# De Novo Atherosclerotic Renal Artery Stenosis Covered Stent Treatment for Resistant Hypertension (ARTISAN) Results

**DOI:** 10.1016/j.jscai.2024.102400

**Published:** 2024-10-18

**Authors:** Douglas E. Drachman, D. Christopher Metzger, Ashit Jain, Ravish Sachar, Amr El-Sayed Abbas, Kenneth Rosenfield, Gary M. Ansel

**Affiliations:** a Massachusetts General Hospital, Boston, Massachusetts; b Ohio Health Heart & Vascular Physicians, Columbus, Ohio; c California Cardiovascular Consultation and Medical Associates, Fremont, California; d North Carolina Heart and Vascular, Raleigh, North Carolina; e William Beaumont Hospital, Royal Oak, Michigan; f The University of Toledo, Toledo, Ohio

**Keywords:** covered stents, hypertension, iCast RX stent system, renal stenting, renovascular hypertension, restenosis

## Abstract

**Background:**

Atherosclerotic renal artery stenosis (ARAS) may provoke hypertension and/or impaired kidney function. Some patients develop uncontrolled hypertension and deteriorating kidney function despite optimal medical therapy. In these patients, endovascular treatment is an important therapeutic option. ARTISAN was a prospective, open-label, single-arm, multicenter clinical trial to evaluate the safety and effectiveness of the iCast RX covered stent both functionally for reestablishing renal artery flow, and clinically for controlling resistant hypertension.

**Methods:**

Patients considered for enrollment had average systolic blood pressure (SBP) ≥155 mm Hg despite taking 3 antihypertensive medications. Prior to enrollment and covered stent placement, angiographic confirmation of ARAS ≥80% with physiologic significance was required. Clinical assessments were performed at 30 days, 9 months, and annually through 36 months. Covered stent safety and efficacy were based on 9-month coprimary end points, including primary vessel patency and SBP improvement at 9 months. Secondary outcomes included target lesion revascularization, major adverse events, and secondary patency.

**Results:**

Sixty-eight of the planned 138 subjects were enrolled. Primary patency was seen in 94.3% of subject lesions; the mean SBP reduction was 15.7 mm Hg. The functional and clinical end points met prespecified performance goals of 70% primary patency (*P* < .0001) and ≥10 mm Hg SBP decrease (*P* = .0192), respectively, at 9 months. Six subjects (8.8%) experienced 7 major adverse events within 36 months. The clinically driven target lesion revascularization rate was 7.3% at 36 months.

**Conclusions:**

The high primary patency and improvement in SBP, persisting through 36 months, suggest that the iCast RX covered stent is safe and effective for the treatment of appropriately selected patients with ARAS.

## Introduction

Atherosclerotic renal artery stenosis (ARAS) is widely prevalent and is associated with the risk of progressive renal dysfunction and systemic major adverse cardiovascular events. Cardinal manifestations of ARAS include hypertension, kidney disease from ischemic nephropathy, flash pulmonary edema, and congestive heart failure. The pathophysiology of ARAS involves activation of the renin-angiotensin-aldosterone system, with resultant secondary hypertension, which is well-described.^1^ Although control of blood pressure with optimal medical therapy remains the mainstay of management,^2^ individuals with ARAS-related accelerated or resistant hypertension may derive significant benefit from renal artery revascularization, supported by societal guidelines and expert consensus documents.^3^^,^^4^ The multisociety 2018 Appropriate Use Criteria for Peripheral Artery Intervention statement indicates that renal artery stenting may be an appropriate option for treatment of hypertension in individuals with the following circumstances: atherosclerotic severe renal artery stenosis (>70% angiographic diameter stenosis, or 50% to 69% stenosis with hemodynamic confirmation of lesion severity) associated with resistant or uncontrolled hypertension despite the use of 3 antihypertensive drugs at the maximally tolerated doses, one of which is a diuretic agent; or refractory hypertension and intolerance of 3 medications at maximal doses.^5^

The majority of renal artery stent procedures are performed using bare metal stent (BMS) scaffolding. Despite excellent acute procedural success rates, prior investigations have identified that in-stent restenosis (ISR) may occur in 11.4% to 21% of cases between 6 months and 5 years of follow-up, attenuating the long-term efficacy of BMS treatment.^6^, ^7^, ^8^, ^9^

Although coronary drug-eluting stents have dramatically reduced the incidence of restenosis following percutaneous coronary intervention, their use for the treatment of renal artery stenosis has not demonstrated benefit over BMS.^10^^,^^11^ Stent scaffolding covered with expanded polytetrafluoroethylene (ePTFE) membrane has been shown to reduce restenosis following treatment of aortoiliac atherosclerotic stenoses and arteriovenous fistula graft stenoses, with a high degree of technical success.^12^, ^13^, ^14^ Moreover, the ePTFE membrane prevents plaque extrusion through stent interstices during deployment, and may thereby reduce atherosclerotic plaque embolization during deployment. Dilation at the aorto-ostial junction of the renal artery may rarely create dissection extending into the aortic wall; the use of an ePTFE-covered stent may theoretically reduce that occurrence. Although ePTFE-covered stents offer potential advantages for the treatment of ARAS, this approach has not previously been examined. The De Novo Atherosclerotic Renal Artery Stenosis Stent Treatment for Resistant Hypertension (ARTISAN) trial was therefore developed to investigate the impact of renal artery stenting using the iCast RX Covered Stent System (Atrium Medical Corporation) for patients with de novo ARAS.

## Methods

### Study design and logistics

The ARTISAN clinical trial was a prospective, open-label, single-arm multicenter trial designed to evaluate the safety and effectiveness of the iCast RX stent for the treatment of ARAS, both to maintain renal artery patency and to control resistant hypertension. This US Food and Drug Administration-approved investigational device exemption protocol (G110194) has been registered on the National Institutes of Health website (ClinicalTrials.gov; identifier NCT01673373). Enrollment was terminated early due to slowed accrual, a result of changing clinical perspectives regarding the utility of renal artery revascularization. The decision to stop enrollment was made although the investigators, trial leadership, and sponsor remained blinded to study results, and without having performed an interim analysis.

### Ethical statement

Subjects were enrolled in accordance with regulatory guidance for the protection of human participants, following good clinical practice (GCP) and the Declaration of Helsinki regarding investigation in humans. Approval was obtained from local institutional review boards. Informed consent was obtained prior to initiating treatment in accordance with institutional guidelines.

### Data management

Data management, data and statistical analysis, monitoring, management of the clinical events committee (CEC) and data safety monitoring board (DSMB), and generation of the clinical investigation report were performed by clinical research organizations (Prairie Education and Research Cooperative or Covance/Chiltern); with oversight from the sponsor (Atrium Medical Corporation). An independent medical monitor reviewed all potential subjects prior to enrollment (Artemida Pharma Ltd). Data were collected and processed using the MedNet Electronic Data Capture & Data Management System. Independent core laboratories evaluated angiographic (SynvaCor) and duplex ultrasound (VasCore, Massachusetts General Hospital) imaging. The CEC reviewed potential major adverse events (MAE) throughout the study and adjudicated the occurrence of study end points. The DSMB reviewed data related to safety, data integrity, and overall conduct of the study.

### Patient selection

Patients were required to be at least 18 years of age and have presence of unilateral or bilateral de novo ARAS defined by angiographic stenosis ≥80% confirmed by angiography caliper measurement of tightest point of stenosis compared to reference luminal diameter; fractional flow reserve <0.8, or a translesional peak pressure gradient of >21 mm Hg, after induced hyperemia via intraarterial dopamine or papaverine, using a pressure wire or 4F or smaller-caliber catheter, respectively.^15^^,^^16^ Eligible target lesions originated ≤15 mm from the renal artery ostium, and were no more than 16 mm in length, and were in vessels of 5 to 7 mm reference diameter. Patients were required to have an average systolic blood pressure (SBP) ≥155 mm Hg during the screening period, despite clearly documented efforts to control hypertension with optimal medical treatment for at least 3 months prior to screening. The documented treatment history was required to demonstrate at least 1 month of stable use of the maximum tolerated dose of at least 3 antihypertensive medications of different classes, including a diuretic. If documented medication compliance was not available, a medication diary was kept for at least 14 days prior to enrollment to ensure that candidate subjects met this requirement. Exclusion criteria are noted in Supplemental Material 1.

### Device description

The iCast RX Covered Stent System consists of a balloon-expandable covered stent mounted on a balloon dilation catheter (Central Illustration). The stent comprised a 316L stainless steel frame, encapsulated with an ePTFE covering overlying the stent struts on both the interior and exterior surfaces of the endoprosthesis. Available stent sizes range from 5 to 7 mm in diameter with 3 available lengths between 16 to 24 mm. The device is crimped and premounted on a dual-lumen rapid exchange balloon catheter delivery system, which is available in 80 cm and 140 cm shaft lengths. The stent system is compatible with a 0.014-inch guide wire and, depending on stent diameter, may pass through a 5F or 6F guide sheath or a 6F or 7F guide catheter.

**Central Illustration:**
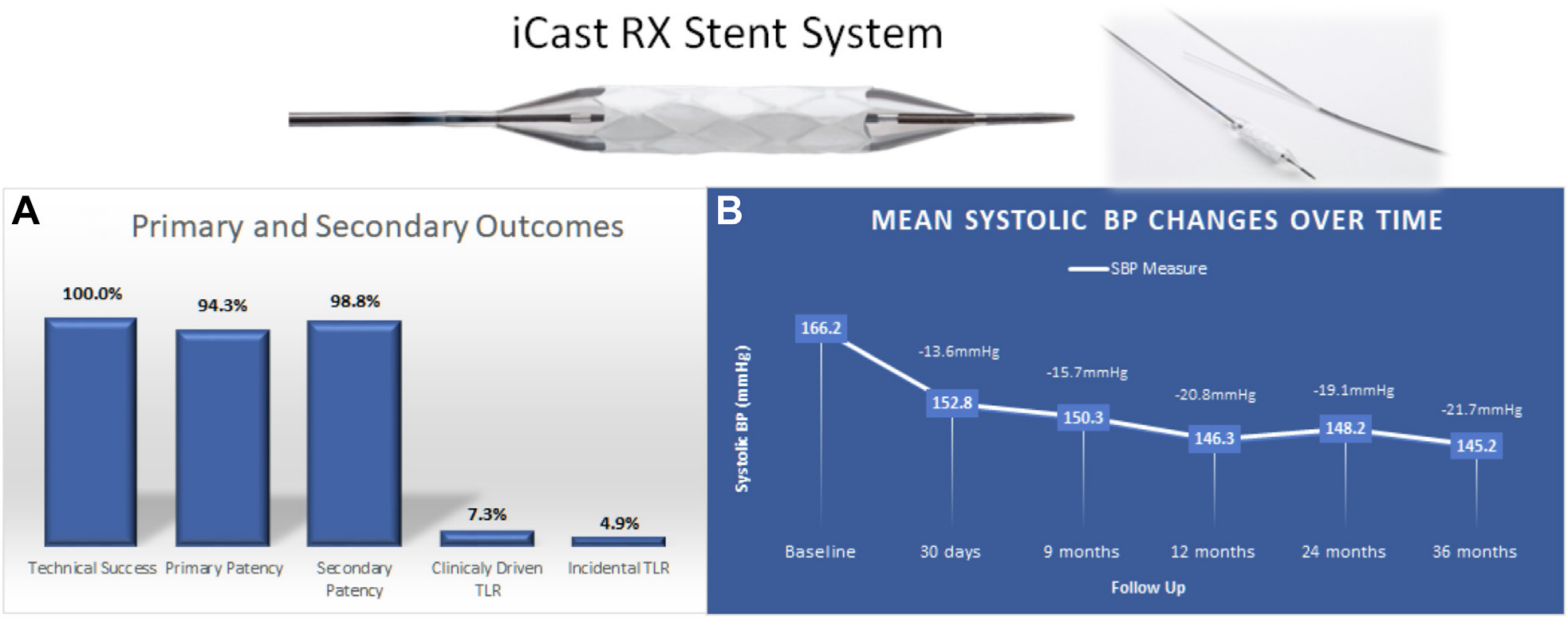
**Primary and secondary outcomes, blood pressure changes, and image of the iCAST RX Covered Stent System.** (**A**) Technical success, primary and secondary patency at 9 months, target lesion revascularization (TLR) rates at 36 months. (**B**) Changes in mean systolic blood pressure (SBP) from baseline, at 30 days, 9 months, 12 months, and 36 months.

### Procedural strategy and follow-up

Baseline and 2-week screening period assessments included demographics, medical history, protocol-guided blood pressure measurements, vital signs, chemistry panel, physical examination, documented clinical evidence of likelihood of renal stenosis (duplex ultrasound [DUS], computed tomography angiography, magnetic resonance angiography), collection of concomitant medications, and adverse events. Prior to enrollment, diagnostic angiography or lesion hemodynamic assessment was used to confirm that all lesions met the protocol criteria. Lesion length and native vessel reference diameter were measured to ensure the selection of the appropriate stent size. The iCast RX stent system was utilized according to the instructions for use provided with the investigational device. Lesion predilation and use of distal embolic protection filters were left to the discretion of the investigator. All enrolled subjects received dual antiplatelet therapy prior to the procedure and for at least 3 months thereafter. A subject was considered enrolled when the study device was introduced into the body. Following the index procedure, clinical assessments, including blood pressure, were performed at 30 days, 9 months, 12 months, 24 months, and 36 months. Serum chemistries were obtained at 30 days and 9 months. Study personnel conducted telephone follow-up assessments at 6 months and 18 months postprocedure. DUS examinations were used to assess treated lesions at the 1- and 9-month visits. A confirmatory angiogram was required if the 9-month DUS was nondiagnostic or suggestive of ≥60% ISR, determined by a core laboratory.

### Blood pressure methodology

The American Heart Association technique for blood pressure measurement was utilized in the study. Blood pressure was measured and recorded in an office setting. The equipment was required to be certified, calibrated, and validated. Rigorous guidelines for subject positioning and activities prior to measurement were followed. A minimum of 3 blood pressure measurements, separated by 2 minutes between measurements, were averaged. If the readings differed by >5 mm Hg, additional readings were obtained and averaged. The initial measurements were taken in both arms and the arm with higher reading was accepted as the study arm for blood pressure measurements for the remainder of the trial. The SBP ≥155 mm Hg hypertension threshold for enrollment and the blood pressure assessment methodology were established in conjunction with the US Food and Drug Administration in developing the investigational device exemption protocol (G110194). Additional methodologic detail and rationale are available for review in the ARTISAN study protocol (Supplemental Material 2).

### Study end points

The efficacy of ARAS treatment using the iCast RX Stent System was assessed via the coprimary end point, which included both functional and clinical parameters; the secondary end points assessed the safety of the treatment. The functional coprimary end point was primary patency of the stent at 9 months, defined as continuous patency without the occurrence of a total occlusion of the original lesion, without a reintervention to treat a partial or total occlusion of the stented segment or bypass of the stented segment due to clinically-driven restenosis or occlusion. The functional end point was compared to a performance goal requiring a primary patency rate of ≥70%. This performance goal was derived from the observed patency rates in prior studies utilizing BMS and percutaneous transluminal angioplasty alone for treatment of renal artery stenosis, with an adjustment to compensate for enrollment of more severe lesions (80% stenosis or greater) than previous studies (see Supplemental Material 3 for the explanation). The clinical coprimary end point was the improvement of SBP at 9 months compared to baseline and was assessed based on a performance goal requiring a decrease in SBP ≥10 mm Hg. Secondary end points included acute technical success and acute procedural success. Technical success was defined as successful delivery and stent deployment, with core laboratory confirmation of ≤30% residual angiographic stenosis at procedure completion. Acute procedural success was defined as technical success without the occurrence of an MAE. The incidence of MAE was defined as the percentage of subjects experiencing procedure- or device-related death, Q-wave myocardial infarction, clinically driven target lesion revascularization (CD-TLR), or significant embolic events (including kidney/bowel infarction identified by symptoms of abdominal or back pain, lower extremity ulceration or gangrene, or kidney failure).

Secondary end points for longer-term outcomes included the rates of target lesion revascularization (TLR) (clinically driven alone, as well as nonclinically driven TLR), MAE, and secondary patency. Additional secondary end points included changes relative to baseline for SBP, renal function (estimated glomerular filtration rate), and use of antihypertensive medications. CD-TLR was defined as the proportion of subjects requiring a TLR due to documented recurrent hypertension and/or deterioration in renal function in association with ≥60% ISR, as assessed by the core laboratory; all cases of CD-TLR were confirmed by CEC adjudication. Potential TLR strategies included percutaneous transluminal angioplasty, BMS implantation, repeat covered stent deployment, or surgical bypass at the discretion of the investigational site.

### Planned sample size

For a 1-sided alpha of 0.025 and desired power of 87%, the sample size for the functional primary patency end point was established by assuming a 0.82 primary patency incidence, which required 125 evaluable subjects. Accounting for 10% attrition, a sample size of 138 subjects was planned. For the clinical SBP reduction end point, the sample size was established by assuming an average reduction of 13.7 mm Hg with a hypothesized SD of 12 mm Hg, resulting in a 92% power to meet the performance goal. This SD was approximated based on the expected range of SBP at baseline of 155 mm Hg to 180 mm Hg and assuming a similar SD at the 9-month follow-up. A mean difference in SBP at 9 months of 12 mm Hg was required to reject the null hypothesis at a 1-sided alpha of 0.025. Given that the coprimary end points of patency and reduction in SBP were powered at 87% and 92%, respectively, this maintained the overall trial power at 80% to meet both end points.

### Statistical analyses

Baseline characteristics, procedural results, and end points were summarized for continuous (n, mean, SD) or categorical (counts and percentage) data, as appropriate. For the functional primary end point, the incidence of 9-month primary patency was calculated by dividing the number of subject lesions achieving primary patency at 9 months by the total number of subject lesions assessed during the protocol allowable 9-month visit window (± 30 days). Only lesions with an evaluable DUS or angiogram performed within the 9-month visit window (± 30 days) were included in the primary efficacy analysis. An exact binomial 1-sided 95% CI was calculated and presented. Similarly, for the clinical primary end point, a 1-sided 95% CI for the mean difference between baseline and 9-month SBP was calculated and presented. The lower limit value of the 95% CI was compared to the performance goal for each of the coprimary end points. Nominal *P* values, without adjustment for multiplicity, were calculated based on the observed differences between the functional and clinical end points and their respective performance goals. *P* values should be interpreted with caution because of the unplanned early termination of the study which raises both issues of multiplicity from interim analysis and reduced power due to the smaller than originally planned sample size. Additional details are available in the statistical analysis plan, Supplemental Material 4.

## Results

### Disposition

**Figure 1:**
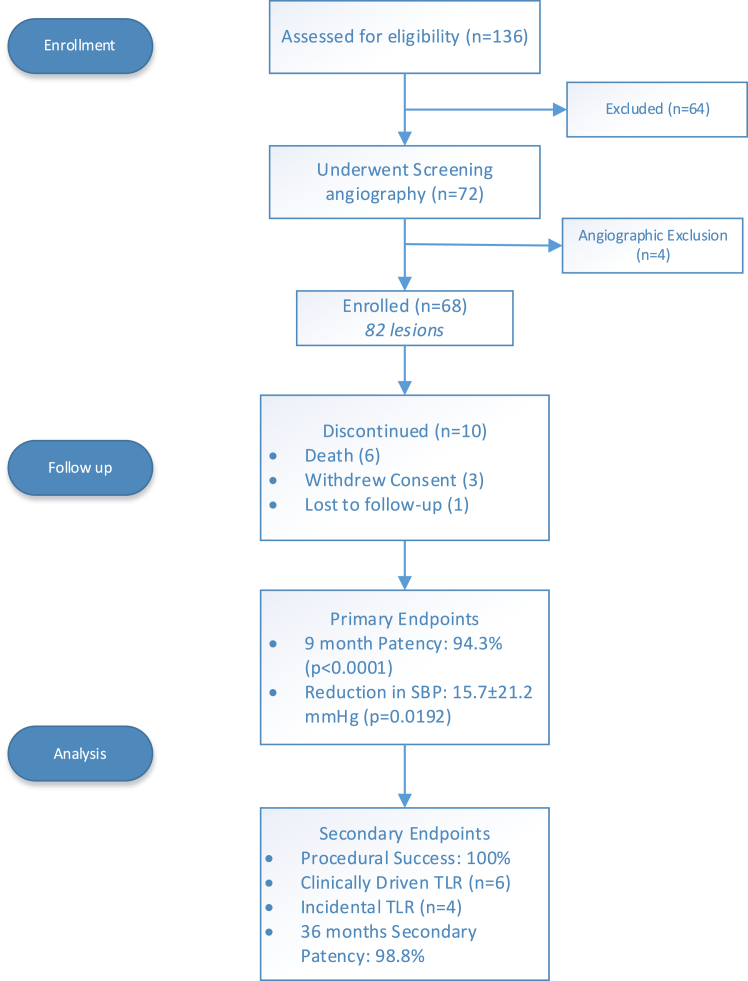
**CONSORT diagram.** SBP, systolic blood pressure; TLR, target lesion revascularization.

From October 2012 through October 2017, 68 of the planned 138 subjects were enrolled. Enrollment was stopped early due to slow enrollment, resulting from changes in the clinical treatment and management paradigm for ARAS, leading to fewer referrals for renal intervention. Enrolled subjects were followed through the 36-month follow-up visit. Ten subjects were discontinued, due to withdrawal of consent (n = 3), loss to follow-up (n = 1), or death (n = 6) (see Figure 1 for further details). Four of the deaths and all 4 of those discontinued due to either withdrawal of consent or loss to follow-up occurred more than 1 year postprocedure. The 6 deaths were unrelated to the study stent and procedure, as determined by the investigator and confirmed by a review of CEC and DSMB committees. The 2 deaths that occurred within 1 year postprocedure were due to hypovolemic shock and cancer. Neither was procedure- or device-related. The 4 remaining deaths occurring more than 1 year following the procedure were due to cancer (n = 1), cardiac failure and arrest (n = 2), and unknown causes (n = 1).

### Demographics, medical history

The mean age of subjects was 71.7 years and more than half of the subjects were female. The mean SBP at baseline was 166.2 mm Hg. A total of 82 lesions were treated in the study, 79.4% characterized as unilateral and 20.6% bilateral. Subjects were treated with concomitant antihypertensive medication as required by protocol. The demographics, baseline characteristics, and medical history of enrolled subjects are presented in Table 1.

**Table 1:** Baseline characteristics and medical history.

	Subjects (N = 68)
Age, y	71.7 ± 10.17
≥65 y	52 (76.5)
Male sex	33 (48.5)
Race	
White	53 (77.9)
Black or African-American	4 (5.9)
Asian	7 (10.3)
American Indian or Alaska Native	1 (1.5)
Other	3 (4.4)
Ethnicity	
Hispanic or Latino	6 (8.8)
Not Hispanic or Latino	62 (91.2)
Body mass index, kg/m^2^	30.3 ± 7.33
Systolic blood pressure, mm Hg	166.2 ± 12.19
Number of lesions	
Unilateral	54 (79.4)
Bilateral lesions	14 (20.6)
At least 1 medical condition	68 (100)
Previous treatment for atherosclerotic renal artery stenosis	4 (5.9)
Renal insufficiency	22 (32.4)
Hyperlipidemia	64 (94.1)
Smoking	
Current/recent	11 (16.2)
Former (stopped >3 months ago)	36 (52.9)
Never smoked	20 (29.4)
Unknown	1 (1.5)
Diabetes	28 (41.2)
Coronary artery disease	46 (67.6)
Other aortic disease	24 (35.3)
Myocardial infarction	12 (17.6)
Congestive heart failure	25 (36.8)
Previous percutaneous coronary intervention	24 (35.3)
Previous coronary artery bypass grafting	12 (17.6)
Peripheral vascular disease	38 (55.9)
Peripheral artery revascularization/surgery	23 (33.8)
Arrhythmia	17 (25.0)
Stent/stent graft	14 (20.6)
Angioplasty	11 (16.2)
Stroke	10 (14.7)
Transient ischemic attack	6 (8.8)

Values are mean ± SD or n (%).

Additional details regarding the determining of the medical history are available in Supplemental Material 5.

### Procedural characteristics

The 68 enrolled subjects received 82 iCast RX stents; 14 subjects received bilateral stents. The majority (78/82) were implanted during the index procedure. Four stents were implanted in a second, planned staged procedure. Preprocedure and postprocedure percent diameter stenoses were 85.7% ± 6.19% and 4.3% ± 6.85%, respectively (Table 2). Two investigator-reported device performance issues occurred during implantation, neither of which met the definition of study device failure or malfunction, including rupture of the stent delivery balloon in 1 case.

**Table 2:** Procedural characteristics.

	Lesions (N = 82)
Preprocedure diameter stenosis, %	85.7 ± 6.19
Number of stents implanted per subject	
1	54 (79.4)
2	14 (20.6)
Stent diameter implanted^a^	
5 mm	17 (21.0)
6 mm	50 (61.7)
7 mm	14 (17.3)
Stent length implanted^a^	
16	49 (60.5)
21	27 (33.3)
24	5 (6.2)
Final poststent diameter stenosis, %	4.3 ± 6.85
Total treated length, mm	15.3 ± 4.66

Values are mean ± SD or n (%).

a Stent information missing for 1 subject lesion.

### Primary end point results

The primary patency evaluation at 9 months was available for 70 of the 82 lesions treated. Of the remaining 12, 4 lesions did not undergo DUS evaluation. The remaining 8 lesions had DUS measurement outside of the 9-month visit window (± 30 days) and were therefore excluded from the patency analysis, although they were all ultimately determined to be patent. The primary patency rate for the 70 evaluable lesions at 9 months was 94.3% (66/70) with an exact 1-sided lower 95% CI value of 86%, which was above the protocol-specified performance goal of 70% (*P* < .0001) (Table 3). Of the 4 lesions in 3 subjects that did not achieve primary patency at 9 months, all underwent a CD-TLR. The performance goal for mean reduction in SBP between preprocedure and 9 months postprocedure was met with a 15.7 ± 21.2 mm Hg decrease, with an exact 1-sided lower 95% CI value of 10.3 mm Hg (Table 3; Central Illustration). This was above the protocol-specified performance goal of 10 mm Hg (*P* = .0192).

**Table 3:** Primary end point results.

	N	End point rate	95% lower limit CI (%)	Performance goal (%)	*P* value
Primary patency (%)	70 lesions^a^	66 (94.3)	86	70	<.0001
Systolic blood pressure decrease (mm Hg)	63 subjects	15.7 ± 21.2	10.3	10	.0192

Values are mean ± SD or n (%).

Primary patency is defined as continuous patency at 9 months without the occurrence of a total occlusion of the original lesion, without a reintervention to treat a partial or total occlusion of the stented segment, or bypass of the stented segment due to clinically-driven restenosis or occlusion.

a Based on the total number of subject lesions with duplex ultrasound or angiography assessment performed during the 9-month visit window (± 30 days).

### Secondary end point results

Acute technical and procedural success was evaluated by the core laboratory in 81 of 82 lesions and was achieved in 100% of those lesions (Table 4). A total of 6 subjects (8.8%) experienced 7 MAE within 36 months, with 2 MAE occurring in 1 subject. Six of the events were CD-TLR. The remaining MAE was a late occlusion of the renal artery detected at 700 days and was associated with an atretic kidney; repeat revascularization was not attempted. Of the 6 lesions (7.3%) requiring CD-TLR within 36 months, 3 TLR (3.7%) occurred in 3 lesions (2 subjects) within 9 months of the index procedure. Nonclinically driven TLR (lesions retreated at the investigators’ discretion that did not meet the prospectively defined criteria for CD-TLR) occurred in 2 patients within 9 months, and 2 others within 24 and 36 months, respectively. The overall secondary patency rate was 98.8% (81/82) (Table 4). All subjects undergoing reintervention had successful results.

**Table 4:** Secondary outcomes.

	Subjects (N = 68) Lesions (N = 82)
Early outcomes^a^	
Technical success	81 (100)
Acute procedural success	81 (100)
Major adverse events^b^	
Overall	6 (8.8)
Within 30	0
Within 9 mo	2 (2.9)
Within 12 mo	3 (4.4)
Within 24 mo	6 (8.8)
Within 36 mo	6 (8.8)
Procedure- or device-related death	0
Q-wave myocardial infarction	0
Clinically driven target lesion revascularization	
Within 9 mo	3 (3.7)
Within 12 mo	4 (4.9)
Within 24 m	6 (7.3)
Within 36 mo	6 (7.3)
Target lesion revascularization^c^	
Within 9 mo	5 (7.4)
Within 12 mo	6 (7.3)
Within 24 mo	9 (11.0)
Within 36 mo	10 (12.2)
Significant embolic event	0
Secondary patency post clinically driven TLR	6 (100)
Overall secondary patency	81 (98.8)

Values are n (%).

TLF, target lesion revascularization.

a Based on 81 lesions evaluable by angiography.

b Percentage of major adverse events calculated with respect to enrolled subjects. All other measures are lesion-based.

c Includes clinically driven and nonclinically driven target lesion revascularizations.

Improvement in SBP was observed at 30 days and was maintained through 36 months. There was no change in antihypertensive medications through 36 months or estimated glomerular filtration rate level through 9 months (Table 5).

**Table 5:** Secondary outcomes: Changes from baseline.

	Value	Change from baseline
Systolic blood pressure, mm Hg		
Baseline	166.2 ± 12.2 (67)	—
30 d	152.8 ± 19.7 (68)	–13.6 ± 18.8 (67)
9 mo	150.3 ± 21.8 (64)	–15.7 ± 21.2 (63)
12 mo	146.3 ± 20.43 (62)	–20.8 ± 20.28 (61)
24 mo	148.2 ± 21.73 (59)	–19.1 ± 21.27 (58)
36 mo	145.2 ± 19.79 (53)	–21.7 ± 18.42 (53)
Antihypertensive medications (n)		
Baseline	4.0 ± 1.1 (68)	—
30 d	3.9 ± 1.2 (68)	–0.1 ± 0.7 (68)
6 mo	3.9 ± 1.3 (67)	–0.0 ± 0.8 (67)
9 mo	3.9 ± 1.2 (65)	–0.1 ± 0.8 (65)
12 mo	3.8 ± 1.3 (64)	–0.2 ± 0.9 (64)
18 mo	3.7 ± 1.2 (61)	–0.3 ± 0.9 (61)
24 mo	3.9 ± 1.5 (59)	–0.1 ± 1.2 (59)
36 mo	3.5 ± 1.3 (59)	–0.4 ± 1.1 (59)
eGFR, mL/min/1.73 m^2^		
Baseline	62.4 ± 23.7 (68)	—
30 d	62.1 ± 22.7 (67)	–0.55 ± 8.4 (67)
9 mo	60.2 ± 22.3 (65)	–2.0 ± 13.4 (65)

Values are mean ± SD (n).

eGFR, estimated glomerular filtration rate.

## Discussion

ARAS is the most frequent etiology of secondary hypertension. The presence of clinically significant stenosis may cause hypoperfusion of the kidney, leading to resistant or refractory hypertension, progressive decline in renal function, and potential development of cardiac syndromes such as pulmonary edema, recurrent heart failure, or acute coronary syndromes. These circumstances may occur despite the utilization of guideline-directed medical therapy. Treatment of renal artery stenosis with stent-based revascularization offers promise to improve these potential clinical sequelae in appropriately selected patients. Procedural complications such as atheroembolism, contrast nephropathy, or longer-term need for repeat revascularization due to ISR may attenuate the short and long-term clinical benefits. In the ARTISAN clinical trial, the effect of renal artery stenosis treatment using an ePTFE-covered stent was evaluated. Covered stents comprise a metal scaffold wrapped by an ePTFE covering, which may attenuate intimal hyperplasia and consequent ISR; further potential benefits may include reduction of intrastrut plaque protrusion (more common for ostial plaque that extends in from the aortic wall) which in turn could precipitate embolization of atheroma. The ARTISAN study was designed to evaluate this unique covered stent platform in patients using very strict criteria to define the presence of the following: (1) significant renal artery stenosis, (2) uncontrolled hypertension, and (3) maximum tolerated antihypertensive therapy. It is this triad that likely identifies individuals with the greatest capacity to benefit from renal artery revascularization.

Over the past 2 decades, clinical trials comparing revascularization to optimal medical therapy for renal artery stenosis have not conclusively demonstrated benefit. Each of these studies was hampered by major methodologic shortcomings. The DRASTIC study enrolled 106 subjects with modest angiographic ARAS of >50%, treated with balloon angioplasty (no stent), with subsequent restenosis in 50% of those treated. Of interest, DRASTIC investigators enrolled on average 2 patients per site over the 4-year course of the trial, an era where renal stenting was commonly performed; selection bias may have limited enrollment of those patients most likely to benefit from therapy.^17^ In the ASTRAL trial, enrollment was inherently biased, as the trial schema explicitly instructed to enroll patients “if the patient’s doctor was uncertain that the patient would definitely…benefit from revascularization.” Patients who would clearly benefit from renal artery revascularization were likely treated outside of the randomized trial, evidenced by the fact that participating centers enrolled an average of only 2 patients per year over the 7 years of the trial. Moreover, enrollment was dependent on DUS assessment for renal artery stenosis, and core laboratory analysis demonstrated that 2 out of 5 enrolled had <70% angiographic stenosis and 1 of 5 patients randomized to the revascularization arm of the study had angiographic stenosis <50%, below the established threshold for treatment.^18^ In the Cardiovascular Outcomes in Renal Atherosclerotic Lesions (CORAL) trial, the inclusion criteria included angiographic severity ≥60%, with average enrolled stenosis of 67%, which is not likely “flow-limiting.”^2^ In addition, during the latter years of CORAL, the angiographic criteria were relaxed substantially to allow enrollment based on the less accurate modalities of computed tomography angiography and magnetic resonance angiography, which are known to occasionally overestimate lesion severity.

These potential shortcomings were addressed proactively by the rigorous design and strict conduct of the ARTISAN trial. In contrast to the prior studies of renal artery revascularization for ARAS, the inclusion criteria for ARTISAN were far more rigorous. Subjects were required to have at least 80% angiographic stenosis, or physiologic demonstration of flow-limiting stenosis in order to be enrolled, enhancing the likelihood that ARAS played a causal role in hypertension in these patients. Furthermore, if patients undergoing evaluation for the potential treatment of renal artery stenosis have not been managed with maximally tolerated medical therapy, including 3 or more antihypertensive agents with at least 1 being a diuretic, it cannot be said that they have failed medical therapy, or presumed that they may derive benefit from renal artery revascularization for hypertension. Patients enrolled in the ARTISAN trial had demonstrated hypertension (average SBP >155 mm Hg) and maximally tolerated medical therapy, according to the rigid criteria described. In the spectrum of clinical trials examining interventional therapies for hypertension, the criteria applied for enrollment in ARTISAN were far more stringent than in previous renal stenting trials.

The ARTISAN trial was originally designed to include 138 patients, but after the publication of the CORAL trial in 2014, enrollment in ARTISAN fell dramatically as many clinicians’ perspectives regarding benefit of renal artery stenting became less favorable. Given the slowed accrual, it was estimated that completion of the trial as designed would require an additional 4 to 5 years. Blinded to the outcomes, the decision was made to stop enrollment. The results garnered from these highly screened patients who failed medical therapy and thus were enrolled in the ARTISAN trial are particularly impactful. Even with far fewer patients than intended, the study demonstrated an important reduction in SBP by more than 15 mm Hg over the 3 years of the study follow-up without the need for additional antihypertensive medications. There were very few complications observed in patients undergoing renal artery revascularization with the ePTFE-covered stent, and the need for CD-TLR was exceptionally low at just over 10% at 3-year follow-up. Compared with BMS, which are commonly associated with restenosis rates in excess of 20% at 1 year, the covered stent platform provided more durable vessel patency, as well as an important and clinically relevant sustained reduction of SBP.

### Limitations

The major limitation of the ARTISAN trial is the fact that enrollment was terminated prematurely, due to slow enrollment. That stated, the trial was terminated prior to any analysis and with all participants blinded to the outcome. Interpretation is challenging given the unplanned nature of the analysis and the reduced power. The study enrolled subjects between October 2012 and October 2017, which came on the heels of the publication of the ASTRAL trial (2009) and was eclipsed by the publication of the CORAL trial (2014). Both of these trials examined medical therapy vs stent-based revascularization of renal artery stenosis and both reported “negative” findings. Although there remain profound methodologic concerns about these studies, the impact of their reported outcomes and the promotion of a noninterventional approach to ARAS markedly reduced the clinical interest in pursuing revascularization for renal artery stenosis. The associated difficulty in enrolling patients in ARTISAN ultimately resulted in the closure of the study. A second limitation of the study is that it was a single-arm, open-label study, and, as a result, covered stents were not randomly compared to BMS or medical therapy alone. The impact of confounding factors, therefore, cannot be excluded. Finally, 1 stent was noted to be totally occluded at 700 days after the index procedure, and the associated kidney was atretic. Although this represents a very low incidence (1.2%) of the treated population, further exploration may be warranted.

## Conclusion

In the ARTISAN clinical trial, the iCast RX Stent was demonstrated to be safe and effective for reducing renal artery stenosis, improving renal perfusion, and controlling resistant hypertension. Covered stent treatment of renal artery stenosis was performed with high technical and procedural success. Although the projected number of subjects were not ultimately enrolled in the study, the primary patency rates and mean SBP reduction met and surpassed the predetermined performance goals. Improvements in SBP and low rates of TLR and MAE were sustained at the 3-year follow-up. Based on the findings of ARTISAN, covered stent treatment of de novo ARAS could be an important treatment option for individuals with refractory hypertension despite maximal antihypertensive therapy.
