# Supplementary material for: De Novo Atherosclerotic Renal Artery Stenosis Covered Stent Treatment for Resistant Hypertension (ARTISAN) Results
Source: J Soc Cardiovasc Angiogr Interv. 2024 Oct 18;3(12):102400. doi: 10.1016/j.jscai.2024.102400 (PMC11725122; doi:10.1016/j.jscai.2024.102400)
Supplement: Supplemental Material 1 [file mmc1.docx]

ARTISAN Supplement 1

### Major Exclusion Criteria

| **General Exclusion Criteria**   - History of kidney or other transplant, polycystic kidney disease, renal aneurysm or organ transplantation, bleeding diathesis or coagulopathy - Known contraindications to anti-coagulants/anti-thrombotics, contrast, stainless steel, PTFE - History of or planned renal bypass or lesion located within or beyond a bypass graft - Thrombolytic treatment in the past 30 days - Stroke or transient ischemic attack in the past 3 months - Significant aortic or valvular disease - Acute pulmonary edema, systolic heart failure with ejection fraction < 30% and/or intubation/ventilation support for this diagnosis in previous 90 days - Hypertensive emergencies resulting in organ damage - An estimated glomerular filtration rate (eGFR) ≤ 25 mL/min/1.73m^2^ - Significant proteinuria >2.0 g/day or a serum creatinine ≥3.0 mg/dL - Ongoing hemodialysis treatment - Presence of cardiogenic shock, cardiomyopathy, active sepsis, NYHA Class IV heart failure, and other uncontrolled concurrent illness - Known bilateral upper-extremity arterial stenosis resulting in spuriously low arm pressures or unable to gain reliable BP measurements from at least 1 arm - Estimated life expectancy < 12 months - Clinically significant disease or circumstance that, in investigator’s opinion, potentially complicating protocol implementation or interpretation of data - Concurrent enrollment in other investigational trial - Other planned/anticipated cardiovascular or interventional surgeries/procedures within 30 days of index procedure and prior to completion of 30-day follow-up - Child-bearing potential and planning to become pregnant during follow-up, or currently pregnant/lactating |
| --- |
| **Anatomic Exclusion Criteria**   - Target lesion is totally occluded or has an anatomic configuration likely to prohibit adequate dilatation, and/or passage or implantation of the investigational device - Multiple ipsilateral lesions of the target renal artery that cannot be covered by a single stent - Previously implanted stent in the target vessel or a previously implanted stent in the contralateral vessel < 1 year - Subject has fibromuscular dysplasia, in renal artery and/or other vascular bed - The target lesion site is associated with a thrombus - Target lesion treated with laser atherectomy, directional atherectomy or other adjuncts to PTA - Critical stenotic (> 70%) small accessory renal artery - Abdominal aortic aneurysm > 4.0 cm in diameter or a severe atherosclerotic aorta - Main renal artery length ≤ 15 mm precluding the safe deployment of a covered renal stent - Any lesion that would include blocking of renal artery side branch - Renal artery stenosis due to dissection of renal artery: spontaneous or traumatic |
