# Supplementary material for: De Novo Atherosclerotic Renal Artery Stenosis Covered Stent Treatment for Resistant Hypertension (ARTISAN) Results
Source: J Soc Cardiovasc Angiogr Interv. 2024 Oct 18;3(12):102400. doi: 10.1016/j.jscai.2024.102400 (PMC11725122; doi:10.1016/j.jscai.2024.102400)
Supplement: Supplemental Material 2 [file mmc2.pdf]

ARTISAN

iCAST™ RX De Novo Stent Placement for the Treatment of Atherosclerotic Renal Artery Stenosis in Patients with Resistant Hypertension

TRIAL SPONSOR

Atrium Medical Corporation
5 Wentworth Drive
Hudson, NH, 03051
U.S.A
Phone: 603-880-1433
Fax: 603-386-6501

Protocol No: iCAST™ RX-ARAS-001

Original Protocol: 22Nov11
Amendment #1: 15Jul13

Confidentiality Statement
The information contained herein is confidential and the proprietary property of Atrium Medical Corporation and any unauthorized use or disclosure of such information without the prior written authorization of Atrium Medical is expressly prohibited.

PROTOCOL SUMMARY

**TRIAL TITLE**	ARTISAN: iCAST™ RX De Novo Stent Placement for the Treatment of Atherosclerotic Renal Artery Stenosis in Patients with Resistant Hypertension
**OBJECTIVE**	The primary objective of the ARTISAN trial is to evaluate the safety and efficacy of the iCAST™ RX Stent System for the treatment of subjects with resistant hypertension associated with de novo atherosclerotic renal artery disease.
**PRIMARY ENDPOINTS**	Functional Endpoint: Assessment of primary patency rate at 9-months, defined as continuous patency without the occurrence of a total occlusion of the original lesion, without a re-intervention to treat a partial or total occlusion of the stented segment, or bypass of the stented segment due to clinically-driven restenosis or occlusion.<br><br>Clinical Endpoint: Improvement in systolic blood pressure (SBP) at 9-months as compared to baseline systolic blood pressure.
**SECONDARY ENDPOINTS**	1. The occurrence of procedure-related Major Adverse Events (MAE) reported as percentage of subjects with MAE, at 30-days, 9-months, 12-months, 24-months, and 36-months. Inclusive of:<br>a. Procedure- or device-related occurrence of death<br>b. Q-Wave Myocardial Infarction (MI)<br>c. Clinically driven target lesion revascularization (TLR)<br>d. Significant embolic events<br>i. Defined as unanticipated kidney/bowel infarct clinically driven by symptoms of abdominal or back pain and confirmed with CT scan or open surgery, lower extremity ulceration or gangrene, or kidney failure after 30-days, 9-months, 12-months, 24-months, and 36-months.<br>2. Technical success defined as successful delivery and deployment of the iCAST™ RX Stent System with ≤ 30%

	residual angiographic stenosis after covered stent deployment (including post-dilatation) assessed via quantitative vascular analysis (QVA) by an independent core laboratory.<br>3. Procedural success defined as technical success without the occurrence of MAE prior to hospital discharge.<br>4. TLR measured as the proportion of subjects that require a clinically-driven reintervention of the target lesion through 9-months.<br>a. A clinically-driven TLR is defined as a TLR (percutaneous balloon angioplasty (PTA), bare metal stent or repeat covered stent deployment, or surgical bypass) due to documented recurrent hypertension from 30-days post-procedure level and/or deterioration in renal function from baseline value, associated with angiographic core laboratory adjudication of a ≥ 60% diameter covered stent restenosis.<br>5. Rate of incidental TLR defined as rate of TLRs not meeting the definition of a clinically driven TLR.<br>6. Improved SBP control assessed at 30-days, 9-months, 12-months, 24-months, and 36-months.<br>7. Secondary patency rate at 9-months after a clinically-driven TLR which restores patency after total occlusion.<br>8. Change in number and dosage of anti-hypertensive medications as compared to baseline.<br>9. Renal function compared to baseline as measured by estimated glomerular filtration rate (eGFR) at 30-days and 9-months.
**TRIAL DESIGN**	This is a prospective, single-arm, multicenter clinical trial that will take place at up to 25 US/OUS sites. Primary endpoints have been determined to show the safety, effectiveness, and clinical outcomes of the iCAST™ RX Stent System. Safety and effectiveness will be evaluated based on the primary patency rate at 9-months on a per lesion basis evaluated against a performance goal of published studies with bare-metal stents. The primary clinical endpoint will assess the improvement in SBP at 9-months as compared to baseline SBP.

	Eligible subjects will undergo a two-week Medical Documentation Screening period to confirm resistant hypertension (SBP ≥ 155mmHg) while on maximum tolerable doses of ≥ three anti-hypertensive medications from at least three classes of drugs, one of which must be a diuretic.<br><br>There must be documented clinical evidence to support likelihood of angiographic findings > 80% whether it is Duplex Ultrasound (DUS), Computed Tomography angiogram (CTa), Magnetic Resonance angiogram (MRa) or other medical evidence. After meeting screening and clinical eligibility criteria, subjects will undergo a baseline assessment for angiographic eligibility. After angiographic documentation of a ≥ 80% diameter renal artery stenosis or Fraction Flow Reserve (FFR) < 0.8 is confirmed, the subject may be enrolled in the trial by placement of the investigational device.<br><br>The 9-month visit will include a follow-up DUS of the target renal artery. If the DUS is non-diagnostic due to an imaging quality problem, such as overlying bowel gas or body habitus, a second DUS may be attempted. If the DUS is indicative of ≥ 60% stenosis as determined by the core laboratory, or the second DUS remains non-diagnostic, a contrast angiogram will be used to assess the degree of restenosis of the covered stent(s).<br><br>Clinical follow-up visits will be required for all enrolled subjects at 30-days, 9-months, 12-months, 24-months, and 36-months. A 6-month and 18-month visit will occur via telephone to collect medication usage and Adverse Events (AEs) only. The 36-month clinic office visit will be required as the final safety visit.
**SUBJECT POPULATION**	Subjects who are candidates for a renal artery revascularization with ≥ 80% visual stenosis and have resistant hypertension with ≥ 155mmHg while on ≥ 3 anti-hypertensive medications from at least 3 classes of drugs, one of which must be a diuretic.
**NUMBER OF SUBJECTS**	Up to 138 subjects will be enrolled in this trial.
**ESTIMATED TRIAL DURATION**	The trial duration is projected for 60 months.

**ESTIMATED SUBJECT DURATION**	Each subject is expected to be enrolled and followed for a total of 36 months.
**STATISTICAL METHODS**	The sample size has been chosen to provide an adequate number of subjects to confirm the safety and efficacy of the iCAST™ RX Stent System. The co-primary endpoints of primary patency and reduction in SBP are powered at 87% and 92%, respectively, in order to maintain the overall trial power at 80% to meet both endpoints with a sample size of up to 138 subjects.
**CO-PRINCIPAL INVESTIGATORS**	[redacted]<br><br>[redacted]
**CONTRACT RESEARCH ORGANIZATION (MONITORING, DATA MANAGEMENT, DSMB, AND CEC)**	[redacted]
**VASCULAR ULTRASOUND CORE LABORATORY**	[redacted]

**ANGIOGRAPHIC CORE LABORATORY**	[redacted]
**MEDICAL MONITOR AND SAFETY**	[redacted]
**ELECTRONIC DATA CAPTURE**	[redacted]
**SPONSOR**	Atrium Medical Corporation<br>5 Wentworth Drive<br>Hudson, NH 03051<br>Phone: 603-880-1433<br>Fax: 603-821-1420

SPONSOR PROTOCOL APPROVAL PAGE

**TRIAL TITLE:**	ARTISAN: iCAST™ RX De Novo Stent Placement for the Treatment of Atherosclerotic Renal Artery Stenosis in Patients with Resistant Hypertension
**INVESTIGATIONAL DEVICE:**	iCAST™ RX Stent System
**PROTOCOL VERSION:**	Amendment #1: 15Jul13
**PROTOCOL DATE:**	Jul 15, 2013
**SPONSOR:**	Atrium Medical Corporation

As the representative of the trial Sponsor, the undersigned have read and agree to oversee the conduct of this trial according to the requirements outlined in this trial protocol and according to applicable FDA regulations (21 CFR Part 812, 50, 54, and 56).

[redacted]

INVESTIGATOR PROTOCOL APPROVAL PAGE

**TRIAL TITLE:**	ARTISAN: iCAST™ RX De Novo Stent Placement for the Treatment of Atherosclerotic Renal Artery Stenosis in Patients with Resistant Hypertension
**INVESTIGATIONAL DEVICE:**	iCAST™ RX Stent System
**PROTOCOL VERSION:**	Amendment #1: 15Jul13
**PROTOCOL DATE:**	July 15, 2013
**SPONSOR:**	Atrium Medical Corporation

Investigator's Responsibility

Prior to participation in the ARTISAN Trial, as the Investigator I understand that I must obtain written approval from my Institutional Review Board (IRB)/Ethics Committee (EC). This approval must include my name and I must send a copy to Atrium or its designated Contract Research Organization (CRO) along with the IRB/EC approved Informed Consent form (ICF) prior to any subject enrollment at my investigational site.

As the Investigator, I must also:

1. Conduct the trial in accordance with the trial protocol, the signed Clinical Investigation Agreement, Good Clinical Practice (GCP), and ensure that all study personnel are appropriately trained prior to any data collection.
2. Ensure that the study is not commenced until IRB/EC approvals have been obtained.
3. Ensure that written informed consent is obtained from each subject prior to any data collection.
4. Provide all required data and reports and agree to source document verification of study data with patient's medical records by Atrium or its designated CRO.
5. Allow Atrium personnel or its designated CRO, to inspect and copy any documents pertaining to this clinical investigation.

Investigator Signature

I have read and understand the contents of the ARTISAN protocol and agree to abide by the requirements set forth in this document.

______________________________	______________________________
Investigator Name (print)	Investigative Site (print)
______________________________	______________________________
Investigator Signature	Date

TABLE OF CONTENTS

1.0 BACKGROUND ... 11
1.1 INVESTIGATIONAL DEVICE ... 13
1.2 INDICATION FOR USE ... 14

2.0 TRIAL OBJECTIVES ... 14
2.1 RATIONALE ... 14
2.2 CO-PRIMARY ENDPOINTS ... 17
2.3 SECONDARY ENDPOINTS ... 17

3.0 TRIAL DESIGN AND POPULATION ... 18
3.1 DETERMINATION OF TRIAL ELIGIBILITY ... 19
3.2 INCLUSION CRITERIA ... 19
3.3 EXCLUSION CRITERIA ... 20
3.4 SUBJECT SCREENING ... 22
3.5 SUBJECT ENROLLMENT ... 25
3.6 SUBJECT DISCONTINUATION ... 25
3.7 SUBJECT WITHDRAWAL ... 25
3.8 EARLY TERMINATION OF THE TRIAL ... 25

4.0 PROCEDURES AND ASSESSMENTS ... 26
4.1 SCREENING PROCEDURES ... 27
4.2 PRIOR TO THE PROCEDURE ... 28
4.3 STENT IMPLANTATION ... 28
4.4 IMMEDIATE POST-PROCEDURE ... 28
4.5 POST PROCEDURE FOLLOW-UP EVALUATIONS ... 29
4.6 CONCOMITANT MEDICATIONS ... 31

5.0 STATISTICAL CONSIDERATIONS AND ANALYSIS PLAN ... 32
5.1 ASSUMPTION OF PRIMARY ENDPOINT RATE ... 32
5.2 SAMPLE SIZE ... 33
5.3 ENDPOINT ANAYLSIS AND REPORTING OF RESULTS ... 35

6.0 ADVERSE EVENTS, SERIOUS ADVERSE EVENTS, AND UNEXPECTED DEVICE EFFECTS ... 38
6.1 DEFINITIONS ... 38
6.2 DEVICE FAILURES, MALFUNCTIONS AND NEAR INCIDENTS ... 39
6.3 DOCUMENTATION ... 40
6.4 EXPEDITED REPORTING OF SAEs AND UADEs ... 41

7.0 TRIAL COMMITTEES ... 42
7.1 DATA SAFETY MONITIORING BOARD (DSMB) ... 42
7.2 CLINICAL EVENTS COMMITTEE (CEC) ... 42

8.0 INVESTIGATOR RESPONSIBILITIES ... 42
8.1 TRAINING ... 42
8.2 PROTOCOLS AND AMENDMENTS ... 43
8.3 INFORMED CONSENT ... 43
8.4 SOURCE DOCUMENTATION REQUIREMENTS ... 43
8.5 PROTOCOL DEVIATIONS ... 44
8.6 DEVICE ACCOUNTABILITY ... 44
8.7 MONITORING ... 45

8.8 AUDITS 45
8.9 RECORD RETENTION 46
8.10 PUBLICATION POLICIES 46

9.0 ROLE OF TRIAL SPONSOR 46
9.1 GENERAL DUTIES 46
9.2 CRITERIA FOR SUSPENDING/TERMINATING AN INVESTIGATIONAL SITE 47

10.0 SUBJECT CONFIDENTIALITY 47

11.0 RISKS TO SUBJECTS 48
11.1 HUMAN SUBJECTS INVOLVEMENT AND CHARACTERISTICS 48
11.2 POTENTIAL RISKS 48

LIST OF APPENDICES

APPENDIX 1: LIST OF ABBREVIATIONS 50
APPENDIX 2: DEFINITIONS 51
APPENDIX 3: METHODOLOGY FOR OFFICE-BASED BP MEASUREMENT 57
APPENDIX 4: SCHEDULE OF EVENTS 58
APPENDIX 5: REFERENCES 59

LIST OF IN-TEXT FIGURES

FIGURE 1: MEDICAL DOCUMENTATION SCREENING PERIOD FLOW DIAGRAM 24
FIGURE 2: TRIAL FLOW DIAGRAM 26

LIST OF IN-TEXT TABLES

TABLE 1: ICAST™ RX STENT SIZES 14
TABLE 2: CONCOMITANT MEDICATIONS 31
TABLE 3: DERIVATION OF PATENCY PERFORMANCE GOAL 34
TABLE 4: DERIVATION OF TLR PERFORMANCE GOAL 37
TABLE 5: ANTICIPATED AES 39
TABLE 6: POTENTIAL AES RELATED TO STENT PLACEMENT 49

1.0 BACKGROUND

Atherosclerotic renal artery stenosis (ARAS) is the most common cause of renal artery stenosis (RAS) in the adult population.[1] ARAS may result in progressive renal impairment, renovascular hypertension, and/or cardiac disturbance syndromes. As the disease progresses, the resulting luminal narrowing decreases blood flow to the kidney, which, in turn, is often accompanied by a reduction in kidney function as well as hypertension triggered by activation of the renin-angiotensin axis. If left untreated, ARAS may progress to renal failure.[2]

The identification and management of patients with suboptimal control of hypertension and coexistent atherosclerotic renal artery stenosis is both challenging and controversial.[3, 4] Current ACC/AHA Peripheral Arterial Disease Guidelines identify renal artery stent deployment as a revascularization strategy in patients with hypertension resistant to optimal medical management.[5] However, a contemporary literature review suggested that there is no data supporting a revascularization strategy over optimal antihypertensive medical therapy.[6] Indeed, several single-arm prospective trials sponsored by commercial device manufacturers, while establishing the procedural safety of renal artery stent deployment after failed/suboptimal balloon angioplasty, were unable to demonstrate a predictable and durable reduction in blood pressure.[7, 8]

More than 50% of subjects with uncontrolled hypertension unresponsive to traditional medical therapy may have significant ARAS and are considered candidates for surgical revascularization or percutaneous intervention for the resolution or stabilization of hypertension and renal dysfunction. Surgical revascularization has proven to be an effective treatment with acceptable long-term patency rates but is hampered by significant morbidity and mortality.[2] Percutaneous transluminal renal angioplasty (PTRA) has been successful in reducing morbidity and mortality compared to surgical revascularization but has poor technical success due to high rates of elastic recoil and dissection for typical ostial lesions, as well as high restenosis rates. Primary stenting of atherosclerotic ostial lesions in the renal artery has thus become the preferred method of treatment due to higher success rates compared to surgical revascularization.[2]

To date, there have been no pivotal clinical trials of blood pressure control or of safety, for newer generation stents in patients with resistant hypertension associated with ARAS. Previously published industry-sponsored trials have not focused on improvement of hypertension control as a primary endpoint.[9] The Cardiovascular Outcomes of Renal Artery Lesions (CORAL) trial[10], a National Institutes of Health (NIH) sponsored, prospective randomized controlled trial of optimal medical therapy compared to optimal medical therapy and renal artery stent deployment, will define the potential impact of renal artery stenting on rates of cardiovascular related morbidity and mortality, progression to renal replacement therapy and blood pressure control over five years of follow-up. However, the final analysis of this 1,050 cohort trial is not anticipated until after 2011. In the interim, clinical trials designed to establish the safety of new

renal stent designs and their effectiveness in improving blood pressure control are needed. In order to facilitate the initiation and completion of single-armed prospective trials investigating the safety and effectiveness of balloon-expandable stents to treat hypertensive patients with ARAS, physicians (VIVA) have used modern clinically relevant data and statistical modeling to develop blood pressure and procedural safety performance goals to be used as a comparator in some single-armed prospective trials.[2]

Although a trend toward a favorable restenosis rate has been noted with the use of stents,
as compared with the with balloon angioplasty alone,[11, 6] restenosis remains the main concern after PTRA stenting. Restenosis often results in symptom recurrence and may progress to occlusion and kidney loss. Thus, early restenosis detection is important to allow for effective secondary intervention; DUS is emerging as a screening modality for RAS.[12,13, 14] Although the value of DUS in RAS screening is well established, its role in assessing the hemodynamic outcome of percutaneous intervention and routine follow-up after successful intervention has not been well evaluated.[15]

A number of recognized conditions can limit the accuracy of intrarenal DUS in RAS evaluation, such as multiple (more than two) renal arteries or only mild to moderate stenosis.[13] Patients who are young or have highly compliant arteries may have an absent early systolic peak, especially when distal interlobar arteries are sampled, which may yield false-positive results.[16] Conversely, noncompliant vessels may not display the parvus-tardus response and may result in false-negative results.[17] Several extraneous factors may also affect the accuracy of intrarenal DUS including valvular heart disease, left ventricular contractility disorders, and the effect of vasoactive drugs.[15]

Clinical follow-up, which has traditionally been used in large series of percutaneous and surgical renal artery revascularizations, may not enable detection of asymptomatic restenosis before it becomes symptomatic or progresses to kidney loss. Magnetic resonance angiography (MRa) after stent placement is limited by the ferromagnetic artifacts caused by most stents.[18] DUS as a follow-up modality offers several advantages: being widely available, non-invasive, relatively inexpensive, and well tolerated by patients as it does not require iodinated contrast media. DUS in the evaluation of initial patency and subsequent surveillance after renal artery revascularization has been recommended.[19, 20, 21]

Renal artery angioplasty and stent placement are generally well-tolerated procedures. As compared to stand-alone balloon angioplasty, renal artery stenting is associated with improved immediate results and a decrease in the rate of restenosis.[22] Complications include contrast-induced nephropathy, thrombosis, distal embolization, renal artery dissection, and perforation.[17] Distal protection devices are attractive for safeguarding against the effects of distal embolization and are currently under investigation.[6] However, the short length of renal arteries and lack of a safe landing zone for placement of distal protection devices is a limiting factor. An alternative

strategy could be the use of covered stents.

The use of polytetraflouroethylene (PTFE) covered stents is designed to combine the effectiveness of surgical treatment with the minimal invasiveness of endovascular treatment.[23] The stent-associated PTFE derived graft layer creates a mechanical barrier between the arterial lumen and wall, theoretically improving patency.[24] This method prevents exposure of the vessel wall to constituents of blood that may initiate the intimal hyperplastic process which may reduce the incidence of in-stent restenosis.[24] Lakshminarayan et al suggests that because covered stents could potentially exclude thrombus from within the lumen of lesions with large thrombus burden, there may be a role for these stents in reducing distal embolization.

1.1 INVESTIGATIONAL DEVICE

The iCAST™ RX Stent System consists of a balloon-expandable covered stent and a balloon dilation catheter. The stent is constructed out of 316L surgical stainless steel. The one piece stent cover is constructed out of a tubular form of expanded polytetraflouroethylene (ePTFE). The covering is attached in a manner such that both the inner lumen and outer surface of the stent are encapsulated with the ePTFE cover material. The stent is radio-opaque under fluoroscopy. The covered stent is mounted and crimped onto the dual lumen rapid exchange balloon catheter (delivery system) so that the system is ready for use by the clinician.

The balloon catheter shaft is comprised of two main sections: a distal coaxial section comprised of a thermoplastic inner tube and thermoplastic outer tube and a proximal section comprised of a 304 stainless steel hypo tube assembly. The proximal end of the coaxial section is attached to the distal end of the hypo tube. A non-compliant thermoplastic dilatation balloon is attached onto the distal aspect of the coaxial catheter section. Radiopaque markers are affixed to the inner tube of the distal catheter section to identify the dilatation zone of the balloon. The distal catheter tip is tapered to facilitate advancement of the catheter to and through the stenosis. The proximal hypo tube assembly section of the catheter is comprised of a stainless steel hypo tube with an overmolded locking female Luer hub attached to one end to allow attachment of a male locking Luer fitting (syringe/inflation device). The attached female Luer hub is molded out of clear plastic to assist the user with purging the device of air during preparation. A strain relief is affixed over the Luer hub and is printed with the device information (stent length, deployed stent diameter, guide wire size, and company logo).

The catheter main lumen (annular space between the inner and outer coaxial tubes and lumen of hypo tube assembly) is used for inflation and deflation of the attached balloon. The secondary lumen (lumen of the inner tube of coaxial assembly), is referred to as the Rapid Exchange (RX) lumen and is used for guide wire introduction. The inner and outer tubes are joined approximately 25cm from the distal tip to form the guide wire access port. The catheter will be available in two working lengths: 80cm and 140cm. Balloons will be available in diameters of 5, 6, and 7mm and in lengths to accommodate the length of each stent. Table 1: iCAST™ RX Stent

Sizes:

Table 1: iCAST™ RX Stent Sizes

	Diameter (expanded)		
Length	5mm	6mm	7mm
16mm	√	√	√
21mm	√	√	√
24mm	√	√	√

1.2 INDICATION FOR USE

The iCAST™ RX Stent System (iCAST RX) is indicated for the improvement in blood pressure control for use as an adjunct to balloon angioplasty of de novo or restenotic lesions (≤ 16mm in length) of the renal artery, with a reference vessel diameter of 5.0mm to 7.0mm to assist in the maintenance of vessel patency.

Refer to the manufacturer's Instructions for Use (IFU) for instructions including: sizing and selection of the stent, preparation of the stent and delivery catheter, introduction and positioning of the stent, deployment of the stent, removal of an unexpanded stent, and emergency withdrawal of an expanded stent.

2.0 TRIAL OBJECTIVES

2.1 RATIONALE

The primary goal of revascularization therapy is the re-establishment and maintenance of adequate blood flow within the renal artery to perfuse the kidney. ARTISAN will investigate the re-establishment of adequate blood flow and normalized pressure in the renal artery and its effect on blood pressure in patients with resistant hypertension. In this single-arm trial, primary endpoints have been determined to show the safety, effectiveness, and clinical outcomes of the iCAST™ RX Stent System. Safety and effectiveness will be evaluated based on the primary patency rate at 9-months on a per lesion basis evaluated against a performance goal of published studies with bare-metal stents. The primary clinical endpoint will assess the improvement in SBP at 9-months as compared to baseline SBP.

The primary functional endpoint of primary patency has been established since the clinical care of the physician is considered a more objective measurement of stent performance than the restenosis measurement of DUS alone. In standard practice, a DUS is used to determine patency of a stent. If a DUS is negative and the patient is asymptomatic, no intervention is typically conducted by a physician. If a patient is symptomatic or a DUS is positive, a confirmatory angiogram is typically performed to assess the patency of the vessel.

To properly match the functional outcomes with historical data, the endpoint will be calculated on a per lesion basis. This reflects the current standard of care of treating patients with single

vessel renal artery lesions and those patients with bilateral renal artery stenosis. Analysis of past studies has demonstrated that the number of lesions treated per patient has increased with time.[26] A recent renal trial reported 1.2 lesions per patient were treated.[27] To calculate on a per patient level for this endpoint would bias the results and overestimate the failure rate compared to a per lesion analysis, as more lesions would be at risk for restenosis but would not be accounted for in a per patient analysis.

DUS is an established safe, noninvasive, reproducible, and sensitive method of detecting and following renal artery lesions that provides both morphological and functional assessments of the renal arteries.[22, 28, 29, 30, 31, 32] There are limits with relying exclusively on DUS for evaluating residual stenosis post-renal stenting. Velocity information such as peak systolic velocity (PSV) and renal aortic ratio (RAR) correlate with angiographic stenosis but these DUS criteria, developed for native renal arteries within renal artery stents has not been studied extensively.[25] The extremes of ARAS can lead to a variety of waveform and flow velocity abnormalities and the entire main renal artery must be visualized in order to perform an adequate examination.[24] There are documented technical disadvantages to DUS as it is very operator dependent and as such, is associated with higher technical failure rate than other diagnostic modalities, in part due to the steep learning curve associated with the technique. To mitigate the challenges with DUS interpretation, clinical validation through independent noninvasive core laboratory and/or angiography will be required in order to correctly validate and definitively assess residual percent diameter stenosis. DUS is highly reliable in identifying widely patent stents. However, correlation studies of DUS with angiography have confirmed that DUS is not as accurate when results suggest a stenosis. To mitigate the risk of inaccurate readings, if a stenosis is detected, an alternate means of clinical validation will be used to definitively assess patency.

The primary clinical endpoint of reduction in SBP has been established to determine the benefit of revascularization therapy to perfuse the kidney. The Framingham Heart Study showed that decreasing SBP by 10mmHg can be lifesaving. Additionally, Joint National Committee VII (JNC VII) estimates that a 5mmHg reduction in SBP would reduce the incidence of stroke in patients by 14%, coronary heart disease (CHD) by 9%, and overall mortality by 7%.[33] JNC VII also presented data from a UK diabetes study that demonstrated a 10mmHg decrease in SBP was associated with average reductions of 15% for diabetes-related mortality, 11% for MI, and 13% for microvascular complications of retinopathy or nephropathy.[33] Currently accepted indications for revascularization are significant ARAS with progressive or acute deterioration of renal function and/or severe uncontrolled hypertension; the key point for success is the correct selection of the patient.[34] When performed correctly, renal artery stenting had been shown to stabilize or improve renal function and/or renovascular hypertension in 65-70% of carefully selected patients with ARAS.[1] It is under this guidance that patients with uncontrolled hypertension will be selected by specific angiographic and clinical selection criteria for this trial.

While previous clinical studies have not consistently shown a direct beneficial effect on

hypertension or renal function, revascularization therapy potentially eliminates one of the several factors that contribute to hypertension. This may be supported by the fact that some patients do show a stabilization or improvement in their hypertension following revascularization. Clinical data show that successful renal artery stenting permits reintroduction of ACE inhibitors in some patients which in turn leads to satisfactory maintenance of renal function.[35] Current clinical experience does not allow one to predict exactly which patients may experience a clinical benefit from renal artery stenting. It is hypothesized that revascularization therapy for hemodynamically significant lesions may facilitate the stabilization or reduction of blood pressure in conjunction with ongoing medical therapy for some portion of patients whose blood pressure is otherwise refractory to medical treatment.

The inclusion/exclusion criteria in the ARTISAN trial seeks to target subjects likely to respond positively based on findings observed in the VIVA performance goals for blood pressure treatment. In the VIVA paper, patients are selected specifically for blood pressure improvement based on the review of three prior studies that suggest patients with resistant hypertension with a systolic blood pressure > 155mmHg, have been medically optimized with 3 or more hypertensive medications, and a lesion that is hemodynamically significant (or gradient) will have a likelihood of having a blood pressure improvement beyond medical therapy alone.[36]

The ARTISAN trial will assess the impact of additional clinical outcomes in this patient population such as improvements in systolic blood pressure and renal function as determined by eGFR. Additionally, MAEs, the number and dose of antihypertensive medications used, technical success, acute procedural success, 30-day clinical success, and TLR will be assessed as part of the secondary endpoint analysis. The American Heart Association scientific statement, *Guidelines for the Management of Patients with Peripheral Arterial Disease 2006*, in combination with discussions with expert physicians and data provided from review of previous clinical trials of renal artery stents, were used to select the primary and secondary endpoints as applicable measures of device effectiveness and safety.[37]

Renal artery stent placement has gained increasing acceptance on the basis of historical results of renal angioplasty and the attractiveness of percutaneous compared with surgical revascularization.[38] As a result, primary stenting of lesions has become the standard of care. In this study, primary stenting of the target lesion will be performed where possible. The enrollment criterion of the ARTISAN trial has selected patients with hemodynamically significant stenoses and where necessary, pre-dilatation will be performed due to narrowing of the lumen diameter of the stenosis compared to the size of the device being inserted. This strategy is selected to minimize the risk of distal embolization and stent dislodgment during deployment.

In conclusion, the safety, effectiveness, and clinical benefit of reducing blood pressure with the iCAST™ RX Stent System will be evaluated under the ARTISAN trial design.

2.2 CO-PRIMARY ENDPOINTS

Functional Endpoint

Assessment of primary patency rate at 9-months, defined as continuous patency without the occurrence of a total occlusion of the original lesion, without a re-intervention to treat a partial or total occlusion of the stented segment, or bypass of the stented segment due to clinically-driven restenosis or occlusion.

Clinical Endpoint

Improvement in systolic blood pressure (SBP) at 9-months as compared to baseline systolic blood pressure.

2.3 SECONDARY ENDPOINTS

The secondary endpoints will provide:

1. The occurrence of procedure-related MAEs reported as percentage of subjects with MAE, at 30-days, 9-months, 12-months, 24-months, and 36-months. Inclusive of:
    a. Procedure-or device-related occurrence of death
    b. Q-Wave MI
    c. Clinically driven TLR
    d. Significant embolic events
        i. Defined as unanticipated kidney/bowel infarct clinically driven by symptoms of abdominal or back pain and confirmed with CT scan or open surgery, lower extremity ulceration or gangrene, or kidney failure after 30-days, 9-months, 12-months, 24-months, and 36-months.
2. Technical success defined as successful delivery and deployment of the iCAST™ RX Stent System with ≤ 30% residual angiographic stenosis after covered stent deployment (including post-dilatation) assessed via QVA by an independent core laboratory.
3. Procedural success defined as technical success without the occurrence of MAE prior to hospital discharge.
4. TLR measured as the proportion of subjects that require a clinically-driven reintervention of the target lesion through 9-months.
    a. A clinically-driven TLR is defined as a TLR (PTA, bare metal stent or repeat covered stent deployment, or surgical bypass) due to documented recurrent hypertension from 30-days post-procedure level and/or deterioration in renal function from baseline value, associated with angiographic core laboratory adjudication of a ≥ 60% diameter covered stent restenosis.
5. Rate of incidental TLR defined as rate of TLRs not meeting the definition of a

clinically driven TLR.

6. Improved SBP control assessed at 30-days, 9-months, 12-months, 24-months and 36-months.
7. Secondary patency rate at 9-months after a clinically-driven TLR which restores patency after total occlusion.
8. Change in number and dosage of anti-hypertensive medications as compared to baseline.
9. Renal function compared to baseline as measured by eGFR at 30-days and 9-months.

3.0 TRIAL DESIGN AND POPULATION

This is a prospective, single-arm, multicenter clinical trial that will take place at up to 25 US/OUS sites. Primary endpoints have been determined to show the safety, effectiveness, and clinical outcomes of the iCAST™ RX Stent System. Safety and effectiveness will be evaluated based on the primary patency rate at 9-months on a per lesion basis evaluated against a performance goal of published studies with bare-metal stents. The primary clinical endpoint will assess the improvement in systolic blood pressure at 9-months as compared to baseline systolic blood pressure.

Eligible subjects will undergo a two-week Medical Documentation Screening period to confirm resistant hypertension (SBP ≥ 155mmHg) while on maximum tolerable dose of ≥ three anti-hypertensive medications from at least three classes of drugs, one of which must be a diuretic.

There must be documented clinical evidence to support likelihood of angiographic findings > 80% whether it is DUS, CTa, MRa or other medical evidence. After meeting screening and clinical eligibility criteria, subjects will undergo a baseline assessment for angiographic eligibility. After angiographic documentation of a ≥ 80% diameter renal artery stenosis or FFR < 0.8 is confirmed the subject may be enrolled in the trial by placement of the investigational device.

The 9-month visit will include a follow-up DUS of the target renal artery. If the DUS is non-diagnostic due to an imaging problem, such as overlying bowel gas or body habitus, a second DUS may be attempted. If the DUS is indicative of ≥ 60% stenosis as determined by the core laboratory, or the second DUS remains non-diagnostic, a contrast angiogram will be used to assess the degree of restenosis of the covered stent(s).

Clinical follow-up visits will be required for all enrolled subjects at 30-days, 9-months, 12-months, 24-months and 36-months. A 6-month and 18-month visit will occur via telephone to

collect medication usage and AEs only. The 36-month clinic office visit will be required as the final safety visit.

3.1 DETERMINATION OF TRIAL ELIGIBILITY

Subjects eligible for initial trial screening will present with renal artery stenosis and meet ALL of the inclusion criteria in order to be considered for the ARTISAN trial. If ANY of the exclusion criteria are met, the subject cannot be enrolled in the trial or treated using the investigational device.

The Medical Monitor will provide oversight in the enrollment of subjects during the course of the trial. All potential subjects will be reviewed and approved by the Medical Monitor, prior to enrollment, to ensure the subjects have met all general Inclusion/Exclusion criteria. Once the subject has been reviewed and approved by the Medical Monitor, the subject may proceed to the index procedure.

A subject is deemed enrolled into the trial once all inclusion criteria have been met and reviewed by an independent medical monitor, no exclusion criteria exist, the operator determines the target lesion is ≥ 80% diameter stenosis or FFR < 0.8 and the trial device enters the subject. All adult subjects will be counseled on and instructed to read and sign the required ICF prior to conducting any trial related procedures.

3.2 INCLUSION CRITERIA

Subjects must meet ALL of the inclusion criteria to be considered for the trial:

<u>General Inclusion Criteria:</u>

1. Age ≥ 18 at the time of informed consent.
2. Subject or subject's legal representative have been informed of the nature of the trial, agrees to participate and can comply with study procedures and follow-up, and has signed an IRB/EC approved ICF.
3. Subjects that have bilateral kidneys or a solitary functioning kidney with Renal Artery Stenosis in at least one kidney and an average SBP ≥ 155mmHg.
4. Subject has a history of maximum tolerable dose of ≥ 3 anti-hypertensive medications of different classes, one of which must be a diuretic (for at least two weeks prior to Medical Documentation Screening period).
    a. A documented history for a minimum of 3 months showing reasonable and aggressive efforts to manage hypertension prior to consent. This must include the use of a broad variety of medications that have been used and failed or not tolerated.
5. Subject must have documented clinical evidence to support likelihood of angiographic findings > 80% whether it is DUS, CTa, MRa or other medical evidence..
6. NYHA class I, II, or III the time of trial enrollment.

NOTE: When a subject has bilateral Renal Artery Stenosis both of which require stenting, it is recommended to treat both kidneys with an iCAST™ RX Stent System during the index procedure. In the event that a subject needs a renal stenting procedure staged for renal protection, it is important that the Investigator treats the second renal artery with an iCAST™ RX Stent System after 30 days of the index procedure. If subjects with bilateral stenosis have only one lesion that meets protocol inclusion criteria that lesion should be treated per protocol. The recommendation is to NOT treat the second non-qualifying lesion, however if the operator feels strongly it is indicated, then they should treat per standard of care after 30-days post index procedure in order to comply with exclusion criteria #10.

Subjects with flash pulmonary edema are allowed into the trial should they meet all other Inclusion and Exclusion criteria.

Angiographic Anatomic Inclusion Criteria:

1. Angiographic diameter renal artery stenosis ≥ 80% involving unilateral or bilateral renal arteries.
    a. The degree of percent diameter stenosis for all lesions intended to be treated, must be confirmed via one of the following methods:
        i. Manual or automated measurement with calipers
        ii. Measured FFR < 0.8 using a pressure wire
        iii. Measured translesional peak pressure gradient of > 21mmHg after induced hyperemia via dopamine or papaverine using a 4Fr or less catheter or pressure wire.
    b. Subjects with 60-79% angiographic stenosis who have confirmed FFR < 0.8 may be enrolled.
2. Renal pole-to-pole length ≥ 8cm (per visual estimate).
3. Target lesion length ≤ 16mm per vessel (per visual estimate).
4. Renal artery vessel diameter ≥ 5.0mm and ≤ 7.0mm (per visual estimate).
5. Lesion originating ≤ 15mm of the renal ostium.

3.3 EXCLUSION CRITERIA

Subjects will be excluded if ANY of the following conditions apply:

General Exclusion Criteria:

1. Subject's estimated life expectancy is < 12 months.
2. Subject has a history of transplanted kidney(s), has had another recent organ transplant or polycystic kidney disease.
3. Subject with estimated eGFR ≤ 25mL/min/1.73m^2
4. Subject has a history of bleeding diathesis or coagulopathy or refuses blood transfusions.
5. Subject has a known contraindication to heparin, aspirin, thienopyridine, other anti-coagulant/antithrombotic therapies, contrast media, stainless steel, and/or PTFE.
6. Subject has had a previous renal bypass operation, a bypass is planned, or the target lesion is located within or beyond a bypass graft.

7. Subject has received a thrombolytic agent within the past 30 days.
8. Subject has documented acute pulmonary edema or systolic heart failure with ejection fraction < 30% and/or hospitalization requiring intubation and ventilation support for this diagnosis within the previous 90 days or hypertensive emergencies defined as resulting in organ damage.
9. Concurrent enrollment in any investigational trial wherein subject's participation has not been completed.
10. Subject has had a planned or anticipated cardiovascular surgical or interventional procedure outside of the affected renal artery (including, but not limited to, aortic, renal, cardiac, carotid, femoro-popliteal, and below the knee) within 30 days prior to the index procedure and prior to completion of the 30 day follow–up.
11. Subject has suffered a stroke or Transient Ischemic Attack (TIA) in the past 3 months.
12. Subject is pregnant, lactating, or is of child-bearing potential and plans to become pregnant during the follow-up trial period.
13. Subject with significant valvular disease.
14. Subject with known significant proteinuria > 2+ or > 2.0gm/d.
15. Subject with known bilateral upper-extremity arterial stenosis that result in spuriously low arm pressures or without the ability to gain reliable blood pressure measurements in at least one upper extremity.
16. Subject with active sepsis.
17. Subject with serum creatinine ≥ 3.0mg/dL.
18. Subject with NYHA Class IV at the time of enrollment.
19. Subject is on hemodialysis.
20. Subject has a history of renal aneurysm.
21. Subject with cardiogenic shock.
22. Subject with cardiomyopathy.
23. Subject has an uncontrolled concurrent illness, including but not limited to ongoing or active infection or active autoimmune disease requiring immunosuppressive therapy.
24. Any subject with clinically significant cardiovascular, respiratory, neurologic, hepatic, endocrine, major systematic disease, making implementation or interpretation of the protocol or protocol results difficult or who in the opinion of the investigator would not be a good candidate for enrollment.

Anatomic Exclusion Criteria:

1. The planned site of intervention is totally occluded or has an anatomic configuration likely to prohibit adequate dilatation, and/or passage or implantation of the investigational device.
2. Subject has multiple ipsilateral lesions of the target renal artery that cannot be covered by a single stent.
3. There is a previously implanted stent in the target vessel or there is a previously implanted stent in the contralateral vessel < one year.

4. Subject has fibromuscular dysplasia, in renal artery and/or other vascular bed.
5. The target lesion site is associated with a thrombus.
6. Target lesion treated with laser atherectomy, directional atherectomy or other adjuncts to PTA.
7. Subject has a critical stenotic (> 70%) small accessory renal artery.
8. Subject has an abdominal aortic aneurysm > 4.0cm in diameter or a severe atherosclerotic aorta.
9. Main renal artery length ≤ 15mm precluding the safe deployment of a covered renal stent.
10. Any lesion that would include blocking of renal artery side branch.
11. Renal artery stenosis due to dissection of renal artery: spontaneous or traumatic.

3.4 SUBJECT SCREENING

At each investigational site, a member of the trial research team will approach individual subjects (or their legal representative or guardian) who are potential candidates for participation. A trial research staff member will explain the purpose, procedures, device, and intent of the trial to each potential participant. Interested subjects will be invited to join the trial and asked to provide written informed consent prior to initiation of any trial related procedure.

A focused hypertension medical history will be reviewed and recorded for each subject. History should capture at least three months' information prior to screening to document that reasonable and aggressive efforts were previously made to manage hypertension via a broad variety of medications that have been tried and failed. Potential subjects will have been on a stable medication regimen for a minimum of two weeks prior to beginning the Medical Documentation Screening period. A minimum of a 14-day Medical Documentation Screening period is required to refine case selection and identify subjects most likely to benefit from renal revascularization*. Titration of the subject's antihypertensive medications will not be allowed during this period. The Medical Documentation Screening period will proceed in the following sequence:

1. Potential subjects will complete an initial office visit during which 3 blood pressure measurements will be obtained, utilizing a standardized technique (APPENDIX 3: METHODOLOGY FOR OFFICE-BASED BP MEASUREMENT), to determine an average SBP value. If the average initial SBP value for this visit is ≥ 150mmHg, then the subject will proceed to step 2.

2. The subject will be tested for proteinuria, have labs drawn, can be scheduled for the procedure, and will proceed to step 3.

- Throughout the Medical Documentation Screening period, subjects will complete

a daily questionnaire every day until the day of procedure that will ask if the subject is taking blood pressure medications as prescribed.

- If the subject has recorded daily medication compliance via the completed questionnaire, the subject proceeds to step 3.

3. Subjects will have their BP taken in the office every two weeks prior to the scheduled procedure. Only subjects with an average SBP value ≥ 150mmHg at this visit after 3 sequential blood pressure measurements are taken, utilizing a standardized technique (APPENDIX 3: METHODOLOGY FOR OFFICE-BASED BP MEASUREMENT), will be allowed to proceed.
   - A minimum of two office visits are required within the 14 day Medical Documentation Screening period. If the procedure is scheduled for more than 14 days from the first visit, subjects should return every two weeks (+/- 3days) for BP assessment and monitoring and the daily questionnaire for medication compliance should be continued until the day of procedure.

4. On the day of the scheduled procedure, or no more than 14 days prior, 3 sequential blood pressure measurements will be taken again, utilizing a standardized technique (APPENDIX 3: METHODOLOGY FOR OFFICE-BASED BP MEASUREMENT), to determine an average SBP value. Only subjects with an average SBP of ≥ 155mmHg from the three measurements taken on the last day of the 14-day Medical Documentation Screening period will be considered for enrollment.

*NOTE: If a subject is seen in the clinic with a medical history documenting at least a 1 month stable medication of ≥ three anti-hypertensive medications from at least three classes of drugs, one of which being a diuretic and documented SBP ≥ 155mmHg, they will be allowed to skip the formal Medical Documentation Screening period and to be enrolled as soon as the history and baseline labs are reviewed by the Medical Monitor. Subjects must complete the daily questionnaire from the day of consent through the day of procedure. All other protocol requirements that would have normally occurred during the two week screening period must still be completed even if the screening period is skipped. These assessments include: vitals, BP, Labs, test for proteinuria, physical exam, and a pregnancy test and would need to be completed either on the day of consent or prior to the day of procedure.

Figure 1: Medical Documentation Screening Period Flow Diagram

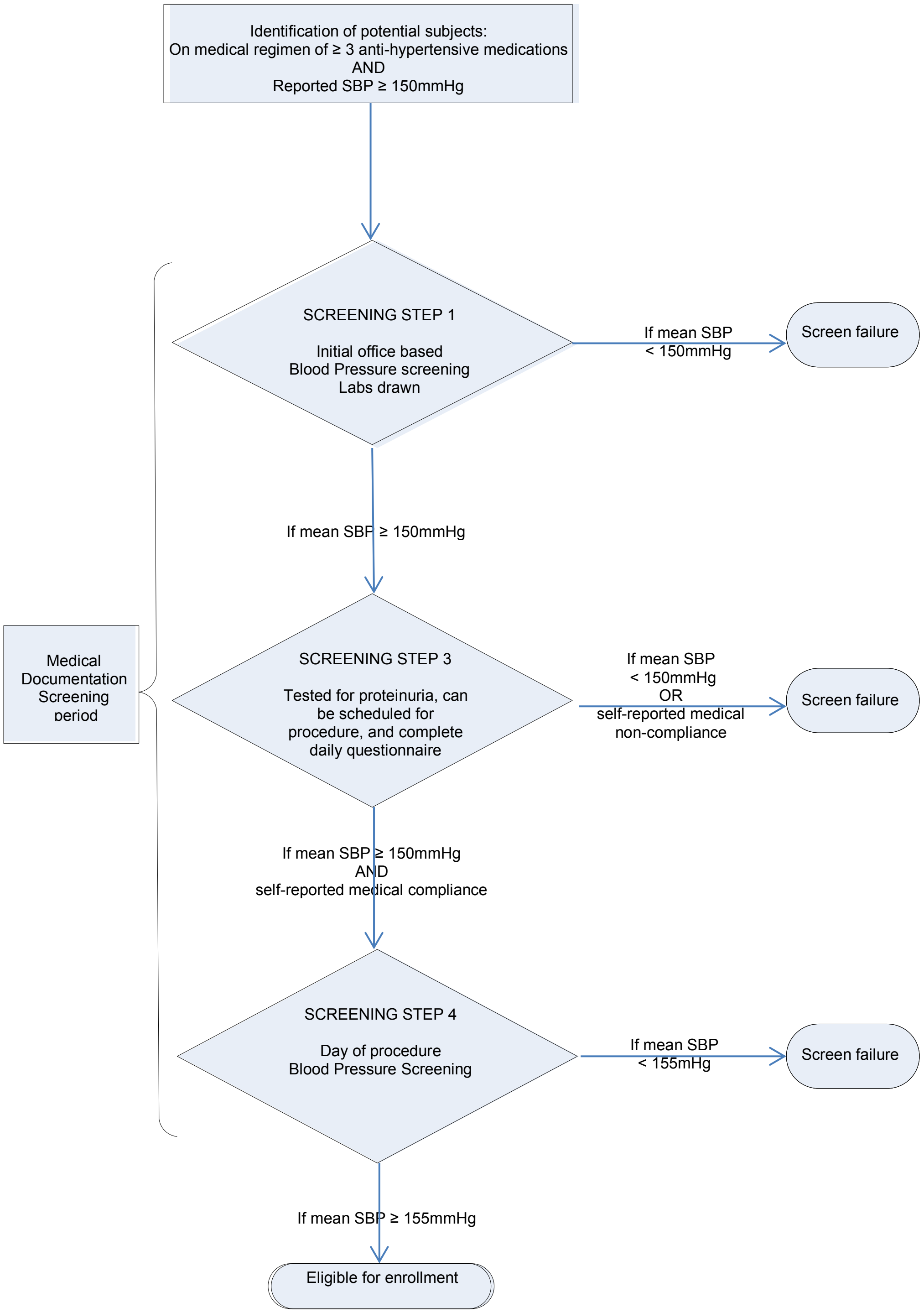

3.5 SUBJECT ENROLLMENT

Up to 138 subjects will be enrolled in this clinical trial. Eligible subjects will be enrolled consecutively at each investigational site. A patient is deemed enrolled into the trial once all inclusion criteria have been met and reviewed by an independent medical monitor, no exclusion criteria exist, the operator determines the target lesion is ≥ 80% diameter stenosis or FFR < 0.8 and the trial device enters the subject. The screening log will then be completed to document the enrollment, subject number, or reason for non-enrollment of subjects screened but not enrolled in the ARTISAN trial.

3.6 SUBJECT DISCONTINUATION

Each enrolled subject shall remain in the trial until completion of the required follow-up period, however, a subject's participation in any clinical trial is voluntary and the subject has the right to withdraw at any time without penalty or loss of standard of care treatment. Conceivable reasons for discontinuation may include, but not be limited to, the following:

1. Subject death
2. Subject voluntary withdrawal
3. Subject withdrawal by physician as clinically indicated

The reason for subject discontinuation must be documented on the electronic Case Report Form (eCRF) and source documents. Investigators must also report all subject discontinuations to their IRB/EC as defined by their Institution's procedure.

3.7 SUBJECT WITHDRAWAL

All subjects should be encouraged to remain in the trial throughout the entire follow up time period. If a subject decides to discontinue trial participation, the reason for their discontinuation must be recorded in the medical record and submitted via the eCRF. Subjects who discontinue participation prematurely will be included in the analysis results if the 9-month visit has been reached.

3.8 EARLY TERMINATION OF THE TRIAL

Possible reasons for early trial termination include:

1. Unanticipated Adverse Device Effects (UADEs) present an unreasonable risk to subjects.
2. The Data Safety Monitoring Board (DSMB) makes a decision for the early termination of the trial per recommendation (refer to Section 7.1).

If the trial is terminated early, Atrium will provide a written statement to the Investigator to enable notification to their IRB/EC.

4.0 PROCEDURES AND ASSESSMENTS

A table summarizing the study procedures is presented in APPENDIX 4: SCHEDULE OF EVENTS. A summary of the assessments to be performed at each interval is presented below.

The following diagram summarizes the timing of visits in this trial:

Figure 2: Trial Flow Diagram

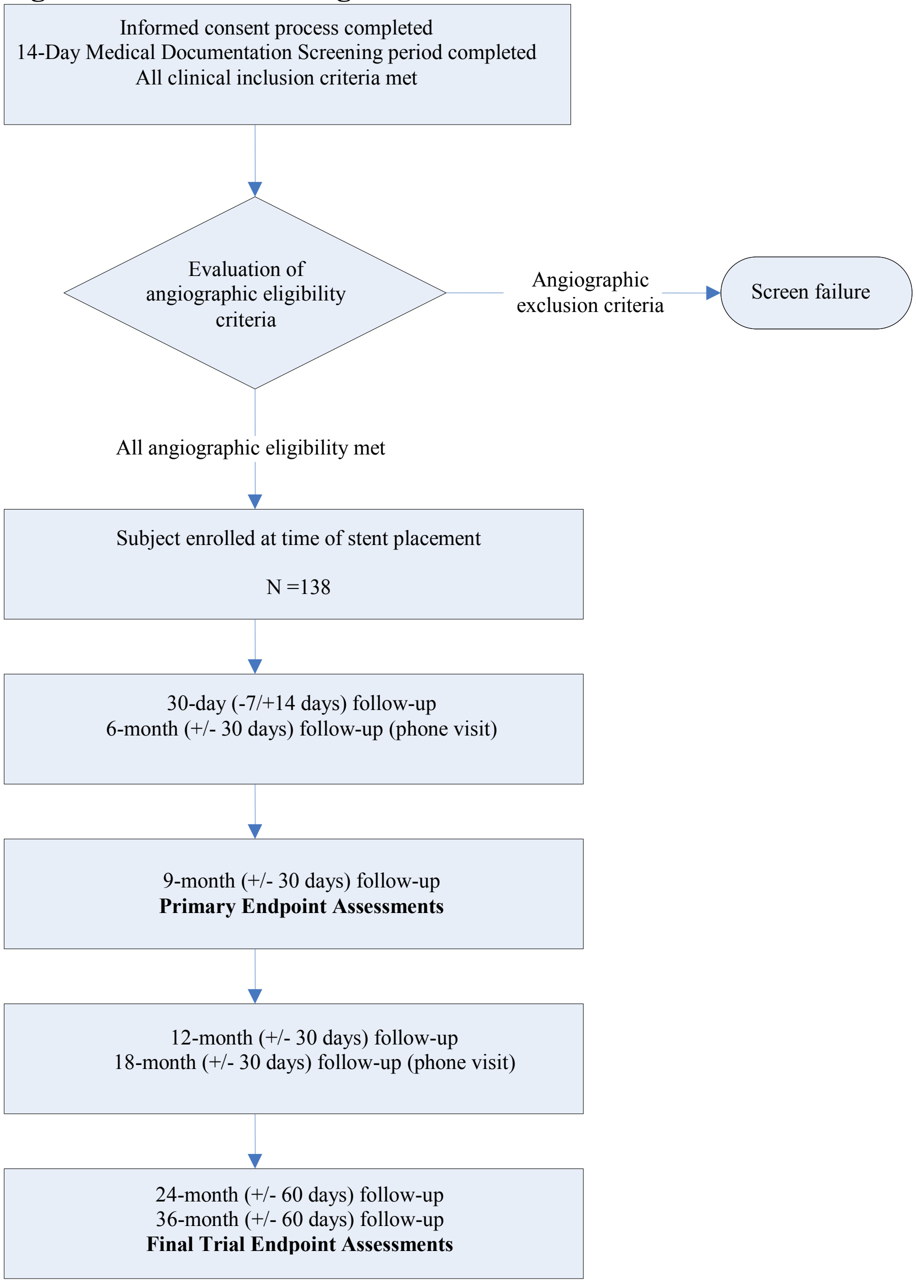

4.1 SCREENING PROCEDURES

At each investigational site, a member of the trial research team will approach individual subjects (or their legal representative) who are potential candidates for participation. A trial research staff member will explain the purpose, procedures, device, and intent of the trial to each potential participant. Interested subjects will be invited to join the trial and asked to provide written informed consent prior to initiation of any trial related procedure.

4.1.1 DAY -14: INITIAL MEDICAL DOCUMENTATION SCREENING VISIT

Trial subjects will complete an initial office visit. The assessment will consist of:

- Signed ICF
- Inclusion/exclusion criteria
- Demographics
- Collection of medical history
- Collection of focused anti-hypertensive medical history
- Height/weight
- Vital signs
- Collection of BP (following the methodology for measurement as outlined in APPENDIX 3: METHODOLOGY FOR OFFICE-BASED BP MEASUREMENT)
- Laboratory assessments
  - Chem12 Panel which includes: Glucose, Calcium, Albumin, Total protein, Sodium, Potassium, CO2, Chloride, BUN, creatinine, ALP, ALT, AST, and bilirubin.
  - Test for proteinuria. Urine proteinuria may be tested by dipstick. A level of > 2+ is exclusionary unless a 24 hour urine shows that the urinary protein is < 2gm/d.
- Physical exam
- Female subjects of childbearing potential will have a pregnancy test
- Documented clinical evidence to support likelihood of angiographic findings > 80% whether it is DUS, CTa, MRa or other medical evidence
- Collection of concomitant medications

4.1.2 DAY -7: MEDICAL DOCUMENATION SCREENING VISIT(S)

Trial subjects will return to the office every 2 weeks prior to the scheduled procedure to monitor their BP. Assessments will consist of:

- Collection of BP (following the methodology for measurement as outlined in APPENDIX 3: METHODOLOGY FOR OFFICE-BASED BP MEASUREMENT)
- Collection of concomitant medications
- Collection of AEs, if applicable

4.2 PRIOR TO THE PROCEDURE

The following tests and examinations must be performed prior to the procedure to verify eligibility.

4.2.1 DAY 0: DAY OF PROCEDURE

Trial subjects will return to the office the day of the scheduled procedure. Assessments will consist of:

- Inclusion/exclusion criteria
- Vital signs
- Collection of BP (following the methodology for measurement as outlined in APPENDIX 3: METHODOLOGY FOR OFFICE-BASED BP MEASUREMENT)
- Laboratory assessments
  - Chem12 Panel
- Physical exam
- Female subjects of childbearing potential will have a pregnancy test
- Collection of concomitant medications
- Collection of AEs, if applicable
- Begin dual anti-platelet therapy (aspirin plus thienopyridine) pre-procedure as required per Table 2: Concomitant Medications

4.2.2 DAY 0: ANGIOGRAPHY

Angiography is to be performed according to standard hospital procedure for renal stent implantation and in compliance with procedures outlined in the Angiographic Core Laboratory Manual of Operations.

4.3 STENT IMPLANTATION

Implantation of the iCAST™ RX Stent System should be performed according to the IFU provided with the investigational device. Refer to the manufacturer's IFU for instructions including: sizing and selection of the stent, preparation of the stent and delivery catheter, introduction and positioning of the stent, deployment of the stent, removal of an unexpanded stent, and emergency withdrawal of an expanded stent.

- Administer IV heparin, low molecular weight heparin, or bivalirudin per routine hospital practice. Target Activated Clotting Time (ACT) must be >250 seconds prior to implantation of the iCAST™ RX Stent System if heparin or low molecular weight heparin is administered. Bivalirudin should be administered in line with the prescribing information according to the weight of the patient.
- NOTE: Pre-dilatation of the lesion and the use of distal protection filters will be at the Investigators discretion and are not distinctly prohibited nor required.

4.4 IMMEDIATE POST-PROCEDURE

Post-procedure, subjects should continue dual anti-platelet therapy (aspirin plus thienopyridine) for a minimum of 3 months except when it is medically necessary to take subjects off therapy.

- Collection of AEs, if applicable

4.5 POST PROCEDURE FOLLOW-UP EVALUATIONS

All trial subjects will be followed through hospital discharge and will undergo follow-up evaluations for a total of 36 months at the following time points:

4.5.1 DAY 30 (+14/-7 DAYS): FOLLOW-UP VISIT

Trial subject follow-up clinic evaluation must occur 30 days post-procedure. The assessment will consist of:

- Vital signs
- Collection of BP (following the methodology for measurement as outlined in APPENDIX 3: METHODOLOGY FOR OFFICE-BASED BP MEASUREMENT)
- Laboratory assessments
  - Chem12 Panel
- Physical exam
- DUS
- Collection of concomitant medications
- Collection of AEs, if applicable
- Subjects remain on dual anti-platelet therapy as required per Table 2: Concomitant Medications

4.5.2 6-MONTH (+/-30 DAYS) PHONE CALL

Trial subject follow-up telephone evaluation must occur 6 months post-procedure. The assessment will consist of:

- Collection of concomitant medications
- Collection of AEs, if applicable

4.5.3 9-MONTH (+/- 30 DAYS) FOLLOW-UP VISIT

Trial subject follow-up clinic evaluation must occur 9 months post-procedure. The assessment will consist of:

- Vital signs
- Collection of BP (following the methodology for measurement as outlined in APPENDIX 3: METHODOLOGY FOR OFFICE-BASED BP MEASUREMENT)
- Laboratory assessments
  - Chem12 Panel
- Physical exam
- DUS*
- *Angiography if DUS indicates ≥ 60% stenosis
- Collection of concomitant medications
- Collection of AEs, if applicable

4.5.4 12-MONTH (+/- 30 DAYS) FOLLOW-UP VISIT

Trial subject follow-up clinic evaluation must occur 12 months post-procedure. The assessment will consist of:

- Vital signs
- Collection of BP (following the methodology for measurement as outlined in APPENDIX 3: METHODOLOGY FOR OFFICE-BASED BP MEASUREMENT)
- Physical exam
- Collection of concomitant medications
- Collection of AEs, if applicable

4.5.5 18-MONTH (+/- 30 DAYS) PHONE CALL

Trial subject follow-up telephone contact evaluation must occur 18 months post-procedure. The assessment will consist of:

- Collection of concomitant medications
- Collection of AEs, if applicable

4.5.6 24-MONTH (+/- 60 DAYS) FOLLOW-UP VISIT

Trial subject follow-up clinic evaluation must occur 24 months post-procedure. The assessment will consist of:

- Vital signs
- Collection of BP (following the methodology for measurement as outlined in APPENDIX 3: METHODOLOGY FOR OFFICE-BASED BP MEASUREMENT)
- Physical exam
- Collection of concomitant medications
- Collection of AEs, if applicable

4.5.7 36-MONTH (+/- 60 DAYS) FOLLOW-UP VISIT

Trial subject follow-up clinic evaluation must occur 36 months post-procedure. The assessment will consist of:

- Vital signs
- Collection of BP (following the methodology for measurement as outlined in APPENDIX 3: METHODOLOGY FOR OFFICE-BASED BP MEASUREMENT)
- Physical exam
- Collection of concomitant medications
- Collection of AEs, if applicable

4.6 CONCOMITANT MEDICATIONS

All medications (prescription, herbal, and over-the-counter) taken from the time of informed consent through 3 years must be recorded in the subject's source records and transcribed into the concomitant medication section of the eCRF.

Subjects are required to receive dual anti-platelet therapy pre-procedure and for a minimum of 3 months post-procedure. The required medications for this study are listed below.

Table 2: Concomitant Medications

Timing	Medication	Procedure
Prior to Procedure	IV Heparin or low molecular weight heparin or bivalirudin	Per routine hospital practice
	Acetylsalicylic acid (ASA)	ASA 325mg loading dose should be given the day of index procedure and prior to placement of the iCAST™ RX Stent System.
	Thienopyridine	The following loading dose of one of the below thienopyridines should be given the day of index procedure and prior to placement of the iCAST™ RX Stent System.<br>Clopidogrel: ≥ 300mg<br>Prasugrel: ≥ 60mg<br>Ticlopidine: ≥ 500mg
During Procedure	IV Heparin or low molecular weight heparin or bivalirudin	Per routine hospital practice
Post Procedure	Acetylsalicylic acid (ASA)	At least 75-325mg per day for a minimum of 3 months
	Thienopyridine	The following maintenance dose of one of the below thienopyridines should be given for a minimum of 3 months or longer based on local guidelines, except when it is medically necessary to take subjects off therapy.<br>Clopidogrel: ≥ 75mg<br>Prasugrel: ≥ 10mg<br>Ticlopidine: ≥ 250mg (twice per day)

5.0 STATISTICAL CONSIDERATIONS AND ANALYSIS PLAN

5.1 ASSUMPTION OF PRIMARY ENDPOINT RATE

The endpoints will be tested in a sequential manner:

1) Functional Endpoint
   Assessment of primary patency rate at 9-months, defined as continuous patency without the occurrence of a total occlusion of the original lesion, without a re-intervention to treat a partial or total occlusion of the stented segment, or bypass of the stented segment due to clinically-driven restenosis or occlusion.

Followed by:

2) Clinical Endpoint
   Improvement in systolic blood pressure (SBP) at 9-months as compared to baseline systolic blood pressure.

If the null hypothesis for the functional goal is rejected at the 0.025 level of significance, the null hypothesis for the clinical goal will be tested at the 0.025 level. If rejected, success will be declared for both Performance Goals. Otherwise, if only the first (primary patency) null hypothesis can be rejected, success will be declared only for that Performance Goal. This sequential approach controls the overall significance level at 0.025. Of note, no success can be declared if the null hypothesis for the primary patency goal was not rejected, regardless of the outcome for the other Performance Goal.

For the primary patency endpoint, the proportion of subjects experiencing this outcome will be compared to the predefined Performance Goal of 0.70. Formally, the hypothesis to be tested is:

H_0: The incidence p_{PP} of primary patency at 9-months is less than or equal to 0.70.

$$H_o:\ p_{PP} \leq 0.70$$

H_A: The incidence p_{PP} of primary patency at 9-months is greater than 0.70.

$$H_A:\ p_{PP} > 0.\ 70$$

The derivation of the Performance Goal is described in Section 5.2.1 Derivation of the Performance Goal for Patency.

For the SBP endpoint, the change between SBP from baseline to 9-month follow-up will be compared to a predefined Performance Goal of 10mmHg. Formally, the hypothesis to be tested is:

H_0: The mean difference between SBP at 9-months and baseline, $\mu_{\Delta SBP}$, is less than or equal to 10mmHg.

$$H_o: \mu_{\Delta SBP} \leq 10\text{mmHg}$$

H_A: The mean difference between SBP at 9-months and baseline, $\mu_{\Delta SBP}$, is greater than 10mmHg.

$$H_o: \mu_{\Delta SBP} > 10\text{mmHg}$$

Both primary endpoints will be tested in one-sided fashion against a significance level of 0.025; the primary patency endpoint will be tested using exact binomial methods and the SBP endpoint will be tested using a paired t-test.

5.2 SAMPLE SIZE

For a one-sided alpha of 0.025 and desired power of 87%, sample size for the primary patency endpoint is established by assuming a hypothesized primary patency incidence of 0.82, which gives 125 evaluable subjects therefore, accounting for 10% attrition, 138 subjects are needed for this endpoint. At the proposed evaluable sample size of 125, no more than 27 subjects (22%) can fail primary patency in order to meet the endpoint hypothesis.

For the endpoint of reduction in SBP, we assume an average reduction of 13.7mmHg. Under a hypothesized standard deviation of 12mmHg for a difference in SBP there will be 92% power to meet the Performance Goal. This standard deviation is conservatively approximated based on the expected range of SBP at baseline of 155mmHg to 180mmHg divided by 3 and assuming a similar standard deviation at the 9-month follow up. A mean difference in SBP at 9-months of 12mmHg will be required in order to reject the null hypothesis at a one-sided alpha of 0.025 at the proposed sample size.

The primary endpoints of primary patency and reduction in SBP are powered at 87% and 92%, respectively, in order to maintain the overall trial power at 80% to meet both endpoints.

5.2.1 DERIVATION OF THE PERFORMANCE GOAL FOR PATENCY

The Performance Goal was derived from a thorough literature review of trials with renal bare metal stent placement. Trials were identified by means of a PUBMED search of the English-language medical literature from 1991 to 2010. When available, the following data were extracted: author and year of publication, number of subjects, number of

arteries, restenosis definition, evaluation method, duration of follow-up, and restenosis rate. Of the 30 articles reviewed, 9 articles were excluded from the Performance Goal based on the following: results from studies with data collection from the 1980's which is not representative of today's standard of care, definitions used for restenosis were outside of our trial design, follow-up occurred at times outside of our assessments, and results from studies not yet published.

The following references were deemed relevant for the patency Performance Goal:

Table 3: Derivation of Patency Performance Goal

Author	Year	Number of Subjects	Number of Arteries	Evaluation Method	Follow-up Time	Restenosis Rate
Rocha -Singh et al[9]	2005	208	252	DUS	9 mo	17.4%
Laird et al[39]	2010	188	188	DUS	9-12 mo	16.8%
Fleming et al[40]	2010	30	66	Angio	6 mo	35%
Corriere et al[41]	2009	91	101	DUS	12 mo	26.7%
Rocha-Singh et al[2]	2008	100	117	DUS	9 mo	21.3%
Nolan et al[42]	2005	78	97	DUS	12 mo	25%
Nolan et al[43]	2005	82	96	DUS	12 mo	25%
Sapoval et al[44]	2005	52	52	Angio	6 mo	14%
Shammas et al[45]	2004	58	58	DUS, Angio, CTa	2-20 mo	26%
Lederman et al[16]	2001	300	358	Angio	16 mo	21%
Symonides et al[7]	1999	27	27	DUS, Angio to confirm	6 mo	30%
Van de Ven et al[8]	1999	42	51	Angio	6 mo	14%
Rundback et al[46]	1998	45	32	Angio	12 mo	25%
Tullis, et al[47]	1997	41	52	Angio/DUS	12 mo	44%
Harden et al[48]	1997	32	33	Angio	6 mo	12%
White et al[49]	1997	100	133	Angio	6-12 mo	19%
Iannone et al[50]	1996	63	86	DUS	12 mo	14%
Dorros et al[51]	1995	76	92	Angio	6 mo	25%
Hennequin et al[52]	1994	21	25	Angio	12 mo	19%

The weighted mean restenosis rate from these trials is 21.4%. Assuming a restenosis rate for PTRA of 40% as noted in Carr based on the analysis of 6 trials with PTRA[(1)] and also used by Rocha-Singh,[(2)] a restenosis rate of 30% leading to a Performance Goal for primary patency set at 70%. This goal will preserve at least 50% of the difference between the currently available bare metal stent rate and the PTRA rate, as well as adjust for a lesion set with a higher percentage stenosis (80%-100%) than previous reported studies (50%-100%).

5.3 ENDPOINT ANAYLSIS AND REPORTING OF RESULTS

5.3.1 GENERAL CONSIDERATIONS

Unless otherwise specified, the following summary statistics will be presented. For continuous variables, statistics will include means, standard deviations and 95% confidence intervals for the means when normal-distribution assumptions are not violated. Otherwise, medians and Interquartile Range (IQR) will be presented. Categorical variables will be summarized using counts and frequencies. For time-to-event data, Kaplan-Meier estimates at the indicated time points will be displayed along with 95% confidence intervals. In addition, survival curves will be constructed for all time-to-event secondary endpoints using Kaplan-Meier methods.

Statistical tests relating to the primary endpoints will be one-sided with p-values less than 0.025 deemed significant.

Statistical analyses will be conducted in Statistical Analysis System (SAS) version 9.1 or above (SAS Institute, Cary, N.C.) or another validated statistical software package.

5.3.2 ANALYSIS POPULATIONS

Intent-to-Treat (ITT) Analysis Set: All subjects enrolled in the study will be included in the ITT analysis set. This will be the primary analysis set.

Per Protocol Analysis Set: All subjects enrolled in the study, who receive the study device as intended with no major protocol violations (such as stent placement/deployment errors) will be included in the Per Protocol analysis set. The primary endpoints will be additionally tested in this analysis set.

5.3.3 MISSING DATA

Results from imputing all possible combinations of successes and failures for the missing data will be summarized.

5.3.4 MULTIPLICITY ADJUSTMENT

Alpha for the primary hypotheses will be controlled by testing the endpoints hierarchically. Patency will be tested first followed by systolic blood pressure. The test for systolic blood pressure will only be conducted if the patency Performance Goal is met.

Two additional secondary endpoints will be evaluated hierarchically if and only if the primary objective is met. In order to control alpha, the endpoint of clinically driven TLR will be tested as described below, followed by changes in the number or dosage of anti-hypertensive medications as compared to baseline.

5.3.5 PRIMARY ENDPOINT ANALYSIS

5.3.5.1 PRIMARY PATENCY

The primary endpoint of primary patency will be summarized using counts and frequencies. An exact one-sided 95% confidence interval will be constructed and the upper limit compared to the Performance Goal of 70%. A p-value for the one-sided test of the difference between the observed rate and the Performance Goal will be presented.

5.3.5.2 SYSTOLIC BLOOD PRESSURE

The primary endpoint of SBP will be analyzed according to the Performance Goal of a decrease in SBP of 10mmHg.

The mean difference between SBP at 9-months and baseline will be compared to a fixed value of 10mmHg. The baseline SBP will be summarized for all subjects and for those subjects with 9-month follow-up data. The baseline and 9-month SBP data, as well as the difference in baseline will be summarized with means and standard deviations. A one-sided 95% confidence interval for the mean difference between baseline and 9-month SBP will be constructed and the limit will be compared to the 10mmHg Performance Goal. A p-value will be computed based on the paired t-test.

5.3.6 ADDITIONAL ANALYSES

5.3.6.1 SENSITIVITY ANALYSIS

For primary patency, a tipping point analysis will be conducted in order to assess the effect of missing data on the analysis. Results from imputing all possible combinations of successes and failures for the missing data will be summarized.

For the SBP endpoint, as a best/worst case analysis, the largest/smallest drop in SBP seen in the complete data will be filled in for the missing values. Summary statistics for the resulting mean difference and p-values from the primary hypothesis test will be presented. In addition, multiple imputations will be used by substituting imputed SBP at 9-months based on available baseline characteristics (including baseline SBP) and then recalculating the mean differences, presenting summary statistics and the p-value for the primary hypothesis.

5.3.6.2 SECONDARY ENDPOINTS ANALYSIS

5.3.6.2.1 MAJOR SECONDARY ENDPOINTS

5.3.6.2.1.1 TLR

The secondary endpoint of clinically driven TLR will be summarized using counts and frequencies. An exact one-sided 95% confidence interval will be

constructed and the upper limit compared to the Performance Goal rate of 8.5% as described below. A p-value for the one-sided test of the difference between the observed rate and the Performance Goal will be presented.

The Performance Goal of 8.5% was established from a review of the relevant literature as detailed in the following table.

Table 4: Derivation of TLR Performance Goal

Author	# subjects	f/u time	TLR Assessment	Subjects with TLR	TLR
Rocha -Singh et al[9]	208	9 mo	9 mo	9	4.3%
Laird et al[39]	188	9-12 mo	9 mo	17	9.0%
Corriere et al[41]	91	12 mo	6 mo	10	11.0%
Rocha-Singh et al2	100	9 mo	9 mo	8	8.0%
Nolan et al[42]	78	12 mo	12 mo	8	10.3%
Nolan et al[43]	82	12 mo	12 mo	8	9.8%
Sapoval et al[44]	52	6 mo	6 mo	4	7.7%
Symonides et al[7]	27	6 mo	6 mo	8	29.6%
Van de Ven et al[8]	42	6 mo	6 mo	0	0.0%
Hennequin et al[52]	21	12 mo	9 mo	4	5.3%
			**Weighted Average**		8.5%

5.3.6.2.1.2 CHANGES IN THE NUMBER OR DOSAGE OF ANTI-HYPERTENSIVE MEDICATIONS

The secondary endpoint of changes in the number or dosage of anti-hypertensive medications as compared to baseline will be summarized using a shift table. The number of patients will be compared to baseline using the paired McNemar's test.

5.3.6.2.2 OTHER SECONDARY ENDPOINTS

All other secondary endpoints will be evaluated in the ITT population using descriptive statistics. No formal hypothesis testing will be performed. The secondary endpoints will be summarized for continuous, categorical or time-to-event data, as appropriate.

5.3.6.3 SUBJECT DISPOSITION, DEMOGRAPHICS AND OTHER BASELINE CHARACTERISTICS

Baseline demographic and clinical variables will be summarized for both the ITT and Per Protocol analysis sets for continuous or categorical variables, as appropriate.

5.3.6.4 SITE-LEVEL POOLABILITY

Poolability of the data across sites will be examined by testing the homogeneity of the primary endpoint results across sites using a random effects model. If differences in the primary or major secondary endpoints exist, these effects will be further examined and results will be additionally reported by adjusting for site using a random effects

model. Since there may be few patients per site and regional differences are expected, this analysis will be repeated for regional differences (US versus OUS). If heterogeneity is detected and the differences between the sites or regions can be attributed to specific differences in baseline characteristics, the random effects model will be further adjusted by these variables. Testing will be conducted at the alpha=0.15 level for this analysis.

5.3.6.5 OTHER SAFETY DATA

Concomitant medications and AEs will be summarized at each follow-up visit. In addition, tables and listings will be provided.

6.0 ADVERSE EVENTS, SERIOUS ADVERSE EVENTS, AND UNEXPECTED DEVICE EFFECTS

In this trial, subjects should be encouraged to report AEs spontaneously or in response to general, non-directed questioning. At any time during the trial, the subject may volunteer information that identifies an AE. If it is determined that an AE has occurred, the investigator should obtain all the information required to complete the AE on the designated eCRF.

6.1 DEFINITIONS

Adverse Events (AE): Any untoward medical occurrence observed in a patient or a clinical investigation subject administered an investigational product and that does not necessarily have a causal relationship with this treatment. An AE can therefore be any unfavorable and unintended sign (including any clinically significant abnormal laboratory finding), symptom, or disease temporally associated with the investigational intervention, whether or not considered related to the investigational intervention. Pre-existing conditions, which worsen during a trial, are to be considered AEs.

Serious Adverse Event (SAE): Any adverse experience that results in any of the following outcomes:

- Death
- Is life-threatening
- Subject hospitalization or prolongation of existing hospitalization
- A persistent or significant disability or incapacity
- Congenital anomaly or birth defect
- Important medical events that may not be immediately life-threatening or result in death or hospitalization, but may jeopardize the subject or may require intervention to prevent one of the outcomes listed in the definition above.

Unanticipated Adverse Device Effect (UADE): Any serious adverse effect on health or safety, or any life-threatening problem or death caused by, or associated with, a device, if that effect,

problem, or death was not previously identified in nature, severity, or degree of incidence in the Investigational Plan or IFU, or any other unanticipated serious problem associated with a device that relates to the rights, safety, or welfare of subjects. A list of anticipated AEs is provided below.

6.1.1 ANTICIPATED AEs

Based on the literature and on clinical and commercial experience with implantation of a covered stent in human arteries, Table 5: Anticipated AEs, includes anticipated AEs that have been identified as possible complications:

Table 5: Anticipated AEs

o Abdominal Pain<br>o Abscess<br>o Acute or sub-acute thrombosis<br>o Acute myocardial infarction<br>o Allergic reaction to stainless steel, drugs, contrast agent, or anti-platelet agents<br>o Aneurysm<br>o Arrhythmias, including Ventricular Fibrillation (VF) and Ventricular Tachycardia (VT)<br>o Arteriovenous (AV) fistula<br>o Bowel infarct<br>o Death<br>o Dialysis<br>o Emboli (air, tissue, or thrombotic) resulting in tissue ischemia or infarction<br>o Emergency surgery to correct vascular complications<br>o Extremity ischemia/amputation<br>o Fever<br>o Hemorrhage<br>o Hematoma	o Hypotension or hypertension<br>o Inadequate implantation or Intimal trauma<br>o Inadvertent exclusion of branch or accessory vessel<br>o Incision site pain or infection<br>o Injury, dissection, perforation, or rupture of the vessel<br>o Kidney infarct<br>o Myocardial infarction or ischemia<br>o Nephrectomy<br>o Pseudo-aneurysm formation<br>o Pyrogenic reaction<br>o Renal insufficiency or failure<br>o Restenosis of stented lesion<br>o Sepsis/infection<br>o Stent embolization<br>o Stent migration/stent misplacement<br>o Stroke or other cerebrovascular accident<br>o Thromboembolic event<br>o Tissue necrosis or ulceration<br>o Total occlusion<br>o Vessel spasm

6.2 DEVICE FAILURES, MALFUNCTIONS AND NEAR INCIDENTS

Investigators are instructed to report all possible device failures, malfunctions or near incidents observed during the course of the trial, to Atrium. In addition, these incidents will be documented in the eCRF provided as follows:

- Device Failure: A device failure has occurred when the device is used in compliance with the IFU, but does not perform as described in the IFU and also negatively impacts treatment of the trial subject.

- Device Malfunction: A device malfunction occurs when an unexpected change to the device that is contradictory to the IFU is observed, which may or may not affect device performance.
- Device Misuse: Any use of the investigational device by an investigator that is contradictory to the application described in the IFU will be categorized as device misuse.

6.3 DOCUMENTATION

All AEs must be listed in the subject's source record, transcribed onto on the appropriate eCRF, and will be characterized by the following criteria:

- Intensity or Severity
- Relatedness
- Outcome
- Treatment or Action Taken
- Expectedness

All AEs (serious and non-serious) will be recorded upon completion of the ICF process through the 9-month follow-up visit. Thereafter, only SAEs (for example, those that result in hospitalization, permanent disability, death, or threat of death) will be recorded. All SAEs will be reported to the CEC and DSMB.

6.3.1 INTENSITY OR SEVERITY

The severity of the AE or SAE should be based on the following categories:

- Mild: An AE that is noticeable to the subject and may require additional therapy and is resolved without treatment and with no sequelae.
- Moderate: An AE that interferes with the subject's activities and requires intervention or additional therapies.
- Severe: An AE that is intolerable, or necessitates additional therapy or places the subject at immediate risk of harm.

6.3.2 RELATEDNESS

The Principal Investigator (PI) will evaluate if the AE or SAE is related to the investigational device. Relatedness is defined in the following manner:

- Not related: The PI has determined that the complication is not related to the trial device.
- Unlikely: The current state of knowledge indicates that a relationship to the use of investigational device is unlikely.
- Possibly or Probably: The PI has determined that the event has a reasonable relationship to the use of the investigational device.
- Definite: The PI has determined that the complication is related to the investigational device.

6.3.3 OUTCOME

The clinical outcome of the AE or SAE will be characterized as follows:

- Death: The SAE eCRF must be completed for this outcome.
- Recovered without Sequelae: Subject returned to baseline status.
- Not Yet Recovered: Subject did not recover and symptoms continue.
- Recovered with Sequelae: Subject has recovered but with clinical sequelae from the event.
- Recovered without Sequelae: Subject returned to baseline status.
- Unknown: Subject outcome is unknown.

6.3.4 TREATMENT OR ACTION TAKEN

AEs or SAEs will result in:

- Interventional Treatment: Surgical, percutaneous or other procedure.
- Medical Treatment: Medication dose reduction/interruption or discontinuation, or medication initiated for event.
- None: No action is taken.

6.3.5 EXPECTEDNESS

- Anticipated: Any AE previously identified as a possible complication in the clinical protocol per Section 6.1.1, the IFU, labeling, or published literature.
- Unanticipated: Any AE not previously identified as a possible complication in the clinical protocol per Section 6.1.1, the IFU, labeling, or published literature.

6.4 EXPEDITED REPORTING OF SAEs AND UADEs

The procedure for reporting any unexpected and related SAEs or UADEs is as follows:

- Report any unexpected and related SAEs or UADEs to Atrium or its designated CRO within 1 business day of knowledge of event by completing the appropriate eCRF forms.
- Report any SAEs or UADEs to the IRB/EC according to the investigational site's IRB/EC procedures.
- Submit physician/nurse notes or discharge summaries related to the reported event, as requested.
- Report of a subject death must be submitted along with a brief statement of the pertinent details and the death records/certificate or autopsy report, if available/performed.

Atrium or its designated CRO will forward site reported unexpected and related SAEs and all UADEs to the regulatory authorities and investigational sites within 10 working days of its receipt. The investigational site will be responsible to forward this report to its IRB/EC.

7.0 TRIAL COMMITTEES

7.1 DATA SAFETY MONITIORING BOARD (DSMB)

The Data Safety Monitoring Board (DSMB) is composed of at least three members which include physicians with specialization in the field of this trial and a biostatistician, and are independent and not directly involved in the conduct of the trial. The DSMB will review the trial on a periodic basis to be defined in the Charter.

Based on the safety data, the DSMB may recommend to Atrium to modify or stop the trial. All final decisions, however, regarding trial modifications, rest with Atrium. No formal statistical rule for stopping the trial will be defined in this trial and no formal interim analysis is planned.

7.2 CLINICAL EVENTS COMMITTEE (CEC)

The Clinical Events Committee (CEC) is made up of physicians with specialization in the field of this trial who are not participants in the trial. The CEC is charged with the development of specific criteria used for adjudication of clinical events related to the primary endpoints in the trial.

At the onset of the trial, the CEC will develop a CEC Charter which will describe explicit rules outlining the minimum amount of data required, and the algorithm followed in order to classify a trial endpoint related clinical event. The CEC will meet regularly (at a minimum of once per year) to review and adjudicate trial endpoint related clinical events in which the required minimum data is not available. The CEC will also review and rule on all deaths that occur throughout the trial.

Once the specific criteria for clinical events and endpoints are established, the CEC will be responsible for adjudicating all clinical events when all necessary data are available.

8.0 INVESTIGATOR RESPONSIBILITIES

The Investigator will ensure the trial will be performed in accordance with the basic principles of the Declaration of Helsinki, ICH E6 Good Clinical Practice (GCP): Consolidated Guidelines, ISO 14155, and any applicable local or governmental regulations. It is the responsibility of each Investigator to provide the current protocol and relevant training to all other staff responsible for trial conduct.

8.1 TRAINING

The training of appropriate investigational site personnel will be the responsibility of the Sponsor, CRO, and PI. The Investigator is responsible for ensuring that his/her staff conduct the trial according to the protocol. The Investigational Plan, instructions on data collection,

schedules for follow-up with the investigational site coordinators, and regulatory requirements will be addressed at the initiation visit. At this visit, Atrium or its designated CRO will also present a formal training session to investigational site personnel that will include review of the device IFU.

8.2 PROTOCOLS AND AMENDMENTS

The protocol and any protocol amendments must be signed and dated by the Investigator and approved by the IRB/EC prior to implementation. No changes to the protocol may be made without the agreement Atrium or its designated CRO. Any amendment to the original protocol will be signed by both parties and submitted to the IRB/EC for approval or notification. Documentation of all study amendment activity should be forwarded to Atrium or its designated CRO.

8.3 INFORMED CONSENT

Prior to participation in the trial, the IRB/EC approved written ICF should be signed and personally dated by the subject or his/her legal representative, and by the person who conducted the informed consent discussion (investigator or designee). If the subject or his/her legal representative is unable to read the ICF, a witness should be present during the entire informed consent discussion. After the ICF is read to the subject and signed by the subject or his/her legal representative, the witness should also sign the ICF, attesting that informed consent was freely given by the subject or his/her legal representative. The informed consent process should be documented in each subject's record.

The subject or his/her legal representative must receive a copy of the signed and dated ICF. The consent form should be updated or amended whenever new information becomes available that may be relevant to the subject.

8.4 SOURCE DOCUMENTATION REQUIREMENTS

Regulations require that Investigators maintain information in the trial subject's medical records which corroborate data collected on the eCRF. In order to comply with these regulatory requirements, the investigator will maintain and make available as required by monitors, Atrium or its designated CRO, and/or its regulatory inspectors the following information that includes but is not limited to:

- Signed ICFs and associated documents.
- Medical history/physical condition of the trial subject before involvement in the trial sufficient to verify protocol entry criteria.
- Dated and signed notes in the subject's medical record on the day of entry into the trial that identify: the subject's date of entry into the trial, the clinical site, subject's assigned number, and a statement that ICF was obtained.
- Dated and signed notes for each trial subject visit with reference to the eCRFs for further information, if appropriate (for specific results of procedures and exams).

- Description of device implantation procedure (material used, drugs administered during the procedure, date, time duration, angiographic, US and clinical findings, etc.).
- Notations on abnormal lab results and their resolution.
- Dated printouts or reports of special assessments, i.e. angiography, echo doppler, CT, X-rays, laboratory tests.
- AE reporting and follow-up of the AEs (minimally event description, severity, onset date, duration, relation to trial device, outcome and treatment for AEs).
- Notes regarding concomitant medications taken during the trial (including start and stop dates).
- Trial subject's condition upon completion of or withdrawal from the trial.

8.5 PROTOCOL DEVIATIONS

A protocol deviation is defined as an event where the Investigator or investigational site personnel did not conduct the trial according to the protocol. Examples of deviations may include, enrollment of a trial subject who does not meet all of the inclusion/exclusion criteria specified in the protocol and missed trial visits.

Atrium will report all deviations annually as part of the IDE annual report to the appropriate regulatory authorities.

Deviations shall be reported to Atrium or its designated CRO regardless of whether medically justifiable or done to protect the subject. Non-subject specific deviations, (e.g. unauthorized use of an investigational device outside the trial, unauthorized use of an investigational device by a physician who has not signed an investigator agreement, etc.), will also be reported to Atrium or its designated CRO in writing. Investigators will also adhere to procedures for reporting protocol deviations to their IRB/EC in accordance with their specific IRB/EC reporting policies and procedures. Regulations require that Investigators maintain accurate, complete and current records, including documents showing the dates of and reasons for each deviation from the protocol.

8.6 DEVICE ACCOUNTABILITY

The Investigator must ensure that the investigational product is used only in accordance with the approved protocol and administered only to subjects under the investigator's supervision or under the supervision of a sub-investigator. It is the responsibility of the Investigator to maintain adequate accountability records of the receipt and disposition of all investigational devices. At the trial closeout visit, the Investigator must return to Atrium or its designated CRO, any unused devices and a copy of the completed device inventory. The Investigator's copy of the device reconciliation report must document any unused devices that have been returned to Atrium or its designated CRO.

8.7 MONITORING

Atrium or its designated CRO will conduct investigational site monitoring visits to ensure that all Investigators conduct the trial in compliance with the protocol and Investigators' agreements. The investigational site will receive notification prior to each monitoring visit during the course of the trial. It is expected that the Investigator and other appropriate staff are available on the day of the visit in case any questions might arise.

The progress of the trial will be monitored by:

- Ensuring the completed eCRF matches the source documents, and resolution of any discrepancies.
- Periodic on-site and off-site review.
- Frequent telephone or electronic communications between the Investigator and site monitor's.
- Review of eCRF and clinical records/source documents. Direct access to complete source documents must be made available during monitoring visits for verification of case report form data.

Periodic monitoring visits will be made at all active investigational sites throughout the clinical trial to assure that Investigator obligations are fulfilled and all applicable regulations and guidelines are being followed. These visits will assure that the facilities are still acceptable, the protocol is being followed, the IRB/EC has been notified of approved protocol changes as required, complete records are being maintained, appropriate and timely reports have been made to Atrium or its designated CRO and the IRB/EC, device and device inventory are controlled, and the Investigator is executing all agreed activities.

Atrium or its designated CRO will retain the right to remove either the Investigator or the investigational site from the trial for issues of non-compliance with the protocol or regulatory requirements. Atrium's designated CRO will fulfill the responsibilities identified in their SOPs.

Atrium or its designated CRO will review significant new information, including UADEs and ensure that such information is provided to the appropriate regulatory authorities, Investigators, and to all reviewing IRBs/ECs.

8.8 AUDITS

On one or more occasions, the investigational site may be inspected or audited by Atrium, Food and Drug Administration (FDA), other local regulatory agency, or a third party. The Investigator may be informed in advance of this visit. Atrium and/or auditors may request direct access to all study records including source documents for inspection and copying, in keeping with country regulations. In the event of an audit by regulatory agency, the Investigator will make all pertinent records available including source documentation for inspection and will immediately notify Atrium or its designated CRO upon notification of any regulatory agency inspections.

8.9 RECORD RETENTION

The Investigator will maintain all essential trial documents and source documentation, in original format, that support the data collected on the study subjects in compliance with the ICH/GCP guidelines. Documents must be retained for at least 2 years after the last marketing application approval or until at least 2 years have elapsed since the formal discontinuation of the clinical investigation of the product. These documents will be retained for a longer period of time by agreement with Atrium or in compliance with other regulatory requirements. When these documents no longer need to be maintained, it is Atrium's responsibility to inform the Investigator. The Investigator will take measures to ensure that these essential documents are not accidentally damaged or destroyed. If for any reason the Investigator withdraws responsibility for maintaining these essential documents, custody must be transferred to an individual who will assume responsibility. Atrium must receive written notification of this custodial change.

8.10 PUBLICATION POLICIES

At the conclusion of the ARTISAN trial, a multi-center manuscript will be prepared for publication in a reputable scientific journal. The publication of the principal results from any single investigational site experience within the trial is not allowed until the preparation and publication of the multi-center results. Exceptions to this rule require the prior approval of Atrium. The analysis of pre-specified and non pre-specified endpoints will be performed by a statistician. Such analyses, as well as other proposed investigations will require the approval of Atrium. For purposes of timely abstract presentation and publication, the writing of these secondary publications will be delegated to the appropriate principal authors, and final analyses and manuscript review for all multi-center data will require the approval of Atrium.

9.0 ROLE OF TRIAL SPONSOR

As the Sponsor of this clinical trial, Atrium has the overall responsibility for the conduct of the trial, including assurance that the trial meets and is conducted within the regulatory requirements specified by each reviewing regulatory authority. In this trial, Atrium will have certain direct responsibilities and will delegate other responsibilities to other designees. Atrium or its designated CRO will ensure adherence to the Sponsor general duties, selection of Investigators, monitoring, supplemental applications, maintaining records, and submitting reports.

9.1 GENERAL DUTIES

Atrium's general duties also include submission of application to the appropriate regulatory authorities and obtaining overall regulatory approval.

Atrium or its designated CRO is responsible for ensuring the ICF is obtained and proper investigational site monitoring is performed, providing quality data that satisfies regulations and informing Investigators of UADE events and deviations from the protocol, as appropriate.

Atrium or its designated CRO will prepare written reports, a final report, and will coordinate data collection and transfer with the core laboratory.

9.2 CRITERIA FOR SUSPENDING/TERMINATING AN INVESTIGATIONAL SITE

Atrium reserves the right to stop the screening and enrollment of subjects at any investigational site at any time for any of the following reasons:

- Noncompliance to GCP/ICH or protocol
- Failure to obtain IRB/EC approval
- Failure to enroll subjects
- Multiple and/or severe protocol deviations
- Inaccurate or incomplete data
- Unsafe or unethical practices
- Safety or performance considerations
- Administrative decision

NOTE: If an investigational site is suspended or terminated, AE assessment and reporting (as outlined in this protocol) will continue for all subjects who received an investigational product.

10.0 SUBJECT CONFIDENTIALITY

Subject confidentiality will be maintained throughout the clinical trial in a way that assures that data can always be tracked back to the source data. For this purpose, a unique subject identification code (i.e. assigned subject number and subject initials) will be used that allows identification of all data reported for each subject.

Data relating to the trial might be made available to third parties (for example in case of an audit performed by regulatory authorities) provided the data are treated confidential and that the patient's privacy is guaranteed.

The Investigator will grant monitor(s) and auditor(s) from Atrium or its designated CRO and regulatory authorities access to the subject's original medical records for verification of data gathered on the eCRF and to audit the data collection process. The subject's confidentiality will be maintained and will not be made publicly available to the extent permitted by applicable laws and regulations.

"Protected Health Information" will be maintained in compliance with the Health Insurance Portability and Accountability Act of 1996 (HIPAA) Privacy rule, where applicable.

11.0 RISKS TO SUBJECTS

11.1 HUMAN SUBJECTS INVOLVEMENT AND CHARACTERISTICS

This trial will enroll 138 adults with de novo atherosclerotic renal artery disease. The inclusion and exclusion criteria are described in Sections 3.2 and 3.3. Vulnerable populations, specifically prisoners, institutionalized individuals will not be targeted for recruitment. Pregnant subjects are excluded because this product has not been investigated in this population and the risk to the fetus is unknown.

11.2 POTENTIAL RISKS

There may be a direct benefit to the subject as the iCAST™ RX Stent System allows for dilatation of the renal artery. This stent has the potential benefits of less injury due to the low profile delivery system and the metal stent is completely encapsulated with the ePTFE covering making it more stable and accurately deployed than other balloon expandable stents of similar diameters.

There are risks for participants in this trial. Potential risks have been minimized by establishing strict inclusion/exclusion criteria to ensure appropriate subjects participate in the trial. Additionally, all enrolling investigators will be instructed on appropriate patient selection, in an effort to minimize the risk of recruiting patients who are found to be ineligible for trial participation.

To minimize the risk of the invasive assessment and the stent procedure, investigators will perform angiography and stent placement based on: current medical license, privileges to perform these services at their treatment center, board certification, or equivalent experience. Appropriate angiographic technique principles will be utilized to expose subjects to the least amount of contrast dye and radiation while providing high resolution angiograms.

Some risks associated with surgery include but are not limited to adverse reactions to anesthesia, DVT, neurological cardiac or respiratory deficit, infection, continuation and/or worsening of the original diagnosis and death. However, it should be noted that the risks of trial participation are not materially different than those entailed by an individual who undergoes a stent procedure with an approved stent. Table 6: Potential AEs Related to Stent Placement outlines possible AEs related to stent placement:

Table 6: Potential AEs Related to Stent Placement

Adverse Event	Anticipated Incidence
Abdominal Pain	< 0.1%
Abscess	< 0.1%
Acute or sub-acute thrombosis	< 0.1%
Acute myocardial infarction	< 0.1%
Allergic reaction to stainless steel, drugs, contrast agent, or anti-platelet agents	< 0.1%
Aneurysm	< 2%
Arrhythmias, including VF and VT	< 0.1%
Arteriovenous (AV) fistula	< 2%
Bowel infarct	< 1%
Death	< 6%
Dialysis	< 5%
Emboli (air, tissue, or thrombotic) resulting in tissue ischemia or infarction	< 4%
Emergency surgery to correct vascular complications	< 3%
Extremity ischemia/amputation	< 0.1%
Fever	< 0.1%
Hemorrhage	< 5%
Hematoma	< 4%
Hypotension or hypertension	< 17%
Inadequate implantation or Intimal trauma	< 5%
Inadvertent exclusion of branch or accessory vessel	< 10%
Incision site pain or infection	< 0.1%
Injury, dissection, perforation, or rupture of the vessel	< 2%
Kidney infarct	< 0.1%
Myocardial infarction or ischemia	< 1%
Nephrectomy	< 2%
Pseudo-aneurysm formation	< 1%
Pyrogenic reaction	< 0.1%
Renal insufficiency or failure	< 6%
Restenosis of stented lesion	< 21%
Sepsis/infection	< 0.1%
Stent embolization	< 0.1%
Stent migration/stent misplacement	< 1%
Stroke or other cerebrovascular accident	< 2%
Thromboembolic event	< 1 %
Tissue necrosis or ulceration	< 0.1%
Total occlusion	< 2%
Vessel spasm	< 0.1%

APPENDIX 1: LIST OF ABBREVIATIONS

ACC	American College of Cardiology
ACE	Angiotensin Converting Enzyme
AE	Adverse Event
AHA	American Heart Association
ARAS	Atherosclerotic Renal Artery Stenosis
ARB	Angiotensin Receptor Blocking agent
ASA	Acetylsalicylic Acid
AV	Arteriovenous
CEC	Clinical Events Committee
CFR	Code of Federal Regulations
CHD	Coronary Heart Disease
CRO	Contract Research Organization
CTa	Computed Tomography angiogram
DSMB	Data Safety Monitoring Committee
DUS	Duplex Ultrasound
EC	Ethics Committee
eCRF	Electronic Case Report Form
eGFR	Estimated glomerular filtration rate
ePTFE	expanded Polytetraflouroethylene
FDA	Food and Drug Administration
FFR	Fraction Flow Reserve
GCP	Good Clinical Practice
HIPAA	Heath Information Portability and Accountability Act
ICF	Informed Consent Form
ICH	International Conference of Harmonisation
IFU	Instructions for Use
IQR	Interquartile Range
IRB	Institutional Review Board
ITT	Intent-to-Treat
MAE	Major Adverse Event
MI	Myocardial Infarction
MRa	Magnetic Resonance Angiogram
NIH	National Institutes of Health
PSV	Peak Systolic Velocity
PTA	Percutaneous Balloon Angioplasty
PTRA	Percutaneous Transluminal Renal Angioplasty
PTRAS	Percutaneous Transluminal Renal Angioplasty with Stenting
QVA	Quantitative Vascular Analysis
RAR	Renal Aortic Ratio
RAS	Renal Artery Stenosis
SAE	Serious Adverse Event
SAS	Statistical Analysis System
SBP	Systolic Blood Pressure
SOP(s)	Standard Operation Procedure(s)
TIA	Transient Ischemic Attack
TLR	Target Lesion Revascularization
UADE	Unanticipated Adverse Device Effect
VF	Ventricular Fibrillation
VT	Ventricular Tachycardia

APPENDIX 2: DEFINITIONS

**ADVERSE EVENT**	Any untoward medical occurrence observed in a patient or a clinical investigational subject. This definition does not imply that there is a relationship between the AE and the device under investigation. An AE can therefore be any unfavorable and unintended sign (including any clinically significant abnormal laboratory finding), symptom, or disease temporally associated with the investigational intervention, whether or not considered related to the investigational intervention. Pre-existing conditions, which worsen during a trial, are to be considered AEs.
**DE NOVO LESION**	A lesion in a native vessel that has not been previously treated.
**DISSECTION**	Renal artery dissections are stenotic or occlusive lesions most often observed in hypertensive patients with underlying atherosclerosis or fibromuscular disease. Acute dissections may present spontaneously, as a complication of diagnostic or therapeutic angiography or as an agonal event associated with overwhelming systemic illness. Chronic dissections may produce renovascular hypertension or be entirely asymptomatic.
**DISTAL EMBOLIZATION**	Free flowing blood clot or thrombus material located in the circulation distal to the treated occlusion.
**DUAL ANTI-PLATELET THERAPY**	The use of thienopyridines in combination with aspirin therapy.
**EMBOLI**	Something that blocks the flow of blood in a vessel. It may be a gas bubble, a blood clot, a fat globule, a mass of bacteria, or other foreign body that usually forms elsewhere and travels through the circulatory system until it lodges in the vasculature.

**ESTIMATED GLOMERULAR FILTRATION RATE**	A measure of kidney function calculated based on the Cockcroft-Gault equation:<br><br>Male:<br>eGFR(mL/min) = (140 - age) x weight (kg)/72 x S_{Cr})<br><br>Female:<br>eGFR(mL/min) = (140 - age) x weight (kg) x 0.85 / 72 x S_{Cr})<br><br>$S_{Cr:}$ Serum creatinine, mg/mL
**FIBROMUSCULAR DYSPLASIA**	Abnormal cellular development or growth in the walls of one or more arteries in the body; most commonly the renal arteries. As a result of abnormal cell development stenosis may develop in these arteries.
**FLASH PULMONARY EDEMA**	A rapid onset pulmonary edema, most often precipitated by acute MI or mitral regurgitation, but can be caused by aortic regurgitation, heart failure, or almost any cause of elevated left ventricular filling pressures.
**HEMATOMA/HEMMORRHAGE**	A swelling or collection of blood, usually clotted, in an organ, space, tissue, or access site due to a break in the wall of a blood vessel. It could also be bleeding into an extravascular space which could be outside the body.
**HYPERTENSIVE EMERGENCIES**	Hypertensive emergencies encompass a spectrum of clinical presentations in which uncontrolled BPs lead to progressive or impending organ damage.
**MAJOR ADVERSE EVENTS**	Procedure-related events inclusive of:<br>• Procedure- or device-related death<br>• Q-Wave MI<br>• Clinically driven TLR<br>• Significant embolic events

**MAXIMUM TOLERABLE DOSE**	The highest dose of treatment that will produce the desired effect without unacceptable toxicity.
**NEW YORK HEART ASSOCIATION CLASSIFICATION**	Class I: The subject has cardiac disease but without resulting limitations of ordinary physical activity. Ordinary physical activity (i.e., walking several blocks or climbing stairs) does not cause undue fatigue, palpitation, dyspnea, or anginal pain. Limiting symptoms may occur with marked exertion.<br><br>Class II: The subject has cardiac disease resulting in slight limitation of ordinary physical activity. Subject is comfortable at rest. Ordinary physical activity such as walking more than two blocks or climbing more than one flight of stairs results in limiting symptoms (i.e., fatigue, palpitation, dyspnea, or anginal pain).<br><br>Class III: The subject has cardiac disease resulting in marked limitation of physical activity. Subject is comfortable at rest. Less than ordinary physical activity such as walking one of two blocks or climbing one flight of stairs causes fatigue, palpitation, dyspnea, or anginal pain.<br><br>Class IV: The subject has dyspnea at rest that increases with any physical activity. Subject has cardiac disease resulting in inability to perform any physical activity without discomfort. Symptoms of cardiac insufficiency or anginal syndrome may be present even at rest. If any physical activity is undertaken, discomfort is increased.
**Q-WAVE MYOCARDIAL INFARCTION**	The diagnosis of MI will be made on the basis of clinical information available from hospitalization (discharge summaries, laboratory data, ECG) and will require an appropriate clinical history consistent with acute MI.

**PATENCY**	Defined and categorized as:<br><br>Primary: continuous patency without the occurrence of a total occlusion of the original lesion, without a re-intervention to treat a partial or total occlusion of the stented segment, or bypass of the stented segment due to clinically-driven restenosis or occlusion.<br><br>Secondary: any procedure that restores patency after occlusion.
**PERFORATION**	The complete penetration of the wall of renal artery; this specifically refers to accidental or pathologic perforation, rather than intentional penetration during surgery.
**PRIMARY STENTING**	Stent placement without prior PTRA, an initial attempt at balloon dilatation, or after intentionally undersized pre-dilatation solely for the purpose of facilitating stent positioning.
**PROCEDURAL SUCCESS**	Technical success without the occurrence of MAE prior to hospital discharge.
**RESISTANT HYPERTENSION**	Failure to achieve goal BP (or SBP ≥ 155mmHg) in patients who are adhering to full doses of ≥ 3 anti-hypertensive medications from at least 3 classes of drugs, one of which must be a diuretic.
**RESTENOSIS**	Recurrent stenosis ≥ 60% diameter luminal narrowing or recurrent translesional gradient, observed post-procedure as determined by the core laboratory. Restenosis is initially assessed by DUS and confirmation of percent diameter stenosis is made by a contrast angiography.
**RESTENOTIC LESION**	A lesion in the vessel segment that has undergone a prior percutaneous treatment.

**SERIOUS ADVERSE EVENT**	Any adverse experience that results in any of the following outcomes:<br>• Death<br>• Is life-threatening<br>• Subject hospitalization or prolongation of existing hospitalization<br>• A persistent or significant disability or incapacity<br>• Congenital anomaly or birth defect<br>• Important medical events that may not be immediately life-threatening or result in death or hospitalization, but may jeopardize the subject or may require intervention to prevent one of the outcomes listed in the definition above.
**SIGNIFICANT EMBOLIC EVENTS**	Unanticipated kidney/bowel infarct clinically driven by symptoms of abdominal or back pain and confirmed with CT scan or open surgery, lower extremity ulceration or gangrene, or kidney failure.
**STENT THROMBOSIS**	Angiographic thrombus or sub-acute closure within the stented vessel.
**SYSTOLIC HEART FAILURE**	Heart failure with ejection fraction < 30% and/or hospitalization requiring intubation and ventilation support for this diagnosis within the previous 90 days.
**TARGET LESION REVASCULARIZATION**	**Clinically-Driven TLR -** TLR (percutaneous balloon angioplasty (PTA), bare metal stent or repeat covered stent deployment) due to documented recurrent hypertension from 30 days post-procedure level and/or deterioration in renal function from baseline value associated with angiographic core laboratory adjudication of a ≥ 60% diameter covered stent restenosis.<br><br>**Incidental TLR** – TLR not meeting the definition of a clinically driven TLR.

**TECHNICAL SUCCESS**	Successful delivery and deployment of the iCAST™ RX Stent System with ≤ 30% residual stenosis after stent deployment (including post-dilatation) assessed via quantitative vascular analysis (QVA) by an independent core laboratory.
**TRANSIENT ISCHEMIC ATTACK**	An attack where a person has stroke-like ("mini-stroke") symptoms for up to 1 - 2 hours; these attacks are often considered a warning sign that a true stroke may happen in the future if something is not done to prevent it.
**TOTAL OCCLUSION**	A complete obstruction or a closure of a passageway or vessel.
**UNANTICIPATED ADVERSE DEVICE EFFECT**	Any serious adverse effect on health or safety, or any life-threatening problem or death caused by, or associated with, a device, if that effect, problem, or death was not previously identified in nature, severity, or degree of incidence in the Investigational Plan or IFU, or any other unanticipated serious problem associated with a device that relates to the rights, safety, or welfare of subjects.
**UNANTICIPATED KIDNEY/BOWEL INFARCT**	Clinically driven by symptoms of abdominal or back pain and confirmed with CT scan or open surgery.

APPENDIX 3: METHODOLOGY FOR OFFICE-BASED BP MEASUREMENT

The following techniques for blood pressure determination have been proposed by the AHA and represent the current gold standard.[37, 53]

Blood pressure must be measured with certified, calibrated, and validated equipment. The size of the bladder within the blood pressure cuff must encircle at least 80% of the arm. Subjects should be seated in a chair with their backs supported and their arms bared and supported at heart level with feet flat on the ground. Subjects should refrain from exercise, smoking or caffeine ingestion for 30 minutes before blood pressure measurement, and measurement should begin after at least 5 minutes of rest (seated comfortably as above). Subjects should also have emptied their bladder prior to obtaining the measurement as a full bladder can affect the reading.

- If necessary, blood pressure may be measured in the supine or reclining position. However, the subject should then be in the same position for subsequent measurements.

- Both systolic and diastolic blood pressure should be recorded, with the first appearance of sound used to define systolic blood pressure and the disappearance of sound used to define diastolic blood pressure.

- A minimum of three (3) readings separated by 2 minutes should be averaged. If the first 3 readings differ by > 5mmHg, additional readings should be obtained and averaged. Blood pressure should be measured in both arms, and the higher value obtained should be used.

- Initial blood pressure should be measured in both arms, to identify which has the higher reading. From that point on, the arm with the higher reading becomes the "BP arm" for the remainder of the trial. For consistency, the site of blood pressure measurement should be recorded, and follow-up pressures should be maintained from the same arm. The appropriately sized blood pressure cuff must again be used, and the site used must be well documented for future examinations.

APPENDIX 4: SCHEDULE OF EVENTS

	(-14)-Days (Medical Documentation Screening period) [7] Prior to Procedure	(-7)-Days[7] Prior to Procedure	Day 0 Procedure	30-Days (+14/-7 Days) Office Visit	6-Months (+/-30 Days) Phone Call	9-Months (+/-30 Days) Office Visit	12-Months (+/- 30 Days) Office Visit	18-Months (+/-30 Days) Phone Call	24-Months (+/- 60 Days) Office Visit	36-Months (+/- 60 Days) Office Visit
**Informed Consent**	X									
**General Entry Criteria**	X									
**Angiographic Entry Criteria**			X							
**Demographics and Medical History**	X									
**Weight (Kg)/ Height (cm)**	X									
**Vital Signs**	X		X	X		X	X		X	X
**BP Assessment[1]**	X[1]	X[1]	X	X		X	X		X	X
**Laboratory Assessments[2]**	X[2]		X[2]	X[2]		X[2]				
**Test for Proteinuria[3]**	X[3]									
**Physical Exam**	X		X	X		X	X		X	X
**Pregnancy Test[4]**	X[4]		X[4]							
**iCAST™ RX Stent System Placement**			X							
**Duplex Ultrasound**				X		X				
**Angiography[5]**			X[5]			X[5]				
**Concomitant Medications**	X	X	X	X	X	X	X	X	X	X
**Adverse Events[6]**	X	X	X	X	X	X	X	X	X	X

[1] A minimum of two office visits are required within the 14-day Medical Documentation Screening period. If the procedure is scheduled > 14 days from the first office visit, subjects should return every two weeks (+/- 3 days) for BP assessments and monitoring.
[2] Chem12 panel which includes: Glucose, Calcium, Albumin, Total protein, Sodium, Potassium, CO2, Chloride, BUN, creatinine, ALP, ALT, AST, and bilirubin.
[3] Test for proteinuria should be completed for subjects during Step 2 of the Medical Documentation Screening Period.
[4] Pregnancy test required at screening and on the day of procedure for females of childbearing potential only.
[5] All subjects must have angiography to determine percent diameter stenosis prior to treatment and at 9-months (if DUS indicates ≥ 60% stenosis).
[6] AE collection (serious and non-serious) will begin upon completion of the ICF process through the 9-month follow-up visit, thereafter only SAEs and UADEs will be recorded.
[7] Subjects with a history of > 1 month stable medication per protocol and documented SBP ≥ 155mmHg will be allowed to skip the formal Screening period. All other protocol requirements that would have normally occurred during the two week screening period must still be completed prior to day of procedure.

APPENDIX 5: REFERENCES

1. Carr TM, Sabri SS, Turba UC, et al .Stenting for atherosclerotic renal artery stenosis. Tech Vasc Interv Radiol. 2010 Jun;13(2):134-45.
2. Rocha-Singh K, Jaff MR, Lynne Kelley E. Renal artery stenting with noninvasive duplex ultrasound follow-up: 3-year results from the RENAISSANCE renal stent trial. Catheter Cardiovasc Interv 2008;72:853–862.
3. Van Jaarsveld BC, Krijnen P, PietermanH, Derkx FH, Deinum J, et al. the effect of balloon angioplasty on hypertension in atherosclerotic renal artery stenosis, Dutch Renal Artery Stenosis Intervention Cooperative Trial Group, N Engl J Med 2000; 342:1007-1014
4. Hoffman O, Carreres T, Sapoval MR, Auguste MC, Beyssen BM, Raynaud AC, Gaux JC. Ostial renal artery stenosis angioplasty: Immediate and mid-term angiographic and clinical results. J Vasc Interv Radiol 1998;9(Part 1):65-73.
5. Hirsch A, Haskal Z, Hertzer N, et al. ACC/AHA 2005 practice guidelines for the management of patients with peripheral arterial disease (lower extremity, renal mesenteric and abdominal aortic): a collaborative report from the American Association for Vascular Surgery/Society for Vascular Surgery, Society for Cardiovascular Angiography and Intervention, Society for Vascular Medicine and Biology, Society of Interventional Radiology, and the ACC/AHA Task Force on Practice Guidelines (Writing Committee to Develop Guidelines for the Management of Patients with Peripheral Arterial Disease). Circulation 2006; 113:e463-e654.
6. Weibull H, Bergqvist D, Jonsson K, Hulthen L, Mannheim P, Bergentz SE. long term results after percutaneous transluminal angioplasty of the atherosclerotic renal artery stenosis – the importance of intensive follow-up. Eur J Vasc Surg 1991;5:291-301.
7. Symonides B, Januszewicz A, Rowinski O, Januszewicz M, Chodakowska J, Berent H, Kuczynska K, Szmigielski C, Malek G, Januszewicz W. plasma fibrinogen as a risk factor for restenosis after percutaneous transluminal renal angioplasty in patients with atherosclerotic renal artery stenosis. J Cardiovasc Risk 1999;6:269-272.
8. Van de Ven PJ, Kaatee R, Beutler JJ, Beek FJ, Woittiez AJ, Buskens E, Koomans HA, Mali WP. Arterial stenting and balloon angioplasty in ostial atherosclerotic renovascular disease: A randomized trial. Lancet 1999;353:282-286.
9. Rocha-Singh K, Jaff M, Rosenfield K, et al. Evaluation of the safety and effectiveness of renal artery stenting after unsuccessful balloon angioplasty: The results of the ASPIRE-2 study. J Am Coll Cardiol 2005; 46:776-783.
10. Cooper C, Murphy T, Matsumoto A, et al. Stent revascularization for the prevention of cardiovascular and renal events among patients with renal artery stenosis and systolic hypertension: rationale and design of the CORAL trial. Am Heart J 2006; 152:59-66.
11. Weibull H, Bergqvist D, Bergentz SE, Jonsson K, Hullthen L, Manhem P. percutaneous transluminal renal angioplasty versus surgical reconstruction of atherosclerotic renal artery stenosis: A prospective randomized trial. J Vasc Surg 1993;18:841-850;discussion 850-852.
12. Plouin PF, Chatellier G, Darne B, et al. Blood pressure outcome of angioplasty in artherosclerotic renal artery stenosis : A randomised trial. The EMMA-Trial Group. Hypertension 1998;31:823-829.
13. Webster J, Marshall F, Abdalla M, et al. Randomised comparison of percutaneous angioplasty vs continued medical therapy for hypertensive patients with atheromatous renal artery stenosis. J Hum Hypertens 1998;12:329-335.
14. The ASTRAL Investigators. Revascularization versus Medical Therapy for renal artery stenosis. The ASTRAL investigators. N Engl J Med 2009;361:1953-62.
15. Sharafuddin M, Raboi C, Abu-Yousef M, et al. renal artery stenosis: Duplex US after angioplasty and stent placement. Radiology 2001;220:168-173.
16. Lederman RJ, Mendelsohn FO, Santos R, et al. Primary renal artery stenting: characteristics and outcomes after 363 procedures. Am Heart J. 2001;142:314–323.

17. Corriere MA, Pearce JD, Edwards MS, et al. Endovascular management of atherosclerotic renovascular disease: Early results following primary intervention. J Vasc Surg 2008;48:580–587.
18. Stone GW, Goldberg S, Mehran R, et al. A prospective, randomized U.S. trial of the PTFE-covered Jostent for the treatment of diseased saphenous vein grafts: The BARRICADE trial (Abstr). J Am Coll Cardiol 2005;45:27A.
19. Bates MC, Shamsham FM, Faulknier B, et al. Artery perforation during catheterization: Fighting with a catastrophe. Catheter Cardiovasc Interv 2002;57:44–46.
20. Allaire E, Melliere D, Poussier B, et al. Iliac artery rupture during balloon dilatation: What treatment? Ann Vasc Surgery 2003;17:306–314. Epub 2003 Apr 28.
21. Beek FJ, Kaatee R, Beutler JJ, et al. Complications during renal artery stent placement for atherosclerotic ostial stenosis. Cardiovasc Interv Radiol 1997;20:184–190.
22. Kohler Tr, Zierler RE, Martin RL, Nicholls SC, Gergelin RO, Kazmers A, Beach KW, Strandness DE Jr. Noninvasive diagnosis of renal artery stenosis by ultrasonic duplex scanning. J Vasc Surg 1986;4:450-456.
23. Patel PM, Eisenberg J, Islam MA, et al. Percutaneous revascularization of persistent renal artery in-stent restenosis. Vasc Med 2009; 14: 259.
24. Anchala PR. Resnick SA. The current state of endovascular therapy in the evaluation and management of renovascular disease. Semin Intervent Radiol. 2009 Dec;26(4):333-44.
25. Lakshminarayan Y, De A, Jolly N. Covered Stents in Renal Artery Interventions. Cath Lab Digest Archive October, 2010.
26. Leertouwer TC, Gussenhoven EJ, Bosch JL et al. Stent Placement for Renal Arterial Stenosis: Where Do We Stand? A Meta-analysis. Radiology 2000; 216:78–85.
27. Miller R. HERCULES: Renal Stenting lowers BP, but BNP is not a marker of benefit. Interventional Surgery. 2011.
28. Taylor DC, Kettler MD, Monetta GL, Kohler TR, Kazmers A, Beach KW, Strandness De Jr. Duplex ultrasound scanning in the diagnosis of renal artery stenosis: A prospective evaluation. J Vasc Surg 1988;7:363-369.
29. Olin JW, Piedmonte MR, Young JR, DeAnna S, Grubb M, Childs MB. The utility of duplex ultrasound scanning of the renal arteries for diagnosing significant renal artery stenosis. Ann Intern Med 1995;122:833-838.
30. Zierler RE. Is duplex scanning the best screening test for renal artery stenosis? Semin Vasc Surg 2001;14:177-185.
31. Carman TL, Olin JW, Czum J. Noninvasive imaging of the renal arteries. Urol Clin North Am 2001;28:815-826.
32. Bakker J, Beutler J, Elgersma O, et al. Duplex ultrasonography in assessing restenosis of renal artery stents. Cardiovasc Intervent Radiol 1999;22:475-480.
33. The Joint National Committee on Prevention, Detection, Evaluation, and Treatment of High Blood Pressure. The seventh report of the Joint National Committee on Prevention, Detection, Evaluation, and Treatment of High Blood Pressure. 2004.
34. Rabbia, C, Pini R. Evidence-based medicine in renal artery stenting. Journal of Cardiovascular Surgery 2010; 51: 755-63.
35. Balachandran KP, Oldroyd KG. Successful reintroduction of previously failed ACE inhibitor therapy following stenting of atherosclerotic renovascular disease. Indian Heart J. 2008 Jan-Feb;60(1):55-7.
36. Rocha-Singh KJ, Novack V, Pencina M et al. Objective performance goals of safety and blood pressure efficacy for clinical trials of renal artery bare metal stents in hypertensive patients with atherosclerotic renal artery stenosis. Catheter Cardiovasc Interv. 2011.
37. Rundback JH, Sacks D, Kent KC, et al. Guidelines for the reporting of renal artery revascularization in clinical trials. American Heart Association. Circulation. 2002;106:1572–1585.

38. Kandarpa K J, Becker GJ, Hunink MGM, et al. Transcatheter Interventions for the Treatment of Peripheral Atherosclerotic Lesions. Vasc Interv Radiology 2001 (12)6: 683-695.
39. Laird JR, Rundback J, Zierler E, et al. Safety and Efficacy of Renal Artery Stenting Following Suboptimal Renal Angioplasty for De Novo and Restenotic Ostial Lesions: Results from a Nonrandomized, Prospective Multicenter Registry. J Vasc Interv Radiol 2010; 21:627–637.
40. Fleming Sh, Davis RP, Craven TE et al. Accuracy of duplex sonography scans after artery stenting. J Vasc Surg 2010; 52: 953-8.
41. Corriere MA, Edwards MS, Pearce JD, et al. Restenosis after renal artery angioplasty and stenting: Incidence and risk factors J Vasc Surg. 2009 October; 50(4): 813–819.
42. Nolan BW, Schermerhorn ML, Powell RJ, et al. Restenosis in gold-coated renal artery stents. J Vasc Surg 2005;42:40-6.
43. Nolan BW, Schermerhorn ML, Rowell E, et al. Outcomes of renal artery angioplasty and stenting using low-profile systems. J Vasc Surg 2005;41:46-52.
44. Sapoval M, Zähringer M, Pattynama P, Rabbia C, et al. Low-profile stent system for treatment of atherosclerotic renal artery stenosis: the GREAT trial. J Vasc Interv Radiol. 2005 Sep; 16(9):1195-202.
45. Shammas NW, Kapalis MJ, Dippel EJ, et al. Clinical and angiographic predictors of restenosis following renal artery stenting. J Invasive Cardiol. 2004;16:10–13.
46. Rundback JH, Gray RJ, Rozenblit G, et al. Renal artery stent placement for the management of ischemic nephropathy. J Vasc Interv Radiol 1998; 9:413–420.
47. Tullis MJ, Zierler RE, Glickerman DJ, Bergelin RO, Cantwell-Gab K, Strandness DE Jr. Results of percutaneous transluminal angioplasty for atherosclerotic renal artery stenosis: a follow-up study with duplex ultrasonography. J Vasc Surg 1997; 25:46–54.
48. Harden PN, MacLeod MJ, Rodger RSC, et al. Effect of renal artery stenting on progression of renovascular renal failure. Lancet 1997; 349:1133–1136.
49. White CJ, Ramee SR, Collins TJ, Jenkins JS, Escobar A, Shaw D. Renal artery stent placement: utility in lesions difficult to treat with balloon angioplasty. J Am Coll Cardiol 1997; 30:1445–1450.
50. Iannone LA, Underwood PL, Nath A, Tannenbaum MA, Ghali MGH, Clevenger LD. Effect of primary balloon expandable renal artery stents on long-term patency, renal function, and blood pressure in hypertensive and renal insufficient patients with renal artery stenosis. Cathet Cardiovasc Diagn 1996; 37:243–250.
51. Dorros G, Jaff M, Mathiak L, et al. Four year follow-up of Palmaz-Schatz stent revascularization as treatment for atherosclerotic renal artery stenosis. Circulation1998; 98:642–647.
52. Hennequin LM, Joffre FG, Rousseau HP, et al. Renal artery stent placement: long-term results with the Wallstent endoprosthesis. Radiology 1994; 191:713–719.
53. Pickering TG, Hall JE, Appel LJ, et al. Recommendations for Blood Pressure Measurement in Humans and Experimental Animals: Part 1: Blood Pressure Measurement in Humans: A Statement for Professionals From the Subcommittee of Professional and Public Education of the American Heart Association Council on High Blood Pressure Research. Circulation 2005; 111: 697-716.
