# Supplementary material for: De Novo Atherosclerotic Renal Artery Stenosis Covered Stent Treatment for Resistant Hypertension (ARTISAN) Results
Source: J Soc Cardiovasc Angiogr Interv. 2024 Oct 18;3(12):102400. doi: 10.1016/j.jscai.2024.102400 (PMC11725122; doi:10.1016/j.jscai.2024.102400)
Supplement: Supplemental Material 3 [file mmc3.docx]

ARTISAN Supplement 2:

DERIVATION OF THE PERFORMANCE GOAL FOR PATENCY

The Performance Goal was derived from a thorough literature review of trials with renal bare metal stent placement. Trials were identified by means of a PUBMED search of the English-language medical literature from 1991 to 2010. When available, the following data were extracted: author and year of publication, number of subjects, number of arteries, restenosis definition, evaluation method, duration of follow-up, and restenosis rate**.** Of the 30 articles reviewed, 9 articles were excluded from the Performance Goal based on the following: results from studies with data collection from the 1980’s which is not representative of today’s standard of care, definitions used for restenosis were outside of our trial design, follow-up occurred at times outside of our assessments, and results from studies not yet published.

The following references were deemed relevant for the patency Performance Goal:

Table: Derivation of Patency Performance Goal

| **Author** | **Year** | **Number of Subjects** | **Number of Arteries** | **Evaluation Method** | **Follow-up Time** | **Restenosis**  **Rate** |
| --- | --- | --- | --- | --- | --- | --- |

| Rocha -Singh et al^1^ | 2005 | 208 | 252 | DUS | 9 mo | 17.4% |
| --- | --- | --- | --- | --- | --- | --- |
| Laird et al^2^ | 2010 | 188 | 188 | DUS | 9-12 mo | 16.8% |
| Fleming et al^3^ | 2010 | 30 | 66 | Angio | 6 mo | 35% |
| Corriere et al^4^ | 2009 | 91 | 101 | DUS | 12 mo | 26.7% |
| Rocha-Singh et al^5^ | 2008 | 100 | 117 | DUS | 9 mo | 21.3% |
| Nolan et al^6^ | 2005 | 78 | 97 | DUS | 12 mo | 25% |
| Nolan et al^7^ | 2005 | 82 | 96 | DUS | 12 mo | 25% |
| Sapoval et al^8^ | 2005 | 52 | 52 | Angio | 6 mo | 14% |
| Shammas et al^8^ | 2004 | 58 | 58 | DUS, Angio, CTa | 2-20 mo | 26% |
| Lederman et al^10^ | 2001 | 300 | 358 | Angio | 16 mo | 21% |
| Symonides et al^11^ | 1999 | 27 | 27 | DUS, Angio to confirm | 6 mo | 30% |
| Van de Ven et al^12^ | 1999 | 42 | 51 | Angio | 6 mo | 14% |
| Rundback et al^13^ | 1998 | 45 | 32 | Angio | 12 mo | 25% |
| Tullis, et al^14^ | 1997 | 41 | 52 | Angio/DUS | 12 mo | 44% |
| Harden et al^15^ | 1997 | 32 | 33 | Angio | 6 mo | 12% |
| White et al^16^ | 1997 | 100 | 133 | Angio | 6-12 mo | 19% |
| Iannone et al^17^ | 1996 | 63 | 86 | DUS | 12 mo | 14% |
| Dorros et al^18^ | 1995 | 76 | 92 | Angio | 6 mo | 25% |
| Hennequin et al^19^ | 1994 | 21 | 25 | Angio | 12 mo | 19% |

The weighted mean restenosis rate from these trials is 21.4%. Assuming a restenosis rate for PTRA of 40% as noted in Carr based on the analysis of 6 trials with PTRA^(20)^ and also used by Rocha-Singh,^(5)^ a restenosis rate of 30% leading to a Performance Goal for primary patency set at 70%. This goal will preserve at least 50% of the difference between the currently available bare metal stent rate and the PTRA rate, as well as adjust for a lesion set with a higher percentage stenosis (80%-100%) than previous reported studies (50%-100%).

1. Rocha-Singh K, Jaff M, Rosenfield K, et al. Evaluation of the safety and effectiveness of renal artery stenting after unsuccessful balloon angioplasty: The results of the ASPIRE-2 study. J Am Coll Cardiol 2005; 46:776-783.
2. Laird JR, Rundback J, Zierler E, et al. Safety and Efficacy of Renal Artery Stenting Following Suboptimal Renal Angioplasty for De Novo and Restenotic Ostial Lesions: Results from a Nonrandomized, Prospective Multicenter Registry. J Vasc Interv Radiol 2010; 21:627–637.
3. Fleming Sh, Davis RP, Craven TE et al. Accuracy of duplex sonography scans after artery stenting. J Vasc Surg 2010; 52: 953-8.
4. Corriere MA, Edwards MS, Pearce JD, et al. Restenosis after renal artery angioplasty and stenting: Incidence and risk factors [J Vasc Surg. 2009 October; 50(4): 813–819.](http://www.ncbi.nlm.nih.gov/entrez/eutils/elink.fcgi?dbfrom=pubmed&retmode=ref&cmd=prlinks&id=19595532)
5. Rocha-Singh K, Jaff MR, Lynne Kelley E. Renal artery stenting with noninvasive duplex ultrasound follow-up: 3-year results from the RENAISSANCE renal stent trial. Catheter Cardiovasc Interv 2008;72:853–862.
6. Nolan BW, Schermerhorn ML, Powell RJ, et al. Restenosis in gold-coated renal artery stents. J Vasc Surg 2005;42:40-6.
7. Nolan BW, Schermerhorn ML, Rowell E, et al. Outcomes of renal artery angioplasty and stenting using low-profile systems. J Vasc Surg 2005;41:46-52.
8. Sapoval M, Zähringer M, Pattynama P, Rabbia C, et al. Low-profile stent system for treatment of atherosclerotic renal artery stenosis: the GREAT trial. J Vasc Interv Radiol. 2005 Sep; 16(9):1195-202.
9. Shammas NW, Kapalis MJ, Dippel EJ, et al. Clinical and angiographic predictors of restenosis following renal artery stenting. J Invasive Cardiol. 2004;16:10–13.
10. Lederman RJ, Mendelsohn FO, Santos R, et al. Primary renal artery stenting: characteristics and outcomes after 363 procedures. Am Heart J. 2001;142:314–323.
11. Symonides B, Januszewicz A, Rowinski O, Januszewicz M, Chodakowska J, Berent H, Kuczynska K, Szmigielski C, Malek G, Januszewicz W. plasma fibrinogen as a risk factor for restenosis after percutaneous transluminal renal angioplasty in patients with atherosclerotic renal artery stenosis. J Cardiovasc Risk 1999;6:269-272.
12. Van de Ven PJ, Kaatee R, Beutler JJ, Beek FJ, Woittiez AJ, Buskens E, Koomans HA, Mali WP. Arterial stenting and balloon angioplasty in ostial atherosclerotic renovascular disease: A randomized trial. Lancet 1999;353:282-286.
13. Rundback JH, Gray RJ, Rozenblit G, et al. Renal artery stent placement for the management of ischemic nephropathy. J Vasc Interv Radiol 1998; 9:413–420.
14. Tullis MJ, Zierler RE, Glickerman DJ, Bergelin RO, Cantwell-Gab K, Strandness DE Jr. Results of percutaneous transluminal angioplasty for atherosclerotic renal artery stenosis: a follow-up study with duplex ultrasonography. J Vasc Surg 1997; 25:46–54.
15. Harden PN, MacLeod MJ, Rodger RSC, et al. Effect of renal artery stenting on progression of renovascular renal failure. Lancet 1997; 349:1133–1136.
16. White CJ, Ramee SR, Collins TJ, Jenkins JS, Escobar A, Shaw D. Renal artery stent placement: utility in lesions difficult to treat with balloon angioplasty. J Am Coll Cardiol 1997; 30:1445–1450.
17. Iannone LA, Underwood PL, Nath A, Tannenbaum MA, Ghali MGH, Clevenger LD. Effect of primary balloon expandable renal artery stents on long-term patency, renal function, and blood pressure in hypertensive and renal insufficient patients with renal artery stenosis. Cathet Cardiovasc Diagn 1996; 37:243–250.
18. Dorros G, Jaff M, Mathiak L, et al. Four year follow-up of Palmaz-Schatz stent revascularization as treatment for atherosclerotic renal artery stenosis. Circulation1998; 98:642–647.
19. Hennequin LM, Joffre FG, Rousseau HP, et al. Renal artery stent placement: long-term results with the Wallstent endoprosthesis. Radiology 1994; 191:713–719.
20. Carr TM, Sabri SS, Turba UC, et al .Stenting for atherosclerotic renal artery stenosis. Tech Vasc Interv Radiol. 2010 Jun;13(2):134-45.
