# Supplementary material for: De Novo Atherosclerotic Renal Artery Stenosis Covered Stent Treatment for Resistant Hypertension (ARTISAN) Results
Source: J Soc Cardiovasc Angiogr Interv. 2024 Oct 18;3(12):102400. doi: 10.1016/j.jscai.2024.102400 (PMC11725122; doi:10.1016/j.jscai.2024.102400)
Supplement: Supplemental Material 4 [file mmc4.pdf]

Protocol/CIP No: iCAST™ RX-ARAS-001

ARTISAN
iCAST™ RX De Novo Stent Placement for the Treatment of Atherosclerotic Renal Artery Stenosis in Subjects with Resitant Hypertension

Statistical Analysis Plan

Prepared for:
Atrium MAQUET GETINGE Group
40 Continental Blvd.
Merrimack, NH, 03054
U.S.A

Version v1.0
Date: 12Feb2018

Prepared by: [redacted]

VERSION HISTORY OF IMPLEMENTED PLANS

Version	Date	Revision Author	Comments
V0.1	10May2016	[redacted]	The main focus of this version is towards safety. Efficacy endpoints were not reviewed.
V0.1	18Jun2016	[redacted]	Safety endpoints revision
V1.0	12Feb2018	[redacted]	Updated efficacy analysis sections to reflect the changes from the planned analysis after termination of study enrollment

Table of Contents

1. LIST OF ABBREVIATIONS AND DEFINITIONS OF TERMS 4

2. DEVIATIONS FROM THE PROTOCOL 6

3. INTRODUCTION 6

4. STUDY OBJECTIVES 7

5. STUDY DESIGN 9

5.1 GENERAL DESIGN 9
5.2 DISCUSSION OF ENDPOINT DETERMINATION 9
5.3 METHOD OF ASSIGNMENT OF SUBJECTS TO TREATMENT GROUPS 10
5.4 BLINDING 10
5.5 DETERMINATION OF SAMPLE SIZE 10
5.6 DERIVATION OF THE PERFORMANCE GOAL FOR PATENCY 11
5.7 STATISTICAL HYPOTHESIS 12

6. CHANGES IN THE CONDUCT OF THE STUDY OR PLANNED ANALYSES 13

6.1 CHANGES IN THE CONDUCT OF THE STUDY 13
6.2 CHANGES FROM THE ANALYSES PLANNED IN THE PROTOCOL/CIP 13

7. BASELINE, EFFICACY AND SAFETY EVALUATIONS 14

7.1 SCHEDULE OF EVALUATION 14
7.2 TIME POINT ALGORITHMS 16
7.3 HANDLING OF MISSING DATE/TIME DATA 16
7.3.1. Missing Date 16
7.3.2. Missing Time 16
7.4 VISIT WINDOWS 17
7.5 BASELINE ASSESSMENTS 17
7.6 ENDPOINTS, SAFETY OUTCOMES AND OTHER PARAMETERS 18
7.6.1. Primary Endpoints 18
7.6.2. Secondary Endpoints 19
7.6.3. Safety Outcomes and Other Parameters 23

8. STATISTICAL METHODS 23

8.1 GENERAL STATISTICAL CONSIDERATIONS 23
8.2 ADJUSTMENTS FOR COVARIATES 24
8.3 HANDLING OF DROPOUTS OR MISSING DATA 24
8.4 INTERIM ANALYSES AND DATA MONITORING 24
8.5 CLINICAL EVENTS COMMITTEE (CEC) 25
8.6 MULTI-CENTER STUDIES AND POOLING OF CENTERS 25
8.7 MULTIPLE COMPARISONS/MULTIPLICITY 26
8.8 UNIT OF ANALYSIS 26

9. STATISTICAL ANALYSIS 26
9.1 DISPOSITION OF SUBJECTS 26
9.2 PROTOCOL DEVIATIONS 27
9.3 ANALYSIS POPULATION 27
9.4 DEMOGRAPHIC AND OTHER BASELINE CHARACTERISTICS 27
9.5 TARGETED MEDICAL HISTORY 28
9.6 ANTI-HYPERTENSIVE MEDICAL HISTORY 30
9.7 PROCEDURAL CHARACTERISTICS 30
9.8 PRIOR AND CONCOMITANT THERAPY 31
9.9 ANALYSIS OF PRIMARY ENDPOINTS 32
9.9.1. Primary Patency Analysis 33
9.9.2. Systolic Blood Pressure Analysis 33
9.9.3. Sensitivity Analysis 33
9.9.4. Subgroup Analyses 34
9.9.5. Exploratory Analyses 34
9.10 ANALYSIS OF SECONDARY ENDPOINTS 34
9.10.1. TLR 34
9.10.2. Changes in the number or dosage of antihypertensive medications 34
9.10.3. Other secondary endpoints 35
1. Occurrence of Major Adverse Events (MAEs) 35
2. Acute procedural success 35
3. Incidental Target lesion revascularization (TLR) 35
4. Rate of incidental TLR 35
5. Improved SBP control assessed at 30-days, 9-months, 12-months, 24-months and 36-months 35
6. Secondary patency rate at 9-months 35
7. Change in number and dosage of anti-hypertensive medications 35
8. Renal function compared to baseline as measured by eGFR at 30-days and 9-months 35
9.11 ANALYSIS OF SAFETY AND OTHER PARAMETERS 35
9.11.1. Adverse Events 35
9.11.2. Serious Adverse Events (SAE) 36
9.11.3. Unanticipated Adverse Device Effect (UADE) 37
9.11.4. Anticipated Adverse Events 37
9.11.5. Device Failures, Malfunctions and Near Incidents 39
9.11.6. Clinical Laboratory Evaluations 39
9.11.7. Vital Signs 40
9.11.8. Duplex Ultrasound (DUS) 40
9.11.9. Physical Examination 41
9.11.10. Angiographic Results and Revascularization 41
9.11.11. Revascularization 42
9.11.12. Extent of Device Exposure 44
9.11.12.1. Extent of Exposure 44
9.11.13. Mortality 44

10. COMPUTER SOFTWARE 44

11. TABLE SHELLS AND SPECIFICATIONS 44

1. LIST OF ABBREVIATIONS AND DEFINITIONS OF TERMS

Table 1: Abbreviations and Definitions of Terms

ACE	Angiotensin Converting Enzyme
AE(s)	Adverse event(s)
ACC	American College of Cardiology
AHA	American Heart Association
ARAS	Atherosclerotic Renal Artery Stenosis
ARB	Angiotensin Receptor Blocking Agent
CEC	Clinical Events Committee
ATC	Anatomical therapeutic chemical
BMI	Body mass index
BP	Blood pressure
CABG	Previous Coronary Artery Bypass Surgery
CAD	Coronary Artery Disease
CHD	Coronary Heart Disease
CHF	Coronary Heart Failure
CI	Confidence interval
CT	Contrast Tomography
DBP	Diastolic Blood Pressure
DMP	Data Management Plan
DSMB	Data Safety Monitoring Committee
DUS	Duplex Ultrasound
eCRF	Electronic Case report form
eGFR	Estimated glomerular filtration rate
FDA	Food and Drug Administration
FFR	Fraction Flow Reserve
HEENT	Head, eyes, ears, nose, and throat
HR	Heart rate
hr(s)	Hour(s)
ICF	Informed Consent Form
IFU	Instructions for Use
ITT	Intent-to-treat
KM	Kaplan-Meier
MAE	Major Adverse Event
MI	Myocardial Infarction
max	Maximum
MedDRA	Medical dictionary for regulatory activities
MDRD	Modification of Diet in Renal Disease
Mg	Milligram
min	Minimum
MLD	Minimal lumen diameter
N	Number of subjects
NYHA	New York Heart Association

OUS	Outside-of the US
PP	Per Protocol
PT	Preferred Term
PTA	Percutaneous Balloon Angioplasty
PTRA	Percutaneous transluminal renal angioplasty
QVA	Quantitative Vascular Analysis
RAS	Renal Artery Stenosis
RR	Respiration rate
RVD	Reference vessel diameter
SAP	Statistical Analysis Plan
SAE	Serious adverse event
SBP	Systolic blood pressure
SD	Standard deviation
SOC	System organ class
SOP	Standard Operating Procedure
TIA	Transient Ischemic Attack
TIMI	Thrombus in Myocardian Infarction
TLR	Target Lesion Revascularization
UADE	Unanticipated Adverse Device Effect
US	United States
WHO	World Health Organization

2. DEVIATIONS FROM THE PROTOCOL

Due to the early termination of enrollment for the Protocol/CIP No. iCAST™ RX-ARAS-001 in November 2017, all inferential analysis should be interpreted as descriptive and will be carried out in an exploratory manner.

Missing data will not be imputed for analysis purposes as there is no need to evaluate the robustness of the primary analysis or the impact of missing data.

3. INTRODUCTION

This Statistical Analysis Plan (SAP) describes the planned analysis and reporting for protocol iCAST™ RX-ARAS-001, entitled, "iCAST™ RX De Novo Stent Placement for the Treatment of Atherosclerotic Renal Artery Stenosis in Patients with Resistant Hypertension", amendment #1 dated July 15th, 2013. The purpose of this SAP is to describe the statistical methodologies that will be used to address the objectives of the above study and come to conclusions regarding the study aims, ensuring their validity and suitability.

Atherosclerotic renal artery stenosis (ARAS) is the most common cause of renal artery stenosis (RAS) in the adult population. (1) ARAS may result in progressive renal impairment, renovascular hypertension, and/or cardiac disturbance syndromes. As the disease progresses, the resulting luminal narrowing decreases blood flow to the kidney, which, in turn, is often accompanied by a reduction in kidney function as well as hypertension triggered by activation of the renin-angiotensin axis. If left untreated, ARAS may progress to renal failure. (2)

The identification and management of subjects with suboptimal control of hypertension and coexistent atherosclerotic renal artery stenosis is both challenging and controversial.(3, 4) Current ACC/AHA Peripheral Arterial Disease Guidelines identify renal artery stent deployment as a revascularization strategy in subjects with hypertension resistant to optimal medical management.(5) However, a contemporary literature review suggested that there is no data supporting a revascularization strategy over optimal antihypertensive medical therapy.(6) Indeed, several single-arm prospective trials sponsored by commercial device manufacturers, while establishing the

procedural safety of renal artery stent deployment after failed/suboptimal balloon angioplasty, were unable to demonstrate a predictable and durable reduction in blood pressure.(7, 8)

More than 50% of subjects with uncontrolled hypertension unresponsive to traditional medical therapy may have significant ARAS and are considered candidates for surgical revascularization or percutaneous intervention for the resolution or stabilization of hypertension and renal dysfunction. Surgical revascularization has proven to be an effective treatment with acceptable long-term patency rates but is hampered by significant morbidity and mortality. (2) Percutaneous transluminal renal angioplasty (PTRA) has been successful in reducing morbidity and mortality compared to surgical revascularization but has poor technical success due to high rates of elastic recoil and dissection for typical ostial lesions, as well as high restenosis rates. Primary stenting of atherosclerotic ostial lesions in the renal artery has thus become the preferred method of treatment due to higher success rates compared to surgical revascularization.(2)

4. STUDY OBJECTIVES

The primary objective of this study is to assess the safety and efficacy of the iCAST™ RX Stent System in subjects with resistant hypertension associated with de novo atherosclerotic renal artery disease. The primary objective will be evaluated using co-primary endpoints. The primary functional endpoint will assess safety and effectiveness based on the primary patency rate at 9-months on a per lesion basis evaluated against a performance goal of published studies with bare-metal stents. The primary clinical endpoint will assess the improvement in Systolic Blood Pressure (SBP) at 9-months as compared to baseline SBP.

The secondary objectives of this study are:

1) To evaluate the occurrence of procedure-related Major Adverse Events (MAEs) reported as percentage of subjects with MAE, at 30-days, 9-months, 12-months, 24-months, and 36-months. Inclusive of:
    i) Procedure-or device-related occurrence of death
    ii) Q-Wave myocardial infarction (MI)
    iii) Clinically driven target lesion revascularization (TLR)

(1) Defined as a TLR (Percutaneous Balloon Angioplasty (PTA), bare metal stent or repeat covered stent deployment, or surgical bypass) due to documented recurrent hypertension from 30-days post-procedure level and/or deterioration in renal function from baseline value, associated with angiographic core laboratory adjudication of a ≥ 60% diameter covered stent restenosis.

iv) Significant embolic events

(1) Defined as unanticipated kidney/bowel infarct clinically driven by symptoms of abdominal or back pain and confirmed with Computed Tomography (CT) scan or open surgery, lower extremity ulceration or gangrene, or kidney failure after 30-days, 9-months, 12-months, 24-months, and 36-months.

MAEs were reviewed and adjudicated by the Clinical Event Committee. The adjudicated data will be used to perform the analysis of this endpoint.

2) To determine technical success defined as successful delivery and deployment of the iCAST™ RX Stent System with ≤ 30% residual angiographic stenosis after covered stent deployment (including post-dilatation) assessed via Qualitative Vascular Analysis (QVA) by an independent core laboratory.
3) To determine acute procedural success defined as technical success without the occurrence of MAE prior to hospital discharge.
4) To evaluate TLR measured as the proportion of subjects that require a clinically-driven re-intervention of the target lesion through 9-months.
    i) A clinically-driven TLR is defined as a TLR (PTA, bare metal stent or repeat covered stent deployment, or surgical bypass) due to documented recurrent hypertension from 30-days post-procedure level and/or deterioration in renal function from baseline value, associated with angiographic core laboratory adjudication of a ≥ 60% diameter covered stent restenosis.

Clinically driven TLR were reviewed and adjudicated by the Clinical Event Committee.

5) To determine the rate of incidental TLR defined as rate of TLRs not meeting the definition of a clinically driven TLR.

6) To measure improvement in SBP control assessed at 30-days, 9-months, 12-months, 24-months and 36-months.
7) To determine the secondary patency rate at 9-months after a clinically-driven TLR; which restores patency after total occlusion.
8) To measure the change in number and dosage of anti-hypertensive medications as compared to baseline.
9) To evaluate renal function compared to baseline as measured by estimated Glomerular Filtration Rate (eGFR) at 30-days and 9-months.

5. STUDY DESIGN

5.1 General Design

This is a prospective, single-arm, multicenter clinical trial that will take place at up to 25 US/OUS sites. Primary endpoints have been determined to show the safety, effectiveness, and clinical outcomes of the iCAST™ RX Stent System.

After meeting screening and clinical eligibility criteria, subjects will undergo a baseline assessment for angiographic eligibility. After angiographic documentation of a ≥ 80% diameter renal artery stenosis or Fraction Flow Reserve (FFR) < 0.8 is confirmed, the subject may be enrolled in the trial by placement of the investigational device.

Primary endpoint data is obtained at the 9-month visit and will include a follow-up Duplex Ultrasound (DUS) of the target renal artery(s). If the DUS is indicative of ≥ 60% stenosis as determined by the core laboratory, or the DUS measures are non-diagnostic, a contrast enhanced angiogram will be used to assess the degree of restenosis of the covered stent(s).

Follow-up will be required for all treated subjects as detailed in Section 6.

5.2 Discussion of Endpoint Determination

The primary functional endpoint of primary patency has been established since the clinical care

of the physician is considered a more objective measurement of stent performance than the restenosis measurement of DUS alone. In standard practice, a DUS is used to determine patency of a stent. If a DUS is negative and the subject is asymptomatic, no intervention is typically conducted by a physician. If a subject is symptomatic or a DUS is positive, a confirmatory angiogram is typically performed to assess the patency of the vessel.

To properly match the functional outcomes with historical data, the endpoint will be calculated on a per lesion basis. This reflects the current standard of care of treating subjects with single vessel renal artery lesions and those subjects with bilateral renal artery stenosis. Analysis of past studies has demonstrated that the number of lesions treated per subject has increased with time. A recent renal trial reported 1.2 lesions per subject were treated.

The primary clinical endpoint of reduction in SBP has been established to determine the benefit of revascularization therapy to perfuse the kidney. The Framingham Heart Study showed that decreasing SBP by 10mmHg can be lifesaving. Additionally, Joint National Committee VII (JNC VII) estimates that a 5mmHg reduction in SBP would reduce the incidence of stroke in subjects by 14%, coronary heart disease (CHD) by 9%, and overall mortality by 7%. JNC VII also presented data from a UK diabetes study that demonstrated a 10mmHg decrease in SBP was associated with average reductions of 15% for diabetes-related mortality, 11% for MI, and 13% for microvascular complications of retinopathy or nephropathy.

5.3 Method of Assignment of Subjects to Treatment Groups

This is a single-arm study therefore subjects were not randomized to treatment.

5.4 Blinding

This is an open label study therefore treatment blinding was not implemented.

5.5 Determination of Sample Size

For a one-sided alpha of 0.025 and desired power of 87%, sample size for the primary patency endpoint is established by assuming a hypothesized primary patency incidence of 0.82, which

gives 125 evaluable subjects therefore, accounting for 10% attrition, 138 subjects are needed for this endpoint. At the proposed evaluable sample size of 125, no more than 27 subjects (22%) can fail primary patency in order to meet the endpoint hypothesis.

For the endpoint of reduction in SBP, we assume an average reduction of 13.7mmHg. Under a hypothesized standard deviation of 12mmHg for a difference in SBP there will be 92% power to meet the Performance Goal. This standard deviation is conservatively approximated based on the expected range of SBP at baseline of 155mmHg to 180mmHg divided by 3 and assuming a similar standard deviation at the 9-month follow up. A mean difference in SBP at 9-months of 12mmHg will be required in order to reject the null hypothesis at a one-sided alpha of 0.025 at the proposed sample size.

The primary endpoints of primary patency and reduction in SBP are powered at 87% and 92%, respectively, in order to maintain the overall trial power at 80% to meet both endpoints.

5.6 Derivation of the performance goal for patency

The Performance Goal was derived from a thorough literature review of trials with renal bare metal stent placement. Trials were identified by means of a PUBMED search of the English-language medical literature from 1991 to 2010. When available, the following data were extracted: author and year of publication, number of subjects, number of arteries, restenosis definition, evaluation method, duration of follow-up, and restenosis rate.

Of the 30 articles reviewed, 9 articles were excluded from the Performance Goal based on the following: results from studies with data collection from the 1980's which is not representative of today's standard of care, definitions used for restenosis were outside of our trial design, follow-up occurred at times outside of our assessments, and results from studies not yet published.

The weighted mean restenosis rate from these trials is 21.4%. Assuming a restenosis rate for PTRA of 40% as noted in Carr based on the analysis of 6 trials with PTRA and also used by Rocha-Singh, a restenosis rate of 30% leading to a Performance Goal for primary patency set at 70%. This goal will preserve at least 50% of the difference between the currently available bare

metal stent rate and the PTRA rate, as well as adjust for a lesion set with a higher percentage stenosis (80%-100%) than previous reported studies (50%-100%).

5.7 Statistical Hypothesis

Functional endpoint

For the primary patency endpoint, the proportion of subjects-lesion (arteries) experiencing this outcome will be compared to the predefined Performance Goal of 0.70. Formally, the hypothesis to be tested is:

H_0: The incidence pPP of primary patency at 9-months is less than or equal to 0.70.

$$H_o: \quad pPP \leq 0.70$$

H_A: The incidence pPP of primary patency at 9-months is greater than 0.70.

$$H_A: \quad pPP > 0.\ 70$$

Primary Clinical Endpoint

For the SBP endpoint, the change between SBP from baseline to 9-month follow-up will be compared to a predefined Performance Goal of 10mmHg. Formally, the hypothesis to be tested is:

H_0: The mean difference between SBP at 9-months and baseline, $\mu\Delta SBP$, is less than or equal to 10mmHg.

$$H_o: \quad \mu\Delta SBP \leq 10mmHg$$

H_A: The mean difference between SBP at 9-months and baseline, $\mu\Delta SBP$, is greater than 10mmHg.

$$H_A: \quad \mu\Delta SBP > 10mmHg$$

6. CHANGES IN THE CONDUCT OF THE STUDY OR PLANNED ANALYSES

6.1 Changes in the Conduct of the Study

The enrollment for Protocol/CIP No. iCAST™ RX-ARAS-001 has been terminated after 68 subjects were screened and enrolled in the study as of November 2017. Enrolled subjects will be followed up to study completion.

6.2 Changes from the Analyses Planned in the Protocol/CIP

Due to the early termination of enrollment for Protocol/CIP No. iCAST™ RX-ARAS-001, all inferential analysis should be interpreted as descriptive and will be carried out in an exploratory manner.

The following changes were applied:

1. Missing data will not be imputed.
2. Sensitivity analysis will be limited to evaluating the primary analysis results using the per protocol population.
3. Supportive analysis of the revascularization information using a time to event approach will be performed.

7. BASELINE, EFFICACY and SAFETY EVALUATIONS

7.1 Schedule of Evaluation

Table 2 SCHEDULE OF EVENTS

	(-14)-Days (Medical Documentation Screening period) [7]<br>Prior to Procedure	(-7)-Days[7]<br>Prior to Procedure	Day 0<br>Procedure	30-Days (+14/-7 Days)<br>Office Visit	6-Months (+/-30 Days)<br>Phone Call	9-Months (+/-30 Days)<br>Office Visit	12-Months (+/- 30 Days)<br>Office Visit	18-Months (+/-30 Days)<br>Phone Call	24-Months (+/- 60 Days)<br>Office Visit	36-Months (+/- 60 Days)<br>Office Visit
**Informed Consent**	X									
**General Entry Criteria**	X									
**Angiographic Entry Criteria**			X							
**Demographic s and Medical History**	X									
**Weight (Kg)/ Height (cm)**	X									
**Vital Signs**	X		X	X		X	X		X	X
**BP Assessment[1]**	X[1]	X[1]	X	X		X	X		X	X
**Laboratory Assessments[2]**	X[2]		X[2]	X[2]		X[2]				
**Test for Proteinuria[3]**	X[3]									
**Physical Exam**	X		X	X		X	X		X	X
**Pregnancy Test[4]**	X[4]		X[4]							
**iCAST™ RX**			X							

	**(-14)-Days (Medical Documentation Screening period)** [7]<br>Prior to Procedure	**(-7)-Days**[7]<br>Prior to Procedure	**Day 0**<br>Procedure	**30-Days (+14/-7 Days)**<br>Office Visit	**6-Months (+/-30 Days)**<br>Phone Call	**9-Months (+/-30 Days)**<br>Office Visit	**12-Months (+/- 30 Days)**<br>Office Visit	**18-Months (+/-30 Days)**<br>Phone Call	**24-Months (+/- 60 Days)**<br>Office Visit	**36-Months (+/- 60 Days)**<br>Office Visit
**Stent System Placement**										
**Duplex Ultrasound**				X		X				
**Angiography**[5]			X[5]			X[5]				
**Concomitant Medications**	X	X	X	X	X	X	X	X	X	X
**Adverse Events**[6]	X	X	X	X	X	X	X	X	X	X

[1]A minimum of two office visits are required within the 14-day Medical Documentation Screening period. If the procedure is scheduled > 14 days from the first office visit, subjects should return every two weeks (+/- 3 days) for BP assessments and monitoring.

[2]Chem12 panel which includes: Glucose, Calcium, Albumin, Total protein, Sodium, Potassium, CO2, Chloride, BUN, creatinine, ALP, ALT, AST, and bilirubin.

[3] Test for proteinuria should be completed for subjects during Step 2 of the Medical Documentation Screening Period.

[4]Pregnancy test required at screening and on the day of procedure for females of childbearing potential only.

[5] All subjects must have angiography to determine percent diameter stenosis prior to treatment and at 9-months (if DUS indicates ≥ 60% stenosis).

[6] AE collection (serious and non-serious) will begin upon completion of the ICF process through the 9-month follow-up visit, thereafter only SAEs and UADEs will be recorded.

[7] Subjects with a history of > 1 month stable medication per protocol and documented SBP ≥ 155mmHg will be allowed to skip the formal Screening period. All other protocol requirements that would have normally occurred during the two week screening period must still be completed prior to day of procedure.

7.2 Time Point Algorithms

The primary and secondary endpoint outcomes will be evaluated after the last enrolled subject completes 9-months of study follow-up. In addition, secondary endpoints will be evaluated at specified time points; most of which include 30-days, 9-months, 12-months, 24-months, and 36-months. Refer to the data management plan (DMP) for details on how the data is handled at the 9-month primary endpoint and final analyses.

7.3 Handling of Missing Date/Time Data

7.3.1. Missing Date

It is preferred that the day and month of all medical history and other baseline characteristics dates will be known. If the date's day of the month is missing such that a determination of whether the event occurred within the reporting window cannot be made, the day will be imputed as follows:

- If only the month of the event is known, then the 1th day of this month will be imputed for a missing day.
- If only the year of the event is known, then the 1st of January will be imputed for a missing, day and month.
- For events occurring between two visits after enrollment, without other information available, the date in the middle of these visits will be used.

7.3.2. Missing Time

For outputs require date and time in calculation, if time is missing or incomplete, the following will be applied:

- If hour and minute are both missing, then the 12:00pm (24hr clock) of the day will be imputed.
- If only minute is missing, the 30th minute of the hour will be imputed.

7.4 Visit Windows

The time schedule described in the protocol for each scheduled activity will be followed as closely as possible. The protocol allowable visit windows in Table 3.1 below will be utilized for analysis purposes. All scheduled visits will be windowed according to the table below.

Table 3.1: Primary Visit Windows

Protocol Visit	Target Day	Protocol allowable window (days)
Screening I (Day -14)	-14	-
Screening II (Day -7)	-7	-
Baseline (Day 0)	0	-
Visit 1 (Day 30)	30	+/- 7 (23 – 44)
Visit 2 (6-Months)	182	+/- 30 (152 – 212)
Visit 3 (9-Months)	273	+/- 30 (243 – 303)
Visit 4 (12-Months)	365	+/- 30 (335 – 395)
Visit 5 (18-Months)	575	+/- 30 (545 – 605)
Visit 6 (24-Months)	730	+/- 60 (670 – 790)
Visit 7 (36-Months)	1095	+/- 60 (1035 – 1155)

If a subject has more than one assessment occurring in the same analysis visit window, the data from the visit closest to the scheduled (target) study day will be used for summaries. If two assessments have the same distance from the scheduled study day, the assessment after the scheduled study day will be used.

If two assessments both occur on the protocol-specified day, and both are valid, then the later one will be summarized. All assessments will be provided in the data listings.

The 9-month primary endpoint analysis will be based on the protocol allowable visit windows. Supportive analysis of the primary efficacy endpoint will be performed using a time to event analysis.

7.5 Baseline Assessments

- Inclusion/exclusion criteria

- Demographics (age, gender, race/ethnicity)
- Collection of targeted medical history
- Collection of focused anti-hypertensive medical history
- Height/weight
- Vital signs
- Collection of blood pressure
- Laboratory assessments:
  - Chem12 Panel which includes: Glucose, Calcium, Albumin, Total protein, Sodium, Potassium, CO_2, Chloride, BUN, creatinine, ALP, ALT, AST, and bilirubin.
  - Test for proteinuria. Urine proteinuria may be tested by dipstick. A level of > 2+ is exclusionary unless a 24 hour urine shows that the urinary protein is < 2gm/d.
  - Pregnancy Test

- Physical exam
- Collection of concomitant medications
- Collection of Adverese Events (AEs), if applicable

7.6 Endpoints, Safety Outcomes and Other Parameters

7.6.1. Primary Endpoints

Primary Patency Rate

In order to mitigate the challenges with DUS interpretation, clinical validation through independent noninvasive core laboratory and/or angiography will be performed in order to correctly validate and definitively assess percent diameter stenosis. DUS is highly reliable in identifying widely patent stents – however, correlation studies of DUS with angiography have confirmed that DUS is not as accurate when results suggest a stenosis. To lessen the risk of inaccurate readings, if a stenosis is detected in DUS (RAS > 60%) or if the DUS is non-diagnostic due to an imaging quality problem then a contrast angiogram will be used to assess the degree of restenosis of the covered stent(s) at the 9-month visit (±30 days).

The primary efficacy variable is the primary patency rate, defined as continuous patency without the occurrence of a total occlusion (complete obstruction or a closure of a passageway or vessel determined by DUS or contrast angiogram) of the original lesion, and

- without a re-intervention due to a clinically driven restenosis or occlusion to treat a partial or total occlusion of the stented segment,
- or bypass of the stented segment due to clinically-driven restenosis or occlusion

The primary patency will be calculated on a per lesion basis – for purpose of analysis in cases in which multiple lesions are covered by one stent then the stented segment will be considered as one lesion. The cumulative incidence of patency at 9 months will be computed as the number of lesions that met primary patency divided by the number of subject-lesions for the population being analyzed.

Change from Baseline in Systolic Blood Pressure

Systolic blood pressure will be assessed at each follow-up study visit. Blood pressure measurements will be obtained with a certified, calibrated, and validated equipment. Subjects will be instructed to refrain from exercise, smoking or caffeine ingestion for 30 minutes before blood pressure measurement, and measurements will be obtained after at least 5 minutes of rest. At each time point, a minimum of three (3) readings separated by 2 minutes will be averaged.

They primary clinical outcome is the improvement in systolic blood pressure (SBP) at 9-months as compared to baseline systolic blood pressure computed as follows:

SBP change from baseline = (SBP value at 9-months – SBP value at baseline)

7.6.2. Secondary Endpoints

The secondary endpoints are:

1. **The occurrence of procedure-related major adverse events (MAEs)**
   For each subject, an indicator variable which identifies if a MAE was observed will be defined. The incidence of MAE occurrence will be computed as the number of subjects with a reported MAE divided by the number of subjects in the Intent-to-treat (ITT) population.

MAEs include:

a. Procedure-or device-related occurrence of death
b. Q-Wave MI
c. Clinically driven TLR
    i. Defined as a TLR (PTA, bare metal stent or repeat covered stent deployment, or surgical bypass) due to documented recurrent hypertension from 30-days post-procedure level and/or deterioration in renal function from baseline value, associated with angiographic core laboratory adjudication of a ≥ 60% diameter covered stent restenosis.

d. Significant embolic events
    i. Defined as unanticipated kidney/bowel infarct clinically driven by symptoms of abdominal or back pain and confirmed with CT scan or open surgery, lower extremity ulceration or gangrene, or kidney failure after 30-days, 9-months, 12-months, 24-months, and 36-months.

2. Technical device delivery/deployment success

For each subject-lesion, the percent of residual stenosis will be measured via QVA by an independent core laboratory. An indicator variable that classifies success or failure of the delivery and deployment will be derived and computed as follows:

- Technical stent delivery will be defined as a success ("1") if the residual angiographic stenosis is less or equal to 30% after covered stent deployment. Otherwise, it will be defined as a failure ("0"). The percentage of subject-lesions with successful technical stent delivery/deployment will be computed by dividing the number of delivery/deployment successes by the number of subject-lesions in the ITT population.

3. Acute procedural success

For each subject-lesion, the procedural success will be derived as a composite outcome of observing technical success in the absence of a MAE prior to hospital discharge. The proportion of subject-lesions with acute procedural success will be computed as the number of subject-

lesions with technical success in the absence of a MAE divided by the total number of subject-lesions in the ITT population.

4. Target lesion revascularization (TLR)

A clinically-driven TLR is defined as a target lesion revascularization (PTA, bare metal stent or repeat covered stent deployment, or surgical bypass) due to documented recurrent hypertension from 30-days post-procedure level and/or deterioration in renal function from baseline value, and associated with angiographic core laboratory adjudication of a ≥ 60% diameter covered stent restenosis.

The percentage of TLRs will be derived as the number of subject-lesions that require a clinically-driven re-intervention divided by the number of subject-lesions in the ITT population.

5. Rate of incidental TLR

The rate of TLRs not meeting the definition of a clinically-driven TLR will be derived as the number of observed non-clinically driven TLRs divided by the total number of subject-lesions in the ITT population.

6. Improved SBP control assessed at 30-days, 9-months, 12-months, 24-months and 36-months.

The change from baseline in systolic blood pressure (SBP) at each follow-up time point will be computed as follows:

SBP change from baseline = (SBP value at follow-up – SBP value at baseline)

7. Secondary patency rate at 9-months

Secondary patency rate at 9-months after a clinically-driven TLR which restores patency after total occlusion. For each subject-lesion, an indicator variable will be derived which identifies if the lesion meets secondary patency. The incidence of secondary of patency at 9 months will be computed as the number of subject-lesions that met secondary patency divided by the number of subject-lesions for the ITT population.

8. Change in number and dosage of anti-hypertensive medications

Anti-hypertensive medications as well as the dosage will be recorded at baseline and throughout the study follow-up. The total number of anti-hypertensive medications will be computed as the sum of all available anti-hypertensive medications at each time point. Change from baseline will be computed as follows:

<u>Change from baseline in the number of antihypertensive medications</u> = (number of anti-hypertensive medications at follow-up – the number of anti-hypertensive medications at baseline)

Additionally, an indicator variable which detects if an increase in the number of antihypertensive medications (change from baseline > 0) was observed will be derived.

Similarly, the change from baseline in the anti-hypertensive medication dosage (within the same medication) will be computed as follows:

<u>Change from baseline in the antihypertensive medication dosage</u> = (Dosage of anti-hypertensive medication at follow-up – the dosage of the anti-hypertensive medication at baseline).

An indicator variable which identifies any increases in dosage (change from baseline > 0) will also be derived.

9. Renal function compared to baseline as measured by eGFR at 30-days and 9-months.

Renal function will be assessed by eGFR at the 30-days and 9-months of study follow-up. eGFR results will be estimated by the abbreviated Modification of Diet in Renal Disease(MDRD) equation : 186 x (Creat / 88.4)-1.154 x (Age)-0.203 x (0.742 if female) x (1.210 if black).

The change from baseline in eGFR will be computed as follows:

<u>Change from baseline in eGFR</u> = (eGFR result at follow-up – eGFR result at baseline)

i. In addition, an indicator variable will be derived in order to identify decreases from baseline in eGFR of >25%, >40%, >50% at any time point.

7.6.3. Safety Outcomes and Other Parameters

The main safety outcome is the incidence of subjects with any adverse event experienced after the study procedure has occurred over the totality of the study.

Other parameters collected to monitor safety and/or to derive efficacy include:

- Vital signs (systolic and diastolic blood pressure)
- Laboratory Assessments (chemistry, urine analysis, proteinuria, eGFR)
- Physical Examination (assessment of general appearance, abdomen, cardiovascular, dermatologic, genitourinary, head, eyes, ear, noes and throat (HEENT), lymph nodes, musculoskeletal, neurological, respiratory)
- Duplex Ultrasound (degree of stenosis)
- Angiography (degree of stenosis)
- Revascularization (number of revascularization, location, type)
- Concomitant medications/antihypertensive medications
- Mortality

8. STATISTICAL METHODS

8.1 General Statistical Considerations

Data collected in this study will be presented using summary tables and subject data listings. Demographic, endpoint information, safety data and other parameters will be summarized and presented for the ITT and Per-Protocol populations accordingly. Summaries for continuous and ordinal variables will include the number of observations (n), arithmetic mean, standard deviation (SD), minimum (min), maximum (max), median, and 95% confidence intervals of the means. Minima and maxima will be reported with the same precision as the raw values; means, medians and quartiles will be presented to one additional decimal place than reported in the raw values. Standard deviations will be presented to two additional decimal places than reported in the raw values.

For the time-to-event analysis, Kaplan-Meier (K-M) estimates at the indicated time points and K-M curve over time will be presented. In addition, K-M curves will be constructed for all time-to-

event analysis using Kaplan-Meier methods. Confidence intervals (CI) will be at 95% confidence level (or applicable). All p-values will be rounded to four decimal places; p-values less than 0.0001 will be presented as “<0.0001”; p-values greater than 0.9999 will be presented as “>0.9999”.

Summaries for discrete variables will include frequency counts and percentages. All percentages will be rounded to one decimal place (i.e., XX.X%). Two-sided 95% exact confidence intervals will be displayed.

Change from baseline summaries will use the procedure visit (Day 0) as the baseline measure. For measurements that have a missing value at baseline, the data prior to the procedure visit (Day 0) will be used as the baseline measurement.

In addition, the primary endpoint rate and its one-sided exact 95% CI will be presented for sub-groups (refer to section 8.9.4).

8.2 Adjustments for Covariates

Adjustment for covariates is not planned for this study.

8.3 Handling of Dropouts or Missing Data

Due to the early termination of study enrollment missing data will not be imputed as there is no need to assess the robustness of the primary analysis by evaluating the impact of missing data.

8.4 Interim Analyses and Data Monitoring

No formal statistical rule for stopping the trial will be defined in this trial and no formal interim analysis for efficacy is planned.

Periodic meetings for the independent Data Safety Monitoring Board (DSMB) will be held at which the DSMB members will evaluate interim data to determine any specific safety concerns.

The DSMB is composed of at least three members which include physicians with specialization in the field of this trial and a biostatistician. They are independent and not directly involved in the conduct of the trial. The DSMB will review the trial on a periodic basis as defined in the DSMB Charter.

Based on the safety data, the DSMB may recommend to Atrium to modify or stop the trial. All final decisions, however, regarding trial modifications, rest with Atrium.

8.5 Clinical Events Committee (CEC)

The Clinical Events Committee (CEC) is made up of physicians with specialization in the field of this trial who are not participants in the trial. The CEC is charged with the development of specific criteria used for adjudication of clinical events related to the primary endpoints in the trial.

At the onset of the trial, the CEC will develop a CEC Charter which will describe explicit rules outlining the minimum amount of data required, and the algorithm followed in order to classify a trial endpoint related clinical event. The CEC will meet regularly (at a minimum of once per year) to review and adjudicate trial endpoint related clinical events in which the required minimum data is not available. The CEC will also review and rule on all deaths that occur throughout the trial.

Once the specific criteria for clinical events and endpoints are established, the CEC will be responsible for adjudicating all clinical events when all necessary data are available.

Details regarding event adjudication are provided in the CEC Charter.

8.6 Multi-Center Studies and Pooling of Centers

Due to the early termination of study enrollment, pooling of centers will not be carried out as the patient-distribution across sites within the same geographical area might not meet pooling requirements.

8.7 Multiple Comparisons/Multiplicity

Due to early termination of study enrollment, the results from the primary and secondary efficacy analyses will be interpreted descriptively and therefore the analysis will not be adjusted to control the Type I error.

8.8 Unit of Analysis

For the primary functional endpoint of primary patency rate the unit of analysis is the atherosclerotic lesion(s). The rationale for analyzing the primary endpoint following a per lesion approach, is to avoid overestimation of the failure rate, as more lesions would be at risk for restenosis but would not be accounted for in a per subject analysis.

Since blood pressure is measured on the subject level, the primary clinical endpoint of change SBP will be evaluated at the subject level.

9. STATISTICAL ANALYSIS

9.1 Disposition of Subjects

The number of subjects screened, enrolled, and number of subjects who underwent the procedure but never received a study stent will be summarized. Also, the number of implanted subjects who completed the study, the number of implanted subjects who discontinued from the study, and the reasons for study discontinuation will also be summarized at minimum.

In addition, the number of subjects with an observed (within visit window), observed (regardless of visit window) and expected visit will be presented at each follow-up time point.

A subject listing will be provided and include at minimum the subject ID, informed consent date, procedure date, completion status, date of completion/discontinuation, last visit completed, per-protocol population exclusion, and reason for study discontinuation.

9.2 Protocol Deviations

A protocol deviation is defined as an event where the Investigator or investigational site personnel did not conduct the trial according to the protocol.

Protocol deviations were classified as major if the parameter affected one of the primary endpoints. All other protocol deviations which did not directly affect the collection or analysis of primary endpoint information were considered minor protocol deviations.

The number and percentage of subjects with major and minor protocol deviations will be summarized for all subjects in the ITT analysis population. A subject listing of all protocol deviations will be provided that includes the date of the deviation, the deviation description, the classification of whether the deviation was judged to be major or minor and an indicator for exclusions from the per protocol (PP) analysis population.

9.3 Analysis Population

Intent-to-Treat (ITT) Analysis Population: All subjects enrolled in the study will be included in the ITT analysis population. This will be the primary analysis population.

Per Protocol (PP) Analysis Population: All subjects enrolled in the study, who receive the study device as intended with no major protocol deviations will be included in the per protocol analysis population.

9.4 Demographic and Other Baseline Characteristics

The demographic and baseline characteristics of the subjects will be summarized for all subjects in the ITT analysis and the per protocol populations. All subjects enrolled will be included in a listing. Quantitative data (i.e., age, height, weight and body mass index (BMI)) will be summarized using descriptive statistics (i.e., number of subjects (N), mean, standard deviation (SD), first quartile (Q1), median, third quartile (Q3), range (minimum, maximum). Age will be reported in years and is to be calculated based on the date of informed consent using the following formula:

Age = ((informed consent date – date of birth + 1) / 365.25) truncated as an integer

Age will also be categorized as follows: <65 years and ≥65 years. Qualitative data (i.e., gender, ethnicity, race, age category) will be summarized using frequency counts and percentages.

The number and percent of subjects with abnormal physical examination findings at baseline will be summarized.

Variables to be included in the demographic and baseline characteristics summaries/listings include:

1. Age
2. Age group (>=18 - <65 years and >=65 years)
3. Gender
4. Race
5. Ethnicity
6. Number of lesions
7. Pulse
8. Oral temperature (celcius)
9. Respiratory rate (breaths/minute)
10. Systolic blood pressure (mmHg)
11. Diastolic blood pressure (mmHg)
12. Height
13. Weight
14. BMI (kg/m^2)

9.5 Targeted Medical History

A focused medical history will be reviewed and recorded for each subject at or prior to the baseline visit.
Medical history information includes:

- Previous treatment for Artherosclerosis Renal Artery Stenosis (ARAS)

- Previous bypass
- Previous surgery
- Time from most recent surgery
- Ballon Angioplasty (PTRA)
- Bare metal dtent
- Time from most recent bare metal stent
- Previous contralateral vessel
- Target vessel
- Other treatments
- History of renal insufficiency
- History of hyperlipidemia
- History of smoking
- History of diabetes mellitus
- History of Coronary Artery Disease (CAD)
- History of other artery disease (aortic disease, congenital heart disease, other)
- History of Myocardial Infarction (MI)
- Type of MI
- History of Congestive Heart Failure (CHF)
- New York Heart Association (NYHA) Classification
- Previous Percutaneous Coronary Intervention (PCI) or angioplasty
- Previous Coronary Artery Bypass Surgery (CABG)
- Time from most recent CABG
- History of arrhythmia
- History of stroke
- Time from most recent stroke
- History of Transient Ischemic Attack (TIA)
- Time from most recent TIA
- History of peripheral vascular disease
- Previous peripheral artery revascularization/surgery
- Time from most recent revascularization/surgery
- Previous procedures

- History of flash pulmonary edema
- History of fibromuscular dysplasia

In addition, the medical history information will be provided as a subject listing.

9.6 Anti-hypertensive Medical History

Anti-hypertensive medical history will capture up to three months' information prior to screening to document that reasonable and aggressive efforts were previously made to manage hypertension via a broad variety of medications that have been tried and failed. The data will be summarized and include:

- Number of subjects taking diuretics
- Number of subjects taking an Angiotensin Coverting Enzyme (ACE) inhibitor or Angiotensin Receptor Blocking (ARB) agent
- Number of subjects taking a beta blocker or calcium channel blocker
- Time from diagnosis

9.7 Procedural Characteristics

Procedure characteristics will be summarized using continuous and categorical summary statistics and will include information regarding:

- Pre-procedure percent diameter stenosis (%)
- FFR
- Pre-procedure translesional peak pressure gradient (mmHg)
- Stent placement location

A subject listing of the procedure characteristics will also be presented.

9.8 Prior and Concomitant Therapy

All medications (prescription, herbal, and over-the-counter) taken from the time of informed consent through 3 years of follow-up will be recorded in the subject's source records and transcribed into the concomitant medication section of the eCRF.

Subjects are required to receive dual anti-platelet therapy pre-procedure and for a minimum of 3 months post-procedure. The required medications for this study are listed below.

Table 5: Concomitant Medications

Timing	Medication	Procedure
Prior to Procedure	IV Heparin or low molecular weight heparin or bivalirudin	Per routine hospital practice
	Acetylsalicylic acid (ASA)	ASA 325mg loading dose should be given the day of index procedure and prior to placement of the iCAST™ RX Stent System.
	Thienopyridine	The following loading dose of one of the below thienopyridines should be given the day of index procedure and prior to placement of the iCAST™ RX Stent System.<br>Clopidogrel: ≥ 300mg<br>Prasugrel: ≥ 60mg<br>Ticlopidine: ≥ 500mg
During Procedure	IV Heparin or low molecular weight heparin or bivalirudin	Per routine hospital practice
Post Procedure	Acetylsalicylic acid (ASA)	At least 75-325mg per day for a minimum of 3 months
	Thienopyridine	The following maintenance dose of one of the below thienopyridines should be given for a minimum of 3 months or longer based on local guidelines, except when it is medically necessary to take subjects off therapy.<br>Clopidogrel: ≥ 75mg<br>Prasugrel: ≥ 10mg<br>Ticlopidine: ≥ 250mg (twice per day)

Concomitant medications will be coded using World Health Organization Drug (WHO-Drug WD 201506) and summarized to the therapeutic drug class (Anatomical Therapeutic Chemical (ATC) 4 classification) and preferred name (generic drug name).

Prior medications are those that started and stopped before exposure to study procedure; concomitant medications are all medications taken during the study period, including those started before but ongoing at the time of the study procedure. Where a medication start date is partially or fully missing, and it is unclear as to whether the medication is prior or concomitant, it will be assumed that it is concomitant.

Medications will be tabulated using frequencies and percentages. Subjects may have more than 1 medication per ATC text. At each level of subject summarization, a subject will be counted once if he/she reports one or more medications. Percentages will be based on the number of subjects in the ITT analysis population.

In addition, the number and percentage of subjects using antihypertensive concomitant medications will be summarized by pre-defined categories as recorded in the electronic case report form (eCRF) (diuretics, beta blockers/calcium channel blocker and ACE/ARBs) and presented overall and by study visit.

Use of anti-platelets medication from hospital discharge to three months will be described as the percentage of subjects taking anti-platelet medication at the 3-month follow-up visit.

A subject listing will be presented and include at minimum the medication, drug classification, start and stop dates.

9.9 Analysis of Primary Endpoints

Statistical tests relating to the primary endpoints will be one-sided with p-values less than 0.025 deemed significant.

9.9.1. Primary Patency Analysis

The primary endpoint will be the cumulative incidence of primary patency at 9-months of study computed as the number of subjects with primary patency at 9-months divided by the number of subjects in the ITT population. An exact binomial one-sided 95% confidence interval will be constructed and the upper limit compared to the Performance Goal equal to 70%. A p-value for the one-sided exact test of the difference between the observed rate and the Performance Goal will be presented. The primary analysis will be analyzed with observed data only.

A planned subgroup analysis of the primary functional endpoint will be performed (details are provided in section 9.9.4

9.9.2. Systolic Blood Pressure Analysis

The primary endpoint of SBP will be analyzed according to the Performance Goal of a decrease in SBP of 10mmHg.

The mean difference between SBP at 9-months and baseline will be compared to a fixed value of 10mmHg. The baseline and 9-month SBP data, as well as the difference in baseline will be summarized with means and standard deviations. A one-sided 95% confidence interval for the mean difference between baseline and 9-month SBP will be constructed and the upper limit will be compared to the 10mmHg Performance Goal. A p-value of the difference between the estimated SBP change from baseline and the Performance Goal will be computed using a paired t-test.

9.9.3.Sensitivity Analysis

Due to early termination of the study enrollment, assessing the robustness of the primary analysis will be performed by evaluating the primary endpoint using the per-protocol population.

9.9.4. Subgroup Analyses

Subgroup analysis of the primary functional endpoint of patency will be performed using a logistic regression model to determine if there are significant differences in the patency rates between:

- Subjects with unilateral vs. bilateral renal stenosis
- Gender
- Age (<=45 or > 45 years old)

9.9.5. Exploratory Analyses

No exploratory analyses of efficacy data are planned.

9.10 Analysis of Secondary Endpoints

Due to study enrollment termination, the secondary endpoints will not be evaluated using a gatekeeping or hierarchical approach. All secondary analyses will be performed using the ITT analysis population and missing data imputation will not be performed.

9.10.1. TLR

The secondary endpoint of clinically driven TLR will be summarized using counts and frequencies. An exact binomial one-sided 95% confidence interval will be constructed and the upper limit compared to the Performance Goal rate of 8.5%. A p-value for the one-sided test of the difference between the observed rate and the Performance Goal will be presented.

9.10.2. Changes in the number or dosage of antihypertensive medications

The secondary endpoint of changes in the number or dosage of anti-hypertensive medications as compared to baseline will be summarized using continuous summary statistics (n, median, mean, SD, min, max) for the ITT population. The average number of anti-hypertensive medications and dosage will be compared to baseline using the paired t-test.

In addition, the proportion of subjects with changes in the number or dosage of anti-hypertensive medications will be presented by counts and percents for the ITT population.

9.10.3. Other secondary endpoints

All other secondary endpoints will be evaluated in the ITT analysis population using descriptive statistics. No formal hypothesis testing will be performed. The secondary endpoints will be summarized for continuous (n, median, mean, SD, min, max), categorical (counts and percents) or time-to-event data (cumulative incidence), as appropriate.

Other secondary endpoints include:

1. Occurrence of Major Adverse Events (MAEs)
2. Acute procedural success
3. Incidental Target lesion revascularization (TLR)
4. Rate of incidental TLR
5. Improved SBP control assessed at 30-days, 9-months, 12-months, 24-months and 36-months
6. Secondary patency rate at 9-months
7. Change in number and dosage of anti-hypertensive medications
8. Renal function compared to baseline as measured by eGFR at 30-days and 9-months

9.11 Analysis of Safety and Other Parameters

All analyses below will be presented using the ITT analysis population.

9.11.1. Adverse Events

Any untoward medical occurrence observed in a subject or a clinical investigation subject administered an investigational product and that does not necessarily have a causal relationship with this treatment. An AE can therefore be any unfavorable and unintended sign

(including any clinically significant abnormal laboratory finding), symptom, or disease temporally associated with the investigational intervention, whether or not considered related to the investigational intervention. Pre-existing conditions, which worsen during a trial, are to be considered AEs. AEs will be coded using Medical Dictionary for Regulatory Activities (MedDRA) version 15.0.

Adverse events will be considered to have occurred after the study procedure in the following situations:

- If the end date of the AE is on or after the the procedure date or missing, or the event is ongoing;
- If the day, month, and year of onset are recorded, and the date of onset is the same as or later than the date of procedure
- If the day of onset is missing, and the month and year of onset are the same as or later than the month and year of the procedure;
- If the day and month of onset are missing, and the year of onset is the same as or later than the year of the procedure;
- If the day, month, and year of onset are missing.

9.11.2. Serious Adverse Events (SAE)

Any adverse experience that results in any of the following outcomes:

- Death
- Is life-threatening
- Subject hospitalization or prolongation of existing hospitalization
- A persistent or significant disability or incapacity
- Congenital anomaly or birth defect
- Important medical events that may not be immediately life-threatening or result in death or hospitalization, but may jeopardize the subject or may require intervention to prevent one of the outcomes listed in the definition above.

9.11.3. Unanticipated Adverse Device Effect (UADE)

Any serious adverse effect on health or safety, or any life-threatening problem or death caused by, or associated with, a device, if that effect, problem, or death was not previously identified in nature, severity, or degree of incidence in the Investigational Plan or Instructions For Use (IFU), or any other unanticipated serious problem associated with a device that relates to the rights, safety, or welfare of subjects.

9.11.4. Anticipated Adverse Events

Based on the literature and on clinical and commercial experience with implantation of a covered stent in human arteries,

Table 4: Anticipated AEs, includes anticipated AEs that have been identified as possible complications:

Table 4: Anticipated AEs

o Abdominal Pain<br>o Abscess<br>o Acute or sub-acute thrombosis<br>o Acute myocardial infarction<br>o Allergic reaction to stainless steel, drugs, contrast agent, or anti-platelet agents<br>o Aneurysm<br>o Arrhythmias, including Ventricular Fibrillation (VF) and Ventricular Tachycardia (VT)<br>o Arteriovenous (AV) fistula<br>o Bowel infarct<br>o Death<br>o Dialysis<br>o Emboli (air, tissue, or thrombotic) resulting in tissue ischemia or infarction<br>o Emergency surgery to correct vascular complications<br>o Extremity ischemia/amputation<br>o Fever<br>o Hemorrhage<br>o Hematoma	o Hypotension or hypertension<br>o Inadequate implantation or Intimal trauma<br>o Inadvertent exclusion of branch or accessory vessel<br>o Incision site pain or infection<br>o Injury, dissection, perforation, or rupture of the vessel<br>o Kidney infarct<br>o Myocardial infarction or ischemia<br>o Nephrectomy<br>o Pseudo-aneurysm formation<br>o Pyrogenic reaction<br>o Renal insufficiency or failure<br>o Restenosis of stented lesion<br>o Sepsis/infection<br>o Stent embolization<br>o Stent migration/stent misplacement<br>o Stroke or other cerebrovascular accident<br>o Thromboembolic event<br>o Tissue necrosis or ulceration<br>o Total occlusion<br>o Vessel spasm

The study main safety outcome is the incidence of subjects with any adverse event

observed after the study procedure over the totality of the study. An overall summary that includes the incidence of subjects with any adverse event during the entire study, the number of AEs and the number and percentage of subjects who experienced at least one AE will be presented. This summary will also include all AEs, serious AEs (SAEs), unanticipated adverse device effects (UADE), procedure-related AEs, procedure related SAEs and AEs with outcome of death.

Adverse event data summaries will be presented by System Organ Class (SOC) and Preferred Term (PT). AEs will be summarized using discrete summary statistics at the subject and event level by SOC and PT. If a subject reports the same PT multiple times within the same SOC, that PT will only be reported once within that SOC. As with the PT, if a subject reports multiple conditions within the same SOC, that SOC will only be reported once.

To count the number of subjects with any AEs, if a subject has multiple AEs coded to the same PT within the same SOC, the subject will be summarized within the maximal severity. For the summary of maximal severity, if the subject has multiple AEs of the same PT and of different grades of severity, the maximal severity will be reported. In the summary, SOC will be listed in ascending alphabetical order; PTs will be listed in order of descending frequency for all subjects within each SOC.

Separate summaries of the AEs stratified by before or after study procedure will be provided for the following categories:

- Adverse Events (AE)
- Procedure-Related AEs
- Unanticipated Adverse Device Effects (UADEs)
- Serious AEs (SAE)
- Serious Procedure Related AEs (separate for CEC adjudicated events and Investigator)
- Fatal AEs

All AEs will be presented in a subject listing. In addition, UADEs will be provided in a

separate listing.

9.11.5. Device Failures, Malfunctions and Near Incidents

Investigators are instructed to report all possible device failures, malfunctions or near incidents observed during the course of the trial, to Atrium. In addition, these incidents will be documented in the eCRF provided as follows:

- Device Failure: A device failure has occurred when the device is used in compliance with the IFU, but does not perform as described in the IFU and also negatively impacts treatment of the trial subject.
- Device Malfunction: A device malfunction occurs when an unexpected change to the device that is contradictory to the IFU is observed, which may or may not affect device performance.
- Device Misuse: Any use of the investigational device by an investigator that is contradictory to the application described in the IFU will be categorized as device misuse.

The overall number of reported device malfunctions, failures and misuses will be summarized using descriptive statistics (counts and percent).

In addition, a subject listing of the device information will be provided and include:

- Subject ID/lesion
- Lot number/Reference number
- Length/Diameter/Catheter length
- Device performance issue related to the procedure
- Type of device incident
- Type of device failure
- Problem with balloon/Type
- Adverse event associated with device performance issue

9.11.6. Clinical Laboratory Evaluations

A chemistry 12 panel which includes: Glucose, Calcium, Albumin, Total protein, Sodium, Potassium, CO_2, Chloride, BUN, creatinine, ALP, ALT, AST, and bilirubin will be collected at baseline, 30-days and 9-months of study follow-up.

The change from baseline will be defined as the follow-up visit value minus the baseline visit value. Laboratory values will also be classified as normal (if value is within normal reference range) or abnormal (if value is either below or above the normal reference range). The clinical significance of the laboratory results will be determined by the clinical investigator.

Clinical laboratory results will be converted into standard units and summarized descriptively (number of observations, standard deviation, median, min and max) using observed values and the change from baseline values.

A subject listing of the clinical laboratory parameters will be also provided and include at minimum subject ID, date/time of assessment, laboratory test, results/units, clinical significance and investigator's comments.

9.11.7. Vital Signs

Vital signs, including pulse, respiratory rate, oral temperature and systolic (SBP)/diastolic blood pressure (DBP) (mmHg) will be summarized with continuous descriptive statistics (number of observations, standard deviation, median, min and max) at baseline, follow-up visits and change from baseline (only for the SBP/DBP), where change from baseline is defined as the follow-up visit value minus the baseline visit value.

Additionally, a subject listing of the vital signs results will also be produced including at minimum date of assessment, study visit and results.

Both systolic and diastolic blood pressures will be measured in triplicate at each time point and the average of the non-missing measurements will be used.

9.11.8. Duplex Ultrasound (DUS)

Follow-up DUS of the target renal artery will be performed at 30 days and 9-months of study follow-up. The count and percentage of subjects in the ITT population with a DUS indicative of <60% or ≥ 60% stenosis will be presented at each study follow-up visit.

A subject listing with the DUS results will be presented and include at a minimum: subject ID, visit, result, date of duplex ultrasound.

9.11.9. Physical Examination

Physical Examination was determined by the investigator as normal or abnormal at each assessment. Changes from baseline will be analyzed as no change from baseline (including normal to normal and abnormal to abnormal), abnormal to normal and normal to abnormal. Changes from baseline that were determined by the investigator to be clinically significant were recorded as adverse events.

Physical exam findings will be presented in a subject listing and include at minimum date of assessment, study visit, exam (body system), findings and investigator's comments.

9.11.10. Angiographic Results and Revascularization

Angiographic Results

The degree of stenosis, baseline angiographic core lab results, procedural characteristics and post-procedure angiographics core lab results will be summarized using descriptive statistics at baseline and at the 9-months of study follow-up.

A subject listing of the angiographic results will include:

- Subject ID/Lesion
- Target artery
- Type of treatment
- Revascularization/Type of treatment required
- Guiding catheter
- Evidence of stenosis within original treatment are

- Target lesion length (mm)
- Pole to pole kidney length (mm)
- Ostial lesion
- Length of stenosis from ostium (mm)
- Minimal lumen diameter (MLD) (mm)
- Reference vessel diameter (RVD) (mm)
- Length of stent outside renal aorta (mm)
- Reference area (mm^2)
- Calcification/Location
- Aneurysm/Size (cm)
- Thrombus
- Eccentric lesion
- Ulcerate plaque
- Abrupt closure
- Spasm
- Distal embolization
- Perforation
- Thrombus in Myocardian Infarction (TIMI) flow pre/post
- Dissection/Grade
- Number of accessory renal arteries
- Per Accessory Renal Artery
  - % Stenosis
  - MLD (mm)
  - RVD (mm)

9.11.11. Revascularization

The total number of subjects with additional treatments to the target vessel will be summarized by counts and percents. In addition, the cumulative probability of target vessel revascularization by 9-months will be computed using the life-table method.

The following SAS code will be used:

```
PROC LIFETEST DATA = ADTT;
  TIME DURATION * CENSOR (0);
  STRATA TREAT;
RUN;
```

Time to revascularization is defined as the time from date of procedure until date of revascularization. For each lesion that has not undergone revascularization, it will be censored at the time of last contact date known to be alive (contacts considered in the determination of last contact date include adverse event date, vital signs, and last known alive date from Study Exit page of eCRF).

Where:

Time to Revascularization = (Date of Revascularization – Date of Procedure +1)

In order to provide visualization of the estimated probability of an event, a cumulative incidence plot of the target vessel revascularization will be presented.

A subject listing with the revascularization information will be also presented and include:

- Subject ID/Lesion
- Date of procedure
- Number of target/non-target lesions treated
- Indication for revascularization
- Lesion Location
- Lesion Type
- Pre-procedure % diameter stenosis
- % stenosis

9.11.12. Extent of Device Exposure

9.11.12.1. Extent of Exposure

The total number of study days (device exposure – measured as total days on study after procedure) will be summarized using descriptive statistics (n, mean, standard deviation, median, min and max) using the ITT analysis population.

9.11.13. Mortality

The number and percent of all cause deaths will be reported throughout the study or at the cutoff date for DSMB. The Kaplan-Meier estimate of the death free-survival curve by 9-months of study follow-up will be presented. Death-free survival is defined as the time (months) from the date of the procedure (stent placement) to the date of death of any cause. For each patient that has not known to have died, these will be censored at the time of last contact date known to be alive (contacts considered in the determination of last contact date include adverse event date, vital signs, and last known alive date from Study Exit page of eCRF).

A detailed listing of deaths will be provided and include: subject ID, date of procedure, date of death, relatedness to the study device, primary cause of death, investigator's comments.

10. COMPUTER SOFTWARE

All analyses will be performed by [redacted] using Version 9.2 or later of SAS® software. All summary tables and data listings will be prepared utilizing SAS® software.

The standard operating procedures (SOPs) of [redacted] will be followed in the creation and quality control of all data displays and analyses.

11. TABLE SHELLS AND SPECIFICATIONS

Refer to TLF mocks for a complete list of the report items.
