# Supplementary material for: De Novo Atherosclerotic Renal Artery Stenosis Covered Stent Treatment for Resistant Hypertension (ARTISAN) Results
Source: J Soc Cardiovasc Angiogr Interv. 2024 Oct 18;3(12):102400. doi: 10.1016/j.jscai.2024.102400 (PMC11725122; doi:10.1016/j.jscai.2024.102400)
Supplement: Supplemental Material 5 [file mmc5.pdf]

Demographics and Medical History Form

Welcome to the Demographics and Medical History form. Please complete the following form, then click the Save button.

Demographics

Date of Birth:

01 02
03 04
05 06
07 08
09 10
11 12
13 14
15 16
17 18
19 20
21 22
23 24
25 26
27 28
29 30
31

Jan
Feb
Mar
Apr
May
Jun
Jul
Aug
Sep
Oct
Nov
Dec

1912 1913 1914
1915 1916 1917
1918 1919 1920
1921 1922 1923
1924 1925 1926
1927 1928 1929
1930 1931 1932
1933 1934 1935
1936 1937 1938
1939 1940 1941
1942 1943 1944
1945 1946 1947
1948 1949 1950
1951 1952 1953
1954 1955 1956
1957 1958 1959
1960 1961 1962
1963 1964 1965
1966 1967 1968
1969 1970 1971
1972 1973 1974
1975 1976 1977
1978 1979 1980
1981 1982 1983
1984 1985 1986
1987 1988 1989
1990 1991 1992
1993 1994 1995
1996 1997 1998
1999 2000 2001
2002 2003 2004
2005 2006 2007
2008 2009 2010

DD-Mon-YYYY

2011 2012 2013
2014 2015

Sex: Male Female

Ethnicity: Hispanic or Latino Not Hispanic or Latino

Race (check all that apply)

American Indian or Alaskan Native

Asian

Black or African American

Native Hawaiian or other Pacific Islander

White

Other

Specify:

Height : cm

Weight : kg

Prior Medical History

Date of Atherosclerotic Renal Artery Stenosis (ARAS) diagnosis::	01<br>02<br>03<br>04<br>05<br>06<br>07<br>08<br>09<br>10<br>11<br>12<br>13<br>14<br>15<br>16<br>17<br>18<br>19<br>20<br>21<br>22	 <br><br><br><br>Jan<br>Feb<br>Mar<br>Apr<br>May<br>Jun<br>Jul<br>Aug<br>Sep<br>Oct<br>Nov<br>Dec	2015<br>2014<br>2013<br>2012<br>2011<br>2010<br>2009<br>2008<br>2007<br>2006<br>2005<br>2004<br>2003<br>2002<br>2001<br>2000<br>1999<br>1998<br>1997<br>1996<br>1995<br>1994	DD-Mon-YYYY Unknown

23 1993
24 1992
25 1991
26 1990
27 1989
28 1988
29 1987
30 1986
31 1985
1984
1983
1982
1981
1980
1979
1978
1977
1976
1975
1974
1973
1972
1971
1970

Previous treatment for ARAS: Yes No Unknown

Bypass

Surgery

Specify:

Specify date of most recent surgery:

01 02		2015 2014	
03 04		2013 2012	
05 06	Jan	2011 2010	
07 08	Feb	2009 2008	
09 10	Mar	2007 2006	
11 12	Apr	2005 2004	
13 14	May	2003 2002	
15 16	Jun	2001 2000	DD-Mon-YYYY
17 18	Jul	1999 1998	
19 20	Aug	1997 1996	
21 22	Sep	1995 1994	
23 24	Oct	1993 1992	
25 26	Nov	1991 1990	
27 28	Dec	1989 1988	
29 30		1987 1986	
31		1985 1984	

1983 1982
1981 1980
1979 1978
1977 1976
1975 1974
1973 1972
1971 1970

Balloon Angioplasty (PTRA)

Specify date of most recent PTRA:	01 02 03 04 05 06 07 08 09 10 11 12 13 14 15 16 17 18 19 20 21 22 23 24 25 26 27 28 29 30 31	Jan Feb Mar Apr May Jun Jul Aug Sep Oct Nov Dec	2015 2014 2013 2012 2011 2010 2009 2008 2007 2006 2005 2004 2003 2002 2001 2000 1999 1998 1997 1996 1995 1994 1993 1992 1991 1990 1989 1988 1987 1986 1985 1984 1983 1982 1981 1980 1979 1978 1977 1976 1975 1974 1973 1972 1971 1970	DD-Mon-YYYY

Bare metal stent

Specify date of most recent stent:	01 02 03 04 05 06 07 08 09 10 11 12 13 14 15 16 17 18 19 20 21 22 23 24 25 26	Jan Feb Mar Apr May Jun Jul Aug Sep Oct Nov Dec	2015 2014 2013 2012 2011 2010 2009 2008 2007 2006 2005 2004 2003 2002 2001 2000 1999 1998 1997 1996 1995 1994 1993 1992 1991 1990	DD-Mon-YYYY

27 28 1989 1988
29 30 1987 1986
31 1985 1984
1983 1982
1981 1980
1979 1978
1977 1976
1975 1974
1973 1972
1971 1970

Contralateral vessel

Target vessel

Other

Specify:

History of renal insufficiency:	Yes No Unknown
History of hyperlipidemia:	Yes No Unknown
History of smoking:	Current/Recent Former (stopped > 3 months ago) Never smoked Unknown
History of diabetes mellitus:	Yes No Unknown
Specify diabetes treatment:	No treatment Diet treatment only Oral agent with/without diet Insulin or any combination with insulin Other injectable medication
History of Coronary Artery Disease (CAD):	Yes No Unknown
History of other artery disease:	Yes No Unknown

Aortic disease

Congenital heart disease

Other

Specify:

History of Myocardial Infarction (MI):	Yes No Unknown
Date of most recent MI:	01 02Jan 2015 2014<br>03 04Feb 2013 2012<br>05 06Mar 2011 2010<br>07 08Apr 2009 2008<br>09 10May 2007 2006<br>11 12Jun 2005 2004<br>13 14Jul 2003 2002<br>15 16Aug 2001 2000<br>17 18Sep 1999 1998<br>19 20Oct 1997 1996<br>DD-Mon-YYYY

	21 22 Nov<br>23 24 Dec<br>25 26<br>27 28<br>29 30<br>31	1995 1994<br>1993 1992<br>1991 1990<br>1989 1988<br>1987 1986<br>1985 1984<br>1983 1982<br>1981 1980<br>1979 1978<br>1977 1976<br>1975 1974<br>1973 1972<br>1971 1970
Type of MI:	STEMI NSTEMI Unknown	
History of Congestive Heart Failure (CHF) :	Yes No Unknown	
Specify NYHA Classification:	I II III IV Unknown	
Previous Percutaneous Coronary Intervention (PCI) or angioplasty:	Yes No Unknown	
Date of most recent PCI:	01 02<br>03 04<br>05 06 Jan<br>07 08 Feb<br>09 10 Mar<br>11 12 Apr<br>13 14 May<br>15 16 Jun<br>17 18 Jul<br>19 20 Aug<br>21 22 Sep<br>23 24 Oct<br>25 26 Nov<br>27 28 Dec<br>29 30<br>31	2015 2014 2013<br>2012 2011 2010<br>2009 2008 2007<br>2006 2005 2004<br>2003 2002 2001<br>2000 1999 1998<br>1997 1996 1995<br>1994 1993 1992<br>1991 1990 1989<br>1988 1987 1986<br>1985 1984 1983<br>1982 1981 1980<br>1979 1978 1977<br>1976 1975 1974<br>1973 1972 1971<br>1970<br><br>DD-Mon-YYYY
Previous Coronary Artery Bypass Graft (CABG) surgery:	Yes No Unknown	
Date of most recent CABG:	01 02 Jan<br>03 04 Feb	2015 2014 2013<br>2012 2011 2010<br><br>DD-Mon-YYYY

05 06 Mar 2009 2008 2007
07 08 Apr 2006 2005 2004
09 10 May 2003 2002 2001
11 12 Jun 2000 1999 1998
13 14 Jul 1997 1996 1995
15 16 Aug 1994 1993 1992
17 18 Sep 1991 1990 1989
19 20 Oct 1988 1987 1986
21 22 Nov 1985 1984 1983
23 24 Dec 1982 1981 1980
25 26 1979 1978 1977
27 28 1976 1975 1974
29 30 1973 1972 1971
31 1970

History of arrhythmia: Yes No Unknown

History of stroke: Yes No Unknown

Date of most recent stroke:

01 02 2015 2014 2013
03 04 2012 2011 2010
05 06 Jan 2009 2008 2007
07 08 Feb 2006 2005 2004
09 10 Mar 2003 2002 2001
11 12 Apr 2000 1999 1998
13 14 May 1997 1996 1995
15 16 Jun 1994 1993 1992
17 18 Jul 1991 1990 1989
19 20 Aug 1988 1987 1986
21 22 Sep 1985 1984 1983
23 24 Oct 1982 1981 1980
25 26 Nov 1979 1978 1977
27 28 Dec 1976 1975 1974
29 30 1973 1972 1971
31 1970

DD-Mon-YYYY

History of Transient Ischemic Attack (TIA): Yes No Unknown

Date of most recent TIA:

01 02 Jan 2015 2014 2013
03 04 Feb 2012 2011 2010
05 06 Mar 2009 2008 2007
07 08 Apr 2006 2005 2004
09 10 May 2003 2002 2001
11 12 Jun 2000 1999 1998
13 14 Jul 1997 1996 1995
15 16 Aug 1994 1993 1992
17 18 Sep 1991 1990 1989
19 20 Oct 1988 1987 1986
21 22 1985 1984 1983

DD-Mon-YYYY

23 24 Nov 1982 1981 1980
25 26 Dec 1979 1978 1977
27 28 1976 1975 1974
29 30 1973 1972 1971
31 1970

History of peripheral vascular disease: Yes No Unknown

Previous peripheral artery revascularization/surgery: Yes No Unknown

Date of most recent revascularization/surgery:

01 02 2015 2014 2013
03 04 2012 2011 2010
05 06 Jan 2009 2008 2007
07 08 Feb 2006 2005 2004
09 10 Mar 2003 2002 2001
11 12 Apr 2000 1999 1998
13 14 May 1997 1996 1995
15 16 Jun 1994 1993 1992
17 18 Jul 1991 1990 1989
19 20 Aug 1988 1987 1986
21 22 Sep 1985 1984 1983
23 24 Oct 1982 1981 1980
25 26 Nov 1979 1978 1977
27 28 Dec 1976 1975 1974
29 30 1973 1972 1971
31 1970

DD-Mon-YYYY

Procedure:

Angioplasty

Aortobifemoral bypass

Axillofemoral bypass

Femoral-femoral bypass

Femoropopliteal bypass

Stent/Stent graft

Thrombectomy

Thrombolysis

Tibial bypass

Other

Specify:

History of Flash Pulmonary Edema: Yes No Unknown

History of Fibromuscular Dysplasia: Yes No Unknown

Other: Yes No Unknown

Specify:
